# Supplementary material for: Mapping Chilean clinical research: a protocol for a scoping review and multiple evidence gap maps
Source: BMJ Open. 2022 Jun 20;12(6):e057555. doi: 10.1136/bmjopen-2021-057555 (PMC9214414; doi:10.1136/bmjopen-2021-057555)
Supplement: Supplementary data [file bmjopen-2021-057555supp001.pdf]

Appendix A. Search strategy for a scoping review protocol for trends in Chilean clinical research

MEDLINE Ovid search strategy

Ovid MEDLINE(R) and Epub Ahead of Print, In-Process, In-Data-Review & Other Non-Indexed Citations, Daily and Versions(R) <1946 to June 17, 2021>

#1	Chile.ia,in.
#2	exp Chile/
#3	((Hospital or Clinic* or funda* or universi*) adj3 (Arica or Tarapaca or Antofagasta or Atacama or Coquimbo or Valparaiso or "Metropolitana de Santiago" or O Higgins or Maule or nuble or Biobio or Araucania or Los Rios or Los Lagos or Aysen or Magallanes)).ia,in.
#4	1 or 2 or 3
#5	randomized controlled trial.pt.
#6	controlled clinical trial.pt.
#7	randomized.ab.
#8	placebo.ab.
#9	drug therapy.fs.
#10	randomly.ab.
#11	trial.ab.
#12	groups.ab.
#13	5 or 6 or 7 or 8 or 9 or 10 or 11 or 12
#14	exp animals/ not humans.sh.
#15	13 not 14
#16	4 and 15
#17	Meta-Analysis as Topic/
#18	meta analy$.tw.
#19	metaanaly$.tw.

#20	Meta-Analysis/
#21	(systematic adj (review$1 or overview$1)).tw.
#22	exp Review Literature as Topic/
#23	17 or 18 or 19 or 20 or 21 or 22
#24	cochrane.ab.
#25	embase.ab.
#26	(psychlit or psyclit).ab.
#27	(psychinfo or psycinfo).ab.
#28	(cinahl or cinhal).ab.
#29	science citation index.ab.
#30	bids.ab.
#31	cancerlit.ab.
#32	24 or 25 or 16 or 27 or 28 or 29 or 30 or 31
#33	reference list$.ab.
#34	bibliograph$.ab.
#35	hand-search$.ab.
#36	relevant journals.ab.
#37	manual search$.ab.
#38	33 or 34 or 35 or 36 or 37
#39	selection criteria.ab.
#40	data extraction.ab.
#41	39 or 40
#42	Review/
#43	41 and 42
#44	Comment/
#45	Letter/

#46	Editorial/
#47	animal/
#48	human/
#49	47 not (47 and 48)
#50	44 or 45 or 46 or 49
#51	23 or 32 or 38 or 43 or 50
#52	4 and 51
#53	16 or 52
#54	limit 53 to yr=2000-2021

#1	exp cohort studies/
#2	cohort$.tw.
#3	exp Observational Study/
#4	observational.ab,ti.
#5	exp Cross-Sectional Studies/
#6	Prevalence Stud*.ti,ab.
#7	Cross Sectional*.ti,ab.
#8	descriptive.mp.
#9	Case.ti,ab. AND (report*.ti,ab. OR case serie*.ti,ab.)
#10	exp Diagnosis/
#11	Diagnos.ti,ab
#12	1 or 2 or 3 or 4 or 5 or 6 or 7 or 8 or 9 or 10 or 11 or 12
#13	Chile.ia,in.
#14	exp Chile/
#15	((Hospital or Clinic* or funda* or universi*) adj3 (Arica or Tarapaca or Antofagasta or Atacama or Coquimbo or Valparaiso or "Metropolitana de Santiago" or O Higgins or Maule or nuble or Biobio or Araucania or Los Rios or Los Lagos or Aysen or Magallanes)).ia,in.

#16	13 or 14 or 15
#17	12 and 16
#18	limit 17 to yr=2000-2021

Appendix B. Data sources for complimentary hand search strategy.

1) ANID repository (http://repositorio.conicyt.cl/). Repository of the National Agency for Research and Development that contains research and innovation projects financed by this agency.

2) SciELO (https://scielo.org/es/). Scielo contains the full texts of Chilean Open Access scientific journals, from all areas of knowledge, including peer-reviewed scientific research.
3) Bibliographic catalogues of the Chilean universities and DIBAM (http://www.bncatalogo.gob.cl). This is a unified catalogue of all public institutions in Chile (public libraries, archives and museums) that includes bibliographic information on all national print journals.

4) Bibliographic and National Repository of the Ministry of Health of Chile (http://www.repositoriodigital.minsal.cl/). It contains all the research developed by the Ministry, including clinical practice guidelines, primary studies, population statistics, and other documents.

5) Directory of Open Access Journals (https://doaj.org/). Independent index containing peer-reviewed open access journals.

6) Latin American Repositories Network (http://repositorioslatinoamericanos.uchile.cl). Provides access to full-text electronic publications located in different repositories in Latin American countries.

7) Portal of academic journals of the Universidad de Chile (https://revistas.uchile.cl/); Chilean Academic Journals provides open access to more than 300 publications published by universities, scientific societies, government agencies and Non-Government Organisations.

8) Google Scholar (https://scholar.google.com/).

9) PROSPERO (https://www.crd.york.ac.uk/prospero/).

10) World Health Organization International Clinical Trials Registry Platform (ICTRP, www.who.int/trialsearch/).

11) ClinicalTrials.gov (http://clinicaltrials.gov)

Appendix C. Potentially eligible and excluded conditions/programs from the General Regime of the Health Guarantees Act (*Garantías Explícitas en Salud*, GES).

Appendix C.1. Potentially eligible conditions from GES, in descending order according to Global Burden of Disease 2019 (GBD 2019) Disability-Adjusted Life Years (DALYs).

GES condition	Matching GBD 2019 cause (age groups)	DALYs, number (CI%95, upper to lower)
Myocardial infarction	Ischemic heart disease (all ages)	214819.57 (227068.320701352 to 0)
Type 2 diabetes	Diabetes mellitus type 2 (all ages)	170569.880724338 (213283.001985072 to 0.192722088496509)
Depression in people aged 15 years and over	Depressive disorders (20 plus)	121414.377867517 (169061.491027657 to 2.2253601218192E-06)
Chronic kidney disease stage 4 and 6	Chronic kidney disease (all ages)	101733.722907926 (110880.123710307 to 208.525669506703)
Ischemic stroke in people aged 15 years and over	Ischemic stroke (20 plus)	100161.760973747 (109460.558336013 to 94.57213560342)
Stomach cancer	Stomach cancer (all ages)	85929.065773134 (91647.4644054603 to

		1.26268744062835)
Lung cancer	Tracheal, bronchus, and lung cancer (all ages)	83674.4888793253 (88943.9075435297 to 0.000258752006588802)
Chronic obstructive pulmonary disease (outpatient management)	Chronic obstructive pulmonary disease (all ages)	83190.7630904602 (96438.3426616626 to 0.000164947566358871)
Alzheimer's disease and other dementias	Alzheimer's disease and other dementias (all ages)	72825.9715672292 (154958.475973897 to 4.10904084133898)
Colorectal cancer in people aged 15 years and over	Colon and rectum cancer (20 plus)	70756.6453332632 (75584.7656820732 to 1.65874626595222)
Hip and/or knee osteoarthritis, mild or moderate, in people aged 55 years and over (medical management)	Osteoarthritis (55 plus)	55576.8772705614 (112169.901045112 to 1.84635512150662)
Prevention of preterm birth	Neonatal preterm birth (all ages)	51479.7300991547 (63435.89308863 to 0.158666035639263)
Chronic hepatitis C	Cirrhosis and other chronic liver diseases due to hepatitis C (all ages)	50969.2141174526 (65323.6655151894 to 0.000549020300847728)
Breast cancer in people aged 15 years and over	Breast cancer (20 plus)	47006.5369289545 (51554.507927785 to 16.1265082764972)
Prostate cancer in people aged 15 years and over	Prostate cancer (20 plus)	45854.8719687154 (54664.4754416884 to 2.59747447089968)
Schizophrenia	Schizophrenia (all ages)	42501.3194923616

		(56297.8169798092 to 0.240749074074181)
Bipolar disorder in people aged 15 years and over	Bipolar disorder (20 plus)	38096.1151342666 (58487.7573236035 to 8.43274832801397E-06)
Community-acquired pneumonia in people aged 65 years and over (outpatient management)	Lower respiratory infections (65 to 89 years)	34844.453660895 (38946.6963391689 to 1.10402107319051)
Asthma in people aged 15 years and over	Asthma (20 plus)	32694.2377867036 (45713.8574539779 to 0.116280132000234)
Secondary subarachnoid hemorrhage to rupture of brain aneurysms	Subarachnoid hemorrhage (all ages)	28635.4404153411 (31612.3869284669 to 1089.40824984095)
Human Immunodeficiency Virus and Acquired Immunodeficiency Syndrome (HIV/AIDS)	HIV/AIDS (all ages)	27181.8681774182 (32191.0340641685 to 29.3017079071855)
Cervical cancer	Cervical cancer (all ages)	25200.4297936711 (30005.252368359 to 0.000474071744494449)
Non-refractory epilepsy in people aged 15 years and older	Idiopathic epilepsy (20 plus)	24620.5412700062 (41485.3459737919 to 3501.83306715692)
Kidney cancer in people aged 15 years and older	Kidney cancer (20 plus)	23991.4065239036 (26418.6543665931 to 534.114744605753)
Leukemia in people aged 15 years and older	Leukemia (20 plus)	21325.5560639148 (22901.6129356555 to 0.000813097105958376)

Parkinson’s disease	Parkinson's disease (all ages)	20331.9233118182 (22034.4067968826 to 147.770845873412)
Operable congenital heart defect in people younger than 15 years	Congenital heart anomalies (0 to 14)	15978.961606000155 (22470.79582735375 to 10367.285436039341)
Epithelial ovarian cancer	Ovarian cancer (20 plus)	15600.3173555778 (17963.5312380173 to 1407.16245468356)
Rheumatoid arthritis	Rheumatoid arthritis (all ages)	14399.6554552725 (18512.9913244069 to 0.00362617834828417)
Primary tumors of the central nervous system in people aged 15 years and older	Brain and central nervous system cancer (20 plus)	14245.6107807219 (16062.6268023673 to 133.432208773944)
Multiple myeloma in people aged 15 years and older	Multiple myeloma (20 plus)	13895.9089272371 (15829.801624441 to 9.75441705033119)
Chronic hepatitis due to hepatitis B virus	Cirrhosis and other chronic liver diseases due to hepatitis B (all ages)	13680.7698238002 (19065.5815024126 to 0.0085089965860338)
Bladder cancer in people aged 15 years and older	Bladder cancer (20 plus)	12707.3764274785 (14072.9882127301 to 3054.38153002352)
Cancer in children under the age of 15 years	Neoplasms (0 to 14)	12303.3788104734 (14469.5035669086 to 113.905690992672)
Total hip endoprothesis for people aged 65 years and older and arthrosis of the hip with severe functional limitations	Osteoarthritis hand (65 to 89 years)	10568.6092353729 (21500.8167895118 to 0)

Cataract surgery	Cataract (all ages)	9770.85045117638 (13317.247585107 to 3232.36904442194)
Moderate and acute bronchial asthma in children under the age of 15 years	Asthma (0 to 14)	7963.77834457529 (13656.3525907285 to 51.6514027019469)
Type I diabetes mellitus	Diabetes mellitus type 1 (all ages)	7429.36242517253 (9651.21392599779 to 0)
Testicular cancer in people aged 15 years and older	Testicular cancer (20 plus)	6425.06386606812 (7673.24210673214 to 0)
Refractory epilepsy in children between the ages of 1 and 15 years	Idiopathic epilepsy (0 to 14)	5569.06914520869 (11130.3966112371 to 105.052591863323)
Heavy consumption or low to moderate risk of addiction to alcohol and drugs in people under the age of 20 years	Substance use disorders (<20 years)	5312.55244366324 (7461.50867463031 to 698.314690531639)
Treatment for the eradication of *Helicobacter pylori*	Gastritis and duodenitis (all ages)	3833.44230358697 (5407.26107597058 to 31.0664601228219)
Differentiated and medullary thyroid cancer in people aged 15 years and older	Thyroid cancer (20 plus)	3524.95356842812 (3925.21814487822 to 0)
Treatment for benign prostatic hyperplasia in symptomatic people	Benign prostatic hyperplasia (all ages)	3136.15726142161 (4876.83538193793 to 0.00194715937414714)
Surgical treatment for chronic lesions of the mitral and tricuspid valves in people aged 15 years and older	Non-rheumatic degenerative mitral valve disease (20 plus)	2321.91890136389 (2772.42279203503 to 0.00640083277215629)
Refractive errors in people aged 65	Refraction disorders (65 to 89 years)	2267.63302256029

years and older		(3291.72011313406 to 4699.20120851181)
Relapsing-remitting multiple sclerosis	Multiple sclerosis (all ages)	2192.67301330785 (2860.98207134884 to 0.000916848432839514)
Cholecystectomy for the prevention of gallbladder cancer in people between the ages of 35 and 49 years	Gallbladder and biliary diseases (25 to 49)	2061.70765625961 (2857.83007158215 to 7.89297979047496)
Outpatient care for Acute Respiratory Infection (ARI) in children under the age of 5 years	Upper respiratory infections (under 5)	1783.98509162834 (2869.84870415435 to 53.7737581195539)
Cleft lip and palate	Orofacial clefts (0 to 14)	66.8835836316003 (109.496663940256 to 17707.9341422313)

Appendix C.2. Excluded GES conditions

GES condition	Reason for exclusion
Primary or essential arterial hypertension in people aged 15 years and older	Matching condition not found in GBD 2019 data
Hemophilia	Matching condition not found in GBD 2019 data
Cystic fibrosis	Matching condition not found in GBD 2019 data
Juvenile idiopathic arthritis	Matching condition not found in GBD 2019 data
Hypothyroidism in people aged 15 years and older	Matching condition not found in GBD 2019 data.
Systemic lupus erythematosis	Matching condition not found in GBD 2019 data
Bilateral hearing loss in people aged 65 years and older requiring hearing aids	Matching condition not found in GBD 2019 data
Diabetic retinopathy	Matching condition not found in GBD 2019 data
Nontraumatic rhegmatogenous retinal detachment	Matching condition not found in GBD 2019 data
Scoliosis surgery for people under the age of 25 years	Matching condition not found in GBD 2019 data
Osteosarcoma in people aged 15 years and older	Matching condition not found in GBD 2019 data
Strabismus in children under the age of 9 years	Matching condition not found in GBD 2019 data
Hip dysplasia	Matching condition not found in GBD 2019 data
Treatment for moderate, severe and profound hearing loss in children under four	Matching condition not found in GBD 2019 data
Serious polytrauma	GES condition does not match with a unique cause in GBD 2019 data
Lymphomas in people aged 15 years and older	GES condition does not match with a unique cause in GBD 2019 data

Spinal dysraphism	Matching condition not found in GBD 2019 data
Surgical treatment of chronic lesions of the aortic valve in people aged 15 years and older	Matching condition not found in GBD 2019 data
Surgery for herniated nucleus pulposus	Matching condition not found in GBD 2019 data
Severe burns	Matching condition not found in GBD 2019 data
Serious eye trauma	Matching condition not found in GBD 2019 data
Impulse and conduction disorders in people aged 15 years and older who require a pacemaker	Matching condition not found in GBD 2019 data
Respiratory distress syndrome in the newborn	Matching condition not found in GBD 2019 data
Bilateral sensorineural hearing loss in premature babies	Matching condition not found in GBD 2019 data
Bronchopulmonary dysplasia in premature babies	Matching condition not found in GBD 2019 data
Retinopathy in premature babies	Matching condition not found in GBD 2019 data
Moderate and serious traumatic brain injury	Matching condition not found in GBD 2019 data

Appendix C.3. Excluded GES programs

Orthosis (or technical help) for people aged 65 years and older
Pain relief and palliative care for advanced cancer
Analgesia in childbirth
Integral dental health care for girls and boys aged 6 years old
Outpatient treatment for odontological emergencies
Integral dental health care for 60 year old adults
Integral dental health care for pregnant women
Secondary prevention of end-stage chronic kidney disease
