# Supplementary material for: Mapping Chilean clinical research: a protocol for a scoping review and multiple evidence gap maps
Source: BMJ Open. 2022 Jun 20;12(6):e057555. doi: 10.1136/bmjopen-2021-057555 (PMC9214414; doi:10.1136/bmjopen-2021-057555)
Supplement: Author's manuscript [file bmjopen-2021-057555.draft_revisions.pdf]

BMJ Open is committed to open peer review. As part of this commitment we make the peer review history of every article we publish publicly available.

When an article is published we post the peer reviewers' comments and the authors' responses online. We also post the versions of the paper that were used during peer review. These are the versions that the peer review comments apply to.

The versions of the paper that follow are the versions that were submitted during the peer review process. They are not the versions of record or the final published versions. They should not be cited or distributed as the published version of this manuscript.

BMJ Open is an open access journal and the full, final, typeset and author-corrected version of record of the manuscript is available on our site with no access controls, subscription charges or pay-per-view fees (http://bmjopen.bmj.com).

If you have any questions on BMJ Open's open peer review process please email info.bmjopen@bmj.com

BMJ Open

Mapping Chilean clinical research: a protocol for a scoping review and multiple evidence gap maps

Journal:	*BMJ Open*
Manuscript ID	bmjopen-2021-057555
Article Type:	Protocol
Date Submitted by the Author:	20-Sep-2021
Complete List of Authors:	Pérez-Bracchiglione, Javier; Universidad de Valparaíso, Interdisciplinary Centre for Health Studies (CIESAL)<br>Meza, Nicolás; Universidad de Valparaíso<br>Escobar Liquitay, Camila Micaela; Instituto Universitario Hospital Italiano de Buenos Aires, Research Department<br>Franco, Juan Victor Ariel; Hospital Italiano de Buenos Aires, Instituto Universitario; Instituto Universitario Hospital Italiano<br>Munoz, Sergio; Universidad de La Frontera, Public Heath<br>Urrutia, Gerard; Iberoamerican Cochrane Centre<br>Madrid, Eva; Universidad de Valparaiso, Escuela de Medicina
Keywords:	PUBLIC HEALTH, STATISTICS & RESEARCH METHODS, HEALTH SERVICES ADMINISTRATION & MANAGEMENT

SCHOLARONE™
Manuscripts

Mapping Chilean clinical research: a protocol for a scoping review and multiple evidence gap maps

Authors:

Javier Pérez-Bracchiglione
Interdisciplinary Centre for Health Studies (CIESAL), Universidad de Valparaíso, Viña del Mar, Chile
https://orcid.org/0000-0001-8738-2184

Nicolás Meza
Interdisciplinary Centre for Health Studies (CIESAL), Universidad de Valparaíso, Viña del Mar, Chile
https://orcid.org/0000-0001-9505-0358

Juan Victor Ariel Franco
Research Department, Instituto Universitario Hospital Italiano de Buenos Aires, Buenos Aires, Argentina
https://orcid.org/0000-0003-0411-899X

Camila Micaela Escobar-Liquitay
Research Department, Instituto Universitario Hospital Italiano de Buenos Aires, Buenos Aires, Argentina
https://orcid.org/0000-0002-2903-6870

Sergio Muñoz
Department of Public Health - CIGES, Faculty of Medicine, Universidad de La Frontera, Temuco, Chile
https://orcid.org/0000-0001-8383-6599

Gerard Urrútia
Iberoamerican Cochrane Centre - Sant Pau Biomedical Research Institute (IIB Sant Pau) - CIBERESP, Barcelona, Spain
https://orcid.org/0000-0002-8850-0960

Eva Madrid
Interdisciplinary Centre for Health Studies (CIESAL), Universidad de Valparaíso, Viña del Mar, Chile
https://orcid.org/0000-0002-8095-5549

Corresponding author: Nicolás Meza
Angamos 655 Edificio R2 Oficina 1107, Reñaca, Viña del Mar, Chile
nicolas.meza@uv.cl

Word count: 3248

For peer review only

Abstract

Introduction: Clinical research broadly aims to influence decision making in order to promote appropriate healthcare. Funding agencies should prioritise research projects according to needed research topics, methodological and cost-effectiveness considerations, and expected social value. In Chile, there is no local diagnosis regarding recent clinical research that might inform prioritisation for future research funding. This research aims to comprehensively identify and classify Chilean health research studies, elaborating evidence gap maps for the most burdensome local conditions.

Methods and analysis: We will search in electronic databases (MEDLINE, Embase, PsycINFO, CINAHL, LILACS, and WoS) and perform hand searches to retrieve, identify, and classify health research studies conducted in Chile or by authors whose affiliations are based in Chile, from 2000 onwards. We will elaborate evidence matrices for the 20 conditions with the highest burden in Chile (according to the Global Burden of Disease 2019) selected from those defined under the General Regime of the Health Guarantees Act. To elaborate the evidence gap maps, we will consider prioritised interventions and core outcome sets. To identify knowledge gaps and estimate research waste, we will contrast these gap maps with the available international evidence of high or moderate certainty of evidence, for each specific clinical question. For this purpose, we will search systematic reviews using the GRADE approach.

Ethics and dissemination: No ethical approval is required to conduct this project. We will submit our results in both peer-reviewed journals and scientific conferences. We will aim to disseminate our findings through different academic platforms, social media, local press, among others. The final results will be communicated to local funding agencies and government stakeholders.

Keywords: medical research, evidence gap map, research waste, research funding, Chile

Strengths and limitations of this study

- We aim to comprehensively search, identify and classify Chilean clinical research studies, and to elaborate evidence gap maps for the most burdensome local conditions.

For peer review only - http://bmjopen.bmj.com/site/about/guidelines.xhtml

- This project will guide local stakeholders and funding agencies on the identification of current knowledge gaps and research waste, and the establishment of future research agendas.
- We will retrieve clinical research studies, excluding descriptive, qualitative, and mixed-methods designs. We will exclude diagnostic accuracy studies as well, limiting our scope to the efficacy or effectiveness of interventions only.
- By focusing on the 20 conditions with the highest burden in Chile, we will not address the whole extent of the local-prioritised health conditions. Nevertheless, our protocol will lead to future research in this regard, either in local or other settings.

Introduction

Clinical research comprehends any investigation addressing questions about prevention, aetiology, diagnosis, treatment and prognosis of any health condition or disease. Primary clinical research and synthesised evidence are the basis for knowledge translation, decision support and implementation in clinical settings, which should ultimately guide the generation of primary research [1,2].

Good quality clinical research, which must be set on priority questions [3,4], is essential for elucidating the optimal and safest approaches to solve health challenges [5]. Clinical research has to be always aimed at informing decision making, to make healthcare more effective, less harmful, and less expensive [6]. However, knowledge generation itself faces certain limitations. Much concern exists about poor design, and deficient conduction and reporting of clinical research [7], not only because of methodological issues or its effects on scientific advancement, but by its practical implications in health decision making, at either individual or population level [8]. Glasziou and Chalmers [8] estimated that 85% of the investments for clinical research end up being avoidable research waste, which may be derived from any step along the research process, by choosing research questions with low-priority, or assessing futile outcomes [3]; conducting research whose rationale of design and methods are inadequate [9]; dismissing the full dissemination of data, causing selective report bias [10]; making research findings unusable because of inadequate reporting (i.e., scientific writing and publishing issues) [11]; and disregarding regulation, governance, and management over clinical research development [12].

Governments, policy-makers, funders and researchers should have accurate and up-to-date pictures of health evidence so that funds and resources are used in the best possible way,

For peer review only

which is attuned to the Strategy on Health Research released by the World Health Organization in 2012 [13]. Nevertheless, the use of rigorous clinical research evidence for both clinical practice and health policymaking is still limited [14–16]. The limited budgets for both clinical practice and research make prioritisation a mandatory step for funding agencies, especially governmental organisations. This prioritisation should be done mainly considering the needed research topics, methodological design, cost-effectiveness of the projects, and expected social value [17,18].

Bases of health scientific advances and funding allocation should tend towards (and be guided by) population needs and local decision-making requirements [17,18]. Although some frameworks have been proposed to evaluate research applications [19], the focus on academic background and scientific productivity during the project assessment seem to be predominant [20]. In practice, organisations summon expert panels to judge according to the aforementioned technical and academic merit measures, with heterogeneous considerations about cost, research gaps, social value or local impact[20].

The lack of a prioritised agenda in developing countries may generate a source of inequity, by letting third-party interests (i.e. global research funding, or the pharmaceutical industry from high-income countries) inhabit regional research planning [21–23], in a context of poor governance in this matter [12,24]. The strategic planning of state funding allocation in most countries can still improve the identification of local evidence gaps to be filled by new research, avoiding (and not funding) redundant research. In Chile, clinical research is funded through different government grants, in addition to other types of funding (private agencies, competitive funding, pharmaceutical or medical device industry sponsoring, international grants, etc.) [25], but there is no comprehensive local diagnosis of recent clinical research that could inform prioritisation for future research funding.

This is a protocol for a government funded project with the following objectives:

1. To identify and classify health research studies conducted in Chile or by authors whose affiliations are based in Chile.
2. To elaborate evidence maps for relevant health conditions in Chile, considering prioritised interventions and core outcome sets.
3. To populate the maps with the primary and secondary evidence and their risk of bias and to identify knowledge gaps and research waste incorporating international evidence.

For peer review only - http://bmjopen.bmj.com/site/about/guidelines.xhtml

Methods and analysis

This section describes the methods for each of the objectives.

Objective 1: Identification and classification of Chilean clinical research

We will conduct a scoping review that will be reported following the Preferred Reporting Items for Systematic Reviews and Meta-Analyses extension for scoping reviews (PRISMA-ScR) [26]. For this protocol, we followed the guidance from the PRISMA-P extension for protocols [27].

Eligibility criteria

We will consider any clinical study design, either conducted in Chile or having at least one affiliation based in Chile. We define clinical studies as those focused on a clinical health topic describing, measuring or exploring a health-related outcome in humans. Considering that our focus in the following objectives will be the highest priority conditions of the last Chilean health reform in which the explicit guaranteed health conditions were defined, we will consider studies published since 2000 onwards [28].

We will consider any descriptive, observational or experimental primary study design, including case reports, case series, cross-sectional studies, case-control studies, cohort studies, quasi-experimental studies, diagnostic or prognostic studies and clinical trials. We will also include different formats of evidence synthesis, including systematic reviews, scoping reviews, evidence maps, and overviews [29]. We will not include narrative reviews, editorials, correspondence, letters to the editor, opinion articles, conference proceedings, study protocols and preprints. We will also exclude studies whose observation units are biological samples, economic evaluations, modelling studies, validation of instruments, and ecological studies.

Search methods

Electronic search strategy

We will perform a comprehensive search in electronic databases. The search strategies will be designed to retrieve any clinical study, whether conducted in Chile or with at least one affiliation based in Chile, that has been published since 2000 to date without restriction of language or publication status.

For peer review only

We will search the following databases to be used:

1. MEDLINE vía Ovid SP
2. Embase vía Elsevier
3. PsycINFO via ProQuest SP
4. Cumulative Index to Nursing and Allied Health Literature (CINAHL) via EBSCOhost
5. Latin American and Caribbean Literature in Health Sciences (LILACS)
6. Web of Science (WoS) via Clarivate.

Supplemental **Appendix A** provides details of the electronic search strategy for MEDLINE via Ovid. For our MEDLINE search, we added a highly sensitive filter to identify randomized trials developed by the Cochrane Collaboration [30] and a validated search filter to retrieve systematic reviews developed by the Scottish Intercollegiate Guidelines Network (SIGN) [31]. The strategy proposed was peer-reviewed by another information specialist prior to implementation using the PRESS checklist [32].

Hand search strategy

Considering that research in the Latin American Region might not be adequately indexed in the aforementioned databases we will conduct complimentaryhand searches in the following information sources with the same criteria reported in the electronic search:
1) ANID repository (http://repositorio.conicyt.cl/), 2) Bibliographic and National Repository of the Ministry of Health of Chile (http://www.repositoriodigital.minsal.cl/), 3) Bibliographic catalogs of the Chilean universities and DIBAM (http://www.bncatalogo.gob.cl), 4) ClinicalTrials.gov (http://clinicaltrials.gov), 5) Directory of Open Access Journals (https://doaj.org/), 6) Google Scholar (https://scholar.google.com/), 7) Latin American Repositories Network (http://repositorioslatinoamericanos.uchile.cl), 8) Portal of academic journals of the Universidad de Chile (https://revistas.uchile.cl/), 9) PROSPERO (https://www.crd.york.ac.uk/prospero/), 10) SciELO (https://scielo.org/es/), and 11) World Health Organization International Clinical Trials Registry Platform (ICTRP, www.who.int/trialsearch/).

Study selection

Two reviewers will independently perform a title and abstract screening. A third reviewer will solve any discrepancies. We will retrieve the full text of each study applying our selection criteria in order to determine its final inclusion. Afterwards, two reviewers will screen references by full text, solving discrepancies by a third reviewer. For this process, we will use the Covidence platform [33].

For peer review only - http://bmjopen.bmj.com/site/about/guidelines.xhtml

Data extraction

Two reviewers will extract the following data from the included studies:

- Bibliographic data: full citation including list of authors, journal, and date of publication.
- Type of evidence: Primary studies or secondary studies.
- Study design: Case reports, case series, cross-sectional studies, case-control studies, cohorts, quasi-experimental studies, diagnostic studies, prognostic studies, randomised trials, systematic reviews and other forms of synthesised evidence.
- Area of study: We will characterise the retrieved articles by discipline and area of study used by the Organisation for Economic Co-operation and Development (OECD) in their category scheme [34].
- Location of the study: Chile, other country, multicentric study.
- Authors and authorships: Affiliation (based in Chilean institution or not), gender and type of authorship (main author, last author, corresponding author, working groups authorships, among others).
- Diseases or health conditions addressed by each study according to the taxonomy developed by the Global Burden Disease [35].
- Funding: We will classify the type of funding of each article, public or private, competitive funding, pharmaceutical or medical device industry sponsoring, or international grants.

We will enter the data into a data extraction form (based in Google Sheets, Google LLC)

Summary of data

We do not intend to perform a risk of bias assessment at this stage (see **Objective 3**). We will summarise the findings of each category (i.e. proportion of clinical trials, proportion of studies for each condition). Moreover we will illustrate the trends for each study design and disease category.

Objective 2: Elaboration of evidence matrices for relevant health conditions in Chile, considering prioritised interventions and core outcome sets.

We will elaborate evidence matrices for prioritised health conditions selected from those defined under the General Regime of the Health Guarantees Act (*Garantías Explícitas en Salud,* GES) that was stated in 2004 in Chile. This Regime commanded public and private

For peer review only - http://bmjopen.bmj.com/site/about/guidelines.xhtml

health providers to guarantee access, opportunity, quality and financial protection for the most relevant programs, diseases or conditions [36]. This Regime contemplates 85 programs, diseases and conditions that have been selected considering the health situation of the population, the effectiveness of the interventions, their contribution to the extension or quality of life and, when possible, their cost effectiveness [37].

We extracted the list of health conditions and excluded those defined as programs (e.g. «Orthosis (or technical help) for people aged 65 years and older» which aims to improve independence in the elderly). These conditions were initially prioritised by the Chilean government based on a local study of the global burden of disease in 2007 [38]. In consultation with the Health Ministry, we decided to prioritise the conditions based on the 2019 report by the Global Burden of Disease (GBD) initiative as it is a more up-to-date resource for decision-making [39]. GBD is a consortium of more than 3,600 researchers who collect, analyse data and provide a tool to visualise the burden and health loss of people due to hundreds of health conditions. The available data is classified by country, incorporating the prevalence, risk factors and the relative harm that each condition causes [39]. We filtered the GBD database by year (2019), location (Chile), context (cause), measure (DALYs, Disability-Adjusted Life Years), and metric (number). We distinguished causes by age groups, and used data from all causes and both sexes. We excluded GES conditions that did not match precisely enough with a GBD cause, according to the consensus of the authors. We provide the details and judgements regarding the reasons for exclusions for each GES problem in **Appendix B**. Then, we selected the 20 conditions with the highest burden in Chile. This selection closely matched the local study on burden of disease conducted in 2007 which informed decision-making during the constitution of GES [38]. See **Table 1** for the list of the top 20 conditions identified in this process.

Table 1. Prioritised conditions from the General Regime of the Health Guarantees Act (*Garantías Explícitas en Salud*, GES) according to the Global Burden of Disease (GBD) 2019 in Chile, in decreasing order by Disability-Adjusted Life Years (DALYs).

GES condition	Matching GBD cause (age group)	GBD 2019 DALYs, number (CI95%)
Myocardial infarction	Ischemic heart disease (all ages)	214819.57 (227068.32 to 0)
Type 2 diabetes	Diabetes mellitus type 2 (all ages)	170569.88 (213283.00 to 0.19)

For peer review only - http://bmjopen.bmj.com/site/about/guidelines.xhtml

Depression in people aged 15 years and over	Depressive disorders (20 plus)	121414.38 (169061.49 to 2.23E-06)
Chronic kidney disease stage 4 and 6	Chronic kidney disease (all ages)	101733.72 (110880.12 to 208.53)
Ischemic stroke in people aged 15 years and over	Ischemic stroke (20 plus)	100161.76 (109460.56 to 94.57)
Stomach cancer	Stomach cancer (all ages)	85929.07 (91647.46 to 1.26)
Lung cancer	Tracheal, bronchus, and lung cancer (all ages)	83674.49 (88943.91 to 0.0002)
Chronic obstructive pulmonary disease (outpatient management)	Chronic obstructive pulmonary disease (all ages)	83190.76 (96438.34 to 0.0002)
Alzheimer's disease and other dementias	Alzheimer's disease and other dementias (all ages)	72825.97 (154958.48 to 4.11)
Colorectal cancer in people aged 15 years and over	Colon and rectum cancer (20 plus)	70756.65 (75584.77 to 1.66)
Operable congenital heart defect in people younger than 15 years	Congenital birth defects (0 to 14)	60558.15 (79184.22 to 3.88E-06)
Hip and/or knee osteoarthritis, mild or moderate, in people aged 55 years and over (medical management)	Osteoarthritis (55 plus)	55576.88 (112169.90 to 1.85)
Prevention of preterm birth	Neonatal preterm birth (all ages)	51479.73 (63435.89 to 0.16)
Chronic hepatitis C	Cirrhosis and other chronic liver diseases due to hepatitis C (all ages)	50969.21 (65323.67 to 0.0005)
Breast cancer in people aged 15 years and over	Breast cancer (20 plus)	47006.54 (51554.51 to 16.13)
Prostate cancer in people aged 15 years and over	Prostate cancer (20 plus)	45854.87 (54664.48 to 2.60)
Schizophrenia	Schizophrenia (all ages)	42501.32 (56297.82 to 0.24)
Bipolar disorder in people aged 15 years and over	Bipolar disorder (20 plus)	38096.12 (58487.76 to 8.43E-06)

For peer review only

For peer review only - http://bmjopen.bmj.com/site/about/guidelines.xhtml

Community-acquired pneumonia in people aged 65 years and over (outpatient management)	Lower respiratory infections (65 to 89 years)	34844.45 (38946.70 to 1.10)
Asthma in people aged 15 years and over	Asthma (20 plus)	32694.24 (45713.86 to 0.12)

We will then conduct the following steps to complete the matrices that will be populated with evidence in the following objective.

Step 1: Identifying the main outcomes

The definition of main outcomes for each health condition is a complex and difficult task, considering that reported outcomes are prone to bias, and that many studies tend to report outcomes with positive or statistically significant findings [40].

For that reason, we will consider agreed standardised sets of outcomes, known as Core Outcome Sets (COS) for identifying the main outcomes. The COS represents a non-restrictive minimum set of outcomes to be assessed and reported in studies for every health condition, and they are permanently updated and revised by the COMET initiative (http://www.comet-initiative.org/). The COS includes outcomes that are most relevant to clinicians, decision makers, patients and carers. If COS are not available for an individual condition, we will build a set of outcomes based on consensus with the input of multidisciplinary experts and patients, considering the existing outcomes embedded in current GES clinical practice guidelines.

Step 2: Identifying the interventions

We will define the interventions for the rows of the matrix for each health condition considering:

- Local Clinical Practice Guidelines: The Chilean Health Ministry developed clinical practice guidelines comprehending primary, secondary and tertiary prevention and treatment for the main health conditions that are guaranteed. We will extract the main interventions from the clinical recommendations in existing guidelines.
- Those prioritised by Cochrane Review Groups and Networks: We will contact Cochrane Groups and Networks' authors in order to gather a list of priority interventions for each health condition, according to their consideration.
- Those identified by the regulatory agency: We will review the authorisations of the Institute of Public Health in Chile as the main regulatory agency in charge of the permits

For peer review only

for clinical trials, in order to classify the authorised pharmacological interventions for each health condition.

Step 3: Building up the matrices

We will create evidence gap maps for each included condition. These evidence gap maps will be framed upon grids or matrices that will consider the relevant interventions (defined in **Step 3**) in the rows, and the main outcomes (defined in **Step 2**) in the columns. These grids will be populated in each intersection with the included studies, as we detail in Objective 3 (see **Objective 3** below). We will use evimappr [41], an R [42] package for producing bubble plots, which provides an interactive display for visualising the evidence gap maps.

Objective 3: To populate the maps with local primary and secondary evidence and their risk of bias and to identify knowledge gaps and research waste incorporating international evidence.

After the completion of the tasks proposed in objective 1, we will select all the studies that might provide evidence for decision making regarding interventions, to populate each of the matrices elaborated as described in objective 2. Whenever an evidence matrix is populated with studies for a certain outcome and intervention, we will refer to it as an evidence map.

Step 1: Identifying the main study designs

From the complete set of research articles included in objective 1, we will separate all those studies with a design relevant for decision making regarding interventions:

1. Randomised controlled trials.
2. Non-randomised primary studies, including controlled clinical trials, quasi-experimental designs (e.g., interrupted time series, controlled before-after studies) and observational studies (cohort studies, case-control studies, case series, analytical cross-sectional studies).
3. Synthesised evidence relevant to interventions (systematic reviews, overviews).

Descriptive studies, qualitative or mixed-methods studies, diagnostic accuracy studies, narrative reviews, scoping reviews and evidence maps will not be considered for this objective as they do not provide evidence supporting the efficacy or effectiveness of interventions.

For peer review only

For peer review only - http://bmjopen.bmj.com/site/about/guidelines.xhtml

Step 2: Risk of bias assessment

To assess risk of bias of the included studies, we will use standardised tools for each methodological design: 1) Cochrane Risk of Bias tool for randomised clinical trials [43], 2) ROBINS-I for non-randomised primary studies [44], and 3) ROBIS for systematic reviews [45], We will skip this step in the case of overviews, since currently there is no validated tool for assessing risk of bias in this specific methodological design.

Step 3: Populating evidence gap maps

We will populate each node of the previously elaborated grids or matrices with the selected studies, considering the intervention and outcome on that node. We will represent the populated nodes with visual symbols (bubbles), with different colours according to the type of study and risk of bias, and different sizes according to the number of studies available. Once a matrix is populated with Chilean studies, we will refer to it as a 'local evidence map'.

Step 4: Developing global certainty of the evidence gap maps

We will develop a second map for each prioritised condition populated with rigorous international research (defined as the availability of high or moderate certainty of evidence, for each specific clinical question, according to the GRADE approach [46], that we will refer to as a 'global evidence map'. For this purpose we will conduct a new search aimed at identifying systematic reviews using the GRADE approach, which is currently used for developing Chilean clinical practice guidelines. We will use appropriate filters for the identification of the abovementioned reviews [31]. We will populate these maps with the degree of certainty of evidence, so each node will be classified as "High certainty" or "Moderate certainty" (reflecting that mostly there is enough evidence); and "Low certainty", "Very low certainty", or "No evidence" (reflecting that more evidence is needed).

Step 5: Contrasting global certainty of the evidence maps with local maps

Once step 4 is concluded, we will have two maps for each condition: 1) A local evidence map (populated with local Chilean research), and 2) a global certainty of the evidence map (populated with certainties of evidence from international synthesised evidence). In order to identify knowledge gaps and estimate research waste, we will contrast these two maps. Once we cross these two maps, we will classify each populated node of the local map as "adequate research" or "research waste", while each empty node will be classified as "evidence gap", according to the criteria defined in **Table 2**.

For peer review only - http://bmjopen.bmj.com/site/about/guidelines.xhtml

Table 2. Possible scenarios in the evidence gap maps.

	Global evidence map: very low to low certainty of the evidence (or no evidence)	Global evidence map: moderate to high certainty of the evidence
**Local evidence map: No local evidence**	True evidence gap	False evidence gap (unless local conditions require context-specific research)
**Local evidence map: Available local evidence**	Adequate research	Adequate research (if local evidence was published before the review)<br><br>Research waste (if local evidence was published after the review)

For peer review only

We will consider the absence of local evidence as a gap. If this gap is established in the context of low or very low certainty of evidence in the global map, or if there is no global evidence, we will consider it as a true gap. If the gap is established in the context of high or moderate certainty of evidence in the global gap, we will consider it in general as a false evidence gap.

If there is local research in the context of a low, very low certainty, or no evidence in the global map, we will consider the local research as adequate. If there is local research in the context of a high or moderate certainty of evidence in the global map, we will consider the research as adequate if it was published before the review that informed the certainty of evidence, or as research waste if it was published after this review.

Ethics and dissemination

No ethical approval is required to conduct this project. Our findings will be submitted to peer-reviewed journals, and scientific conferences. To amplify the impact of our work and to

For peer review only - http://bmjopen.bmj.com/site/about/guidelines.xhtml

accomplish knowledge translation objectives, we will disseminate the results through different platforms, including academic social media, blogs, local press, among others. At the same time, we will aim to communicate the final results to local funding agencies and government stakeholders, in order to inform the elaboration of future national research agendas.

Acknowledgments: We thank Ivan Solà from the Iberoamerican Cochrane Centre - Sant Pau Biomedical Research Institute (IIB Sant Pau) - CIBERESP (Barcelona, Spain) for their valuable support in drafting and refining our search strategy.

Authors' contributions: The study concept was developed by JP-B, NM, JVAF and EM. The manuscript of the protocol was drafted by JP-B, NM, JVAF, CME-L and EM. SM and GU developed and provided feedback for all sections of the review protocol and approved the final manuscript. The search strategy was developed by CME-L and JP-B. Study selection, data extraction, quality assessment and gap maps elaboration will be performed by all the authors. All authors reviewed and approved the final version of the manuscript.

Funding: FONDECYT Grant 1212037 from the Chilean National Agency of Research and Development (ANID).

Competing interests: None declared.

Patient consent for publication: Not required.

Provenance and peer review: Not commissioned; externally peer reviewed.

Data sharing statements: No additional data are available.

For peer review only

References

1 Vandvik PO, Brandt L, Alonso-Coello P, *et al.* Creating Clinical Practice Guidelines We Can Trust, Use, and Share. *Chest* 2013;**144**:381–9. doi:10.1378/chest.13-0746
2 Vandvik PO, Brandt L. Future of Evidence Ecosystem Series: Evidence ecosystems and learning health systems: why bother? *Journal of Clinical Epidemiology* 2020;**123**:166–70. doi:10.1016/j.jclinepi.2020.02.008
3 Chalmers I, Bracken MB, Djulbegovic B, *et al. How to increase value and reduce waste when research priorities are set*. Elsevier 2014. doi:10.1016/S0140-6736(13)62229-1
4 Dechartres A, Ravaud P. *Better prioritization to increase research value and decrease waste*. BioMed Central 2015. doi:10.1186/s12916-015-0492-3
5 Cartabellotta A, Tilson JK. The ecosystem of evidence cannot thrive without efficiency of knowledge generation, synthesis, and translation. *Journal of Clinical Epidemiology* 2019;**110**:90–5. doi:10.1016/j.jclinepi.2019.01.008
6 World Health Organization. *The WHO strategy on research for health*. World Health Organization 2012.
7 Ioannidis JPA, Greenland S, Hlatky MA, *et al. Increasing value and reducing waste in*

For peer review only - http://bmjopen.bmj.com/site/about/guidelines.xhtml

research design, conduct, and analysis. Elsevier 2014. doi:10.1016/S0140-6736(13)62227-8
8 Glasziou P, Chalmers I. Research waste is still a scandal- A n essay by Paul Glasziou and Iain Chalmers. *The BMJ* 2018;**363**:10–2. doi:10.1136/bmj.k4645
9 Ioannidis JPA, Greenland S, Hlatky MA, *et al. Increasing value and reducing waste in research design, conduct, and analysis*. Elsevier 2014. doi:10.1016/S0140-6736(13)62227-8
10 Chan A-W, Song F, Vickers A, *et al.* Increasing value and reducing waste: addressing inaccessible research. *The Lancet* 2014;**383**:257–66. doi:10.1016/S0140-6736(13)62296-5
11 Glasziou P, Altman DG, Bossuyt P, *et al.* Reducing waste from incomplete or unusable reports of biomedical research. *The Lancet* 2014;**383**:267–76. doi:10.1016/S0140-6736(13)62228-X
12 Salman RA-S, Beller E, Kagan J, *et al.* Increasing value and reducing waste in biomedical research regulation and management. *The Lancet* 2014;**383**:176–85. doi:10.1016/S0140-6736(13)62297-7
13 World Health Organization. Research and development to meet health needs in developing countries: strengthening global financing and coordination: report of the consultative expert working group on research and development: financing and coordination. 2012.
14 Brownson RC, Fielding JE, Maylahn CM. Evidence-Based Public Health: A Fundamental Concept for Public Health Practice. *Annu Rev Public Health* 2009;**30**:175–201. doi:10.1146/annurev.publhealth.031308.100134
15 Lester L, Haby MM, Chapman E, *et al.* Evaluation of the performance and achievements of the WHO Evidence-informed Policy Network (EVIPNet) Europe. In Review 2020. doi:10.21203/rs.2.21663/v1
16 van de Goor I, Hämäläinen R-M, Syed A, *et al.* Determinants of evidence use in public health policy making: Results from a study across six EU countries. *Health Policy* 2017;**121**:273–81. doi:10.1016/j.healthpol.2017.01.003
17 Ciarli T, Ràfols I. The relation between research priorities and societal demands: The case of rice. *Research Policy* 2019;**48**:949–67. doi:10.1016/j.respol.2018.10.027
18 Mutapi F. Africa should set its own health-research agenda. *Nature* 2019;**575**:567. doi:10.1038/d41586-019-03627-9
19 Tuffaha HW, Aitken J, Chambers S, *et al.* A Framework to Prioritise Health Research Proposals for Funding: Integrating Value for Money. *Appl Health Econ Health Policy* 2019;**17**:761–70. doi:10.1007/s40258-019-00495-2
20 Cassi L, Lahatte A, Rafols I, *et al.* Improving fitness: Mapping research priorities against societal needs on obesity. *Journal of Informetrics* 2017;**11**:1095–113. doi:10.1016/j.joi.2017.09.010
21 Aguilera B. Trends in clinical trials performed in Chile. *Rev méd Chile* 2021;**149**:110–8. doi:10.4067/S0034-98872021000100110
22 Atal I, Trinquart L, Porcher R, *et al.* Differential Globalization of Industry- and Non-Industry–Sponsored Clinical Trials. *PLoS ONE* 2015;**10**:e0145122. doi:10.1371/journal.pone.0145122
23 Hoffman SJ, Gunn E, Rogers Van Katwyk S, *et al.* Systematic analysis of global health research funding in Canada, 2000–2016. *Can J Public Health* 2020;**111**:80–95. doi:10.17269/s41997-019-00247-8
24 Smits P, Champagne F. Governance of health research funding institutions: an integrated conceptual framework and actionable functions of governance. *Health Res Policy Sys* 2020;**18**:22. doi:10.1186/s12961-020-0525-z
25 Agencia Nacional de Investigación y Desarrollo, Ministerio de Ciencia, Tecnología, Conocimiento e Innovación, Chile. ANID, Concursos. 2021.https://www.anid.cl/concursos/ (accessed 2 Jul 2021).
26 Tricco AC, Lillie E, Zarin W, *et al.* PRISMA Extension for Scoping Reviews (PRISMA-ScR): Checklist and Explanation. *Ann Intern Med* 2018;**169**:467–73. doi:10.7326/M18-

For peer review only - http://bmjopen.bmj.com/site/about/guidelines.xhtml

0850
27 Moher D, Shamseer L, Clarke M, *et al.* Preferred reporting items for systematic review and meta-analysis protocols (PRISMA-P) 2015 statement. *Syst Rev* 2015;**4**:1. doi:10.1186/2046-4053-4-1
28 Bastias G, Pantoja T, Leisewitz T, *et al.* Health care reform in Chile. *Canadian Medical Association Journal* 2008;**179**:1289–92. doi:10.1503/cmaj.071843
29 Franco JVA, Arancibia M, Simancas-Racines D, *et al.* Syntheses of biomedical information: narrative reviews, systematic reviews and emerging formats. *Medwave* 2018;**18**:e7354–e7354. doi:10.5867/medwave.2018.07.7354
30 Lefebvre C, Glanville J, Briscoe S, *et al.* Technical Supplement to Chapter 4: Searching for and selecting studies. In: *Cochrane Handbook for Systematic Reviews of Interventions Version 6.2 (updated February 2021)*. Cochrane 2021. www.training.cochrane.org/handbook. (accessed 3 Sep 2021).
31 SIGN. Search filters. Scottish Intercollegiate Guidelines Network (SIGN). https://www.sign.ac.uk/what-we-do/methodology/search-filters/ (accessed 3 Sep 2021).
32 McGowan J, Sampson M, Salzwedel DM, *et al.* PRESS Peer Review of Electronic Search Strategies: 2015 Guideline Statement. *Journal of Clinical Epidemiology* 2016;**75**:40–6. doi:10.1016/j.jclinepi.2016.01.021
33 Veritas Health Innovation. *Covidence*. Melbourne, Australia: www.covidence.org
34 OECD Category Scheme. http://help.prod-incites.com/inCites2Live/filterValuesGroup/researchAreaSchema/oecdCategoryScheme.html (accessed 14 Jul 2020).
35 Lozano R, Naghavi M, Foreman K, *et al.* Global and regional mortality from 235 causes of death for 20 age groups in 1990 and 2010: a systematic analysis for the Global Burden of Disease Study 2010. *The Lancet* 2012;**380**:2095–128. doi:10.1016/S0140-6736(12)61728-0
36 Superintendencia de Salud, MINSAL. Ley N° 19.966. Superintendencia de Salud, MINSAL. 2004.https://www.supersalud.gob.cl/normativa/668/w3-article-554.html (accessed 1 Sep 2021).
37 Urriola C, Infante A, Aguilera I, *et al.* La reforma de salud chilena a diez años de su implementación. *Salud Publica de Mexico* 2016;**58**:514–21. doi:10.21149/spm.v58i5.8240
38 Departamento de Epidemiología, MINSAL. Estudio Carga de Enfermedad y Carga atribuible. 2007.http://epi.minsal.cl/estudio-de-carga-de-enfermedades/ (accessed 1 Sep 2021).
39 Vos T, Lim SS, Abbafati C, *et al.* Global burden of 369 diseases and injuries in 204 countries and territories, 1990–2019: a systematic analysis for the Global Burden of Disease Study 2019. *The Lancet* 2020;**396**:1204–22. doi:10.1016/S0140-6736(20)30925-9
40 Dwan K, Altman DG, Arnaiz JA, *et al.* Systematic Review of the Empirical Evidence of Study Publication Bias and Outcome Reporting Bias. *PLoS ONE* 2008;**3**:e3081. doi:10.1371/journal.pone.0003081
41 Haddaway N, Keenan C. *evimappr: an R package for creating bubble plots for evidence maps.* 2021. https://github.com/nealhaddaway/evimappr/
42 R Core Team. *R: A language and environment for statistical*. 2021. https://www.R-project.org
43 Higgins JPT, Altman DG, Gotzsche PC, *et al.* The Cochrane Collaboration's tool for assessing risk of bias in randomised trials. *BMJ* 2011;**343**:d5928–d5928. doi:10.1136/bmj.d5928
44 Sterne JAC, Savović J, Page MJ, *et al.* RoB 2: a revised tool for assessing risk of bias in randomised trials. *BMJ* 2019;:l4898. doi:10.1136/bmj.l4898
45 Whiting P, Savović J, Higgins JPT, *et al.* ROBIS: A new tool to assess risk of bias in systematic reviews was developed. *Journal of Clinical Epidemiology* 2016;**69**:225–34. doi:10.1016/j.jclinepi.2015.06.005
46 Guyatt G, Oxman AD, Akl EA, *et al.* GRADE guidelines: 1. Introduction—GRADE

For peer review only - http://bmjopen.bmj.com/site/about/guidelines.xhtml

evidence profiles and summary of findings tables. *Journal of Clinical Epidemiology* 2011;**64**:383–94. doi:10.1016/j.jclinepi.2010.04.026

For peer review only

Appendix A. Search strategy for a scoping review protocol for trends in Chilean clinical research

MEDLINE Ovid search strategy

Ovid MEDLINE(R) and Epub Ahead of Print, In-Process, In-Data-Review & Other Non-Indexed Citations, Daily and Versions(R) <1946 to June 17, 2021>

#1	Chile.ia,in.
#2	exp Chile/
#3	((Hospital or Clinic* or funda* or universi*) adj3 (Arica or Tarapaca or Antofagasta or Atacama or Coquimbo or Valparaiso or "Metropolitana de Santiago" or O Higgins or Maule or nuble or Biobio or Araucania or Los Rios or Los Lagos or Aysen or Magallanes)).ia,in.
#4	1 or 2 or 3
#5	randomized controlled trial.pt.
#6	controlled clinical trial.pt.
#7	randomized.ab.
#8	placebo.ab.
#9	drug therapy.fs.
#10	randomly.ab.
#11	trial.ab.
#12	groups.ab.
#13	5 or 6 or 7 or 8 or 9 or 10 or 11 or 12
#14	exp animals/ not humans.sh.
#15	13 not 14
#16	4 and 15
#17	Meta-Analysis as Topic/
#18	meta analy$.tw.
#19	metaanaly$.tw.
#20	Meta-Analysis/

For peer review only

#21	(systematic adj (review$1 or overview$1)).tw.
#22	exp Review Literature as Topic/
#23	17 or 18 or 19 or 20 or 21 or 22
#24	cochrane.ab.
#25	embase.ab.
#26	(psychlit or psyclit).ab.
#27	(psychinfo or psycinfo).ab.
#28	(cinahl or cinhal).ab.
#29	science citation index.ab.
#30	bids.ab.
#31	cancerlit.ab.
#32	24 or 25 or 16 or 27 or 28 or 29 or 30 or 31
#33	reference list$.ab.
#34	bibliograph$.ab.
#35	hand-search$.ab.
#36	relevant journals.ab.
#37	manual search$.ab.
#38	33 or 34 or 35 or 36 or 37
#39	selection criteria.ab.
#40	data extraction.ab.
#41	39 or 40
#42	Review/
#43	41 and 42
#44	Comment/
#45	Letter/
#46	Editorial/
#47	animal/

For peer review only

#48	human/
#49	47 not (47 and 48)
#50	44 or 45 or 46 or 49
#51	23 or 32 or 38 or 43 or 50
#52	4 and 51
#53	16 or 52
#54	limit 53 to yr=2000-2021

#1	exp cohort studies/
#2	cohort$.tw.
#3	exp Observational Study/
#4	observational.ab,ti.
#5	exp Cross-Sectional Studies/
#6	Prevalence Stud*.ti,ab.
#7	Cross Sectional*.ti,ab.
#8	descriptive.mp.
#9	Case.ti,ab. AND (report*.ti,ab. OR case serie*.ti,ab.)
#10	exp Diagnosis/
#11	Diagnos.ti,ab
#12	1 or 2 or 3 or 4 or 5 or 6 or 7 or 8 or 9 or 10 or 11 or 12
#13	Chile.ia,in.
#14	exp Chile/
#15	((Hospital or Clinic* or funda* or universi*) adj3 (Arica or Tarapaca or Antofagasta or Atacama or Coquimbo or Valparaiso or "Metropolitana de Santiago" or O Higgins or Maule or nuble or Biobio or Araucania or Los Rios or Los Lagos or Aysen or Magallanes)).ia,in.
#16	13 or 14 or 15
#17	12 and 16
#18	limit 17 to yr=2000-2021

For peer review only

Appendix B. Potentially eligible and excluded conditions/programs from the General Regime of the Health Guarantees Act (*Garantías Explícitas en Salud*, GES).

Appendix B.1. Potentially eligible conditions from GES, in descending order according to Global Burden of Disease 2019 (GBD 2019) Disability-Adjusted Life Years (DALYs).

GES condition	Matching GBD 2019 cause (age groups)	DALYs, number (CI%95)
Myocardial infarction	Ischemic heart disease (all ages)	214819.571800329 (227068.320701352 to 0)
Type 2 diabetes	Diabetes mellitus type 2 (all ages)	170569.880724338 (213283.001985072 to 0.192722088496509)
Depression in people aged 15 years and over	Depressive disorders (20 plus)	121414.377867517 (169061.491027657 to 2.2253601218192E-06)
Chronic kidney disease stage 4 and 6	Chronic kidney disease (all ages)	101733.722907926 (110880.123710307 to 208.525669506703)
Ischemic stroke in people aged 15 years and over	Ischemic stroke (20 plus)	100161.760973747 (109460.558336013 to 94.57213560342)
Stomach cancer	Stomach cancer (all ages)	85929.065773134 (91647.4644054603 to 1.26268744062835)

For peer review only

Lung cancer	Tracheal, bronchus, and lung cancer (all ages)	83674.4888793253 (88943.9075435297 to 0.000258752006588802)
Chronic obstructive pulmonary disease (outpatient management)	Chronic obstructive pulmonary disease (all ages)	83190.7630904602 (96438.3426616626 to 0.000164947566358871)
Alzheimer's disease and other dementias	Alzheimer's disease and other dementias (all ages)	72825.9715672292 (154958.475973897 to 4.10904084133898)
Colorectal cancer in people aged 15 years and over	Colon and rectum cancer (20 plus)	70756.6453332632 (75584.7656820732 to 1.65874626595222)
Operable congenital heart defect in people younger than 15 years	Congenital birth defects (0 to 14)	60558.1526518899 (79184.2191913363 to 3.88473612239553E-06)
Hip and/or knee osteoarthritis, mild or moderate, in people aged 55 years and over (medical management)	Osteoarthritis (55 plus)	55576.8772705614 (112169.901045112 to 1.84635512150662)
Prevention of preterm birth	Neonatal preterm birth (all ages)	51479.7300991547 (63435.89308863 to 0.158666035639263)
Chronic hepatitis C	Cirrhosis and other chronic liver diseases due to hepatitis C (all ages)	50969.2141174526 (65323.6655151894 to 0.000549020300847728)
Breast cancer in people aged 15 years and over	Breast cancer (20 plus)	47006.5369289545 (51554.507927785 to 16.1265082764972)
Prostate cancer in people aged 15 years and over	Prostate cancer (20 plus)	45854.8719687154 (54664.4754416884 to 2.59747447089968)

For peer review only

Schizophrenia	Schizophrenia (all ages)	42501.3194923616 (56297.8169798092 to 0.240749074074181)
Bipolar disorder in people aged 15 years and over	Bipolar disorder (20 plus)	38096.1151342666 (58487.7573236035 to 8.43274832801397E-06)
Community-acquired pneumonia in people aged 65 years and over (outpatient management)	Lower respiratory infections (65 to 89 years)	34844.453660895 (38946.6963391689 to 1.10402107319051)
Asthma in people aged 15 years and over	Asthma (20 plus)	32694.2377867036 (45713.8574539779 to 0.116280132000234)
Secondary subarachnoid hemorrhage to rupture of brain aneurysms	Subarachnoid hemorrhage (all ages)	28635.4404153411 (31612.3869284669 to 1089.40824984095)
Human Immunodeficiency Virus and Acquired Immunodeficiency Syndrome (HIV/AIDS)	HIV/AIDS (all ages)	27181.8681774182 (32191.0340641685 to 29.3017079071855)
Cervical cancer	Cervical cancer (all ages)	25200.4297936711 (30005.252368359 to 0.000474071744494449)
Non-refractory epilepsy in people aged 15 years and older	Idiopathic epilepsy (20 plus)	24620.5412700062 (41485.3459737919 to 3501.83306715692)
Kidney cancer in people aged 15 years and older	Kidney cancer (20 plus)	23991.4065239036 (26418.6543665931 to 534.114744605753)
Leukemia in people aged 15 years and older	Leukemia (20 plus)	21325.5560639148 (22901.6129356555 to 0.000813097105958376)

Parkinson’s disease	Parkinson's disease (all ages)	20331.9233118182 (22034.4067968826 to 147.770845873412)
Epithelial ovarian cancer	Ovarian cancer (20 plus)	15600.3173555778 (17963.5312380173 to 1407.16245468356)
Rheumatoid arthritis	Rheumatoid arthritis (all ages)	14399.6554552725 (18512.9913244069 to 0.00362617834828417)
Primary tumors of the central nervous system in people aged 15 years and older	Brain and central nervous system cancer (20 plus)	14245.6107807219 (16062.6268023673 to 133.432208773944)
Multiple myeloma in people aged 15 years and older	Multiple myeloma (20 plus)	13895.9089272371 (15829.801624441 to 9.75441705033119)
Chronic hepatitis due to hepatitis B virus	Cirrhosis and other chronic liver diseases due to hepatitis B (all ages)	13680.7698238002 (19065.5815024126 to 0.0085089965860338)
Bladder cancer in people aged 15 years and older	Bladder cancer (20 plus)	12707.3764274785 (14072.9882127301 to 3054.38153002352)
Cancer in children under the age of 15 years	Neoplasms (0 to 14)	12303.3788104734 (14469.5035669086 to 113.905690992672)
Total hip endoprothesis for people aged 65 years and older and arthrosis of the hip with severe functional limitations	Osteoarthritis hand (65 to 89 years)	10568.6092353729 (21500.8167895118 to 0)
Cataract surgery	Cataract (all ages)	9770.85045117638 (13317.247585107 to 3232.36904442194)

For peer review only

Moderate and acute bronchial asthma in children under the age of 15 years	Asthma (0 to 14)	7963.77834457529 (13656.3525907285 to 51.6514027019469)
Type I diabetes mellitus	Diabetes mellitus type 1 (all ages)	7429.36242517253 (9651.21392599779 to 0)
Testicular cancer in people aged 15 years and older	Testicular cancer (20 plus)	6425.06386606812 (7673.24210673214 to 0)
Refractory epilepsy in children between the ages of 1 and 15 years	Idiopathic epilepsy (0 to 14)	5569.06914520869 (11130.3966112371 to 105.052591863323)
Heavy consumption or low to moderate risk of addiction to alcohol and drugs in people under the age of 20 years	Substance use disorders (<20 years)	5312.55244366324 (7461.50867463031 to 698.314690531639)
Treatment for the eradication of *Helicobacter pylori*	Gastritis and duodenitis (all ages)	3833.44230358697 (5407.26107597058 to 31.0664601228219)
Differentiated and medullary thyroid cancer in people aged 15 years and older	Thyroid cancer (20 plus)	3524.95356842812 (3925.21814487822 to 0)
Treatment for benign prostatic hyperplasia in symptomatic people	Benign prostatic hyperplasia (all ages)	3136.15726142161 (4876.83538193793 to 0.00194715937414714)
Surgical treatment for chronic lesions of the mitral and tricuspid valves in people aged 15 years and older	Non-rheumatic degenerative mitral valve disease (20 plus)	2321.91890136389 (2772.42279203503 to 0.00640083277215629)
Refractive errors in people aged 65 years and older	Refraction disorders (65 to 89 years)	2267.63302256029 (3291.72011313406 to 4699.20120851181)
Relapsing-remitting multiple sclerosis	Multiple sclerosis (all ages)	2192.67301330785 (2860.98207134884 to

		0.000916848432839514)
Cholecystectomy for the prevention of gallbladder cancer in people between the ages of 35 and 49 years	Gallbladder and biliary diseases (25 to 49)	2061.70765625961 (2857.83007158215 to 7.89297979047496)
Outpatient care for Acute Respiratory Infection (ARI) in children under the age of 5 years	Upper respiratory infections (under 5)	1783.98509162834 (2869.84870415435 to 53.7737581195539)
Cleft lip and palate	Orofacial clefts (0 to 14)	66.8835836316003 (109.496663940256 to 17707.9341422313)

For peer review only

Appendix B.2. Excluded GES conditions

GES condition	Reason for exclusion
Primary or essential arterial hypertension in people aged 15 years and older	Matching condition not found in GBD 2019 data
Hemophilia	Matching condition not found in GBD 2019 data
Cystic fibrosis	Matching condition not found in GBD 2019 data
Juvenile idiopathic arthritis	Matching condition not found in GBD 2019 data
Hypothyroidism in people aged 15 years and older	Matching condition not found in GBD 2019 data.
Systemic lupus erythematosis	Matching condition not found in GBD 2019 data
Bilateral hearing loss in people aged 65 years and older requiring hearing aids	Matching condition not found in GBD 2019 data
Diabetic retinopathy	Matching condition not found in GBD 2019 data
Nontraumatic rhegmatogenous retinal detachment	Matching condition not found in GBD 2019 data
Scoliosis surgery for people under the age of 25 years	Matching condition not found in GBD 2019 data
Osteosarcoma in people aged 15 years and older	Matching condition not found in GBD 2019 data
Strabismus in children under the age of 9 years	Matching condition not found in GBD 2019 data
Hip dysplasia	Matching condition not found in GBD 2019 data
Treatment for moderate, severe and profound hearing loss in children under four	Matching condition not found in GBD 2019 data
Serious polytrauma	GES condition does not match with a unique cause in GBD 2019 data
Lymphomas in people aged 15 years and older	GES condition does not match with a unique cause in GBD 2019 data
Spinal dysraphism	Matching condition not found in GBD 2019 data

For peer review only

Surgical treatment of chronic lesions of the aortic valve in people aged 15 years and older	Matching condition not found in GBD 2019 data
Surgery for herniated nucleus pulposus	Matching condition not found in GBD 2019 data
Severe burns	Matching condition not found in GBD 2019 data
Serious eye trauma	Matching condition not found in GBD 2019 data
Impulse and conduction disorders in people aged 15 years and older who require a pacemaker	Matching condition not found in GBD 2019 data
Respiratory distress syndrome in the newborn	Matching condition not found in GBD 2019 data
Bilateral sensorineural hearing loss in premature babies	Matching condition not found in GBD 2019 data
Bronchopulmonary dysplasia in premature babies	Matching condition not found in GBD 2019 data
Retinopathy in premature babies	Matching condition not found in GBD 2019 data
Moderate and serious traumatic brain injury	Matching condition not found in GBD 2019 data

Appendix B.3. Excluded GES programs

Orthosis (or technical help) for people aged 65 years and older
Pain relief and palliative care for advanced cancer
Analgesia in childbirth
Integral dental health care for girls and boys aged 6 years old
Outpatient treatment for odontological emergencies
Integral dental health care for 60 year old adults
Integral dental health care for pregnant women
Secondary prevention of end-stage chronic kidney disease

For peer review only

Mapping Chilean clinical research: a protocol for a scoping review and multiple evidence gap maps

Journal:	*BMJ Open*
Manuscript ID	bmjopen-2021-057555.R1
Article Type:	Protocol
Date Submitted by the Author:	30-Jan-2022
Complete List of Authors:	Pérez-Bracchiglione, Javier; Universidad de Valparaíso, Interdisciplinary Centre for Health Studies (CIESAL)<br>Meza, Nicolás; Universidad de Valparaíso<br>Franco, Juan Victor Ariel; Hospital Italiano de Buenos Aires, Instituto Universitario; Instituto Universitario Hospital Italiano<br>Escobar Liquitay, Camila Micaela; Instituto Universitario Hospital Italiano de Buenos Aires, Research Department<br>Munoz, Sergio; Universidad de La Frontera, Public Heath<br>Urrutia, Gerard; Iberoamerican Cochrane Centre<br>Madrid, Eva; Universidad de Valparaiso, Escuela de Medicina
**Primary Subject Heading**:	Epidemiology
Secondary Subject Heading:	Health policy, Medical publishing and peer review, Health services research
Keywords:	PUBLIC HEALTH, STATISTICS & RESEARCH METHODS, HEALTH SERVICES ADMINISTRATION & MANAGEMENT

SCHOLARONE™
Manuscripts

Mapping Chilean clinical research: a protocol for a scoping review and multiple evidence gap maps

Authors:

Javier Pérez-Bracchiglione
Interdisciplinary Centre for Health Studies (CIESAL), Universidad de Valparaíso, Viña del Mar, Chile
https://orcid.org/0000-0001-8738-2184

Nicolás Meza
Interdisciplinary Centre for Health Studies (CIESAL), Universidad de Valparaíso, Viña del Mar, Chile
https://orcid.org/0000-0001-9505-0358

Juan Victor Ariel Franco
Research Department, Instituto Universitario Hospital Italiano de Buenos Aires, Buenos Aires, Argentina
https://orcid.org/0000-0003-0411-899X

Camila Micaela Escobar-Liquitay
Research Department, Instituto Universitario Hospital Italiano de Buenos Aires, Buenos Aires, Argentina
https://orcid.org/0000-0002-2903-6870

Sergio Muñoz
Department of Public Health - CIGES, Faculty of Medicine, Universidad de La Frontera, Temuco, Chile
https://orcid.org/0000-0001-8383-6599

Gerard Urrútia
Iberoamerican Cochrane Centre - Sant Pau Biomedical Research Institute (IIB Sant Pau) - CIBERESP, Barcelona, Spain
https://orcid.org/0000-0002-8850-0960

Eva Madrid
Interdisciplinary Centre for Health Studies (CIESAL), Universidad de Valparaíso, Viña del Mar, Chile
https://orcid.org/0000-0002-8095-5549

Corresponding author: Nicolás Meza
Angamos 655 Edificio R2 Oficina 1107, Reñaca, Viña del Mar, Chile
nicolas.meza@uv.cl

Word count: 3530

For peer review only

Abstract

Introduction: Clinical research broadly aims to influence decision making in order to promote appropriate healthcare. Funding agencies should prioritise research projects according to needed research topics, methodological and cost-effectiveness considerations, and expected social value. In Chile, there is no local diagnosis regarding recent clinical research that might inform prioritisation for future research funding. This research aims to comprehensively identify and classify Chilean health research studies, elaborating evidence gap maps for the most burdensome local conditions.

Methods and analysis: We will search in electronic databases (MEDLINE, Embase, PsycINFO, CINAHL, LILACS, and WoS) and perform hand searches to retrieve, identify, and classify health research studies conducted in Chile or by authors whose affiliations are based in Chile, from 2000 onwards. We will elaborate evidence matrices for the 20 conditions with the highest burden in Chile (according to the Global Burden of Disease 2019) selected from those defined under the General Regime of the Health Guarantees Act. To elaborate the evidence gap maps, we will consider prioritised interventions and core outcome sets. To identify knowledge gaps and estimate redundant research, we will contrast these gap maps with the available international evidence of high or moderate certainty of evidence, for each specific clinical question. For this purpose, we will search systematic reviews using the GRADE approach.

Ethics and dissemination: No ethical approval is required to conduct this project. We will submit our results in both peer-reviewed journals and scientific conferences. We will aim to disseminate our findings through different academic platforms, social media, local press, among others. The final results will be communicated to local funding agencies and government stakeholders.

Keywords: clinical research, evidence map, scoping review, Chile

Strengths and limitations of this study

- We aim to comprehensively search, identify and classify Chilean clinical research studies by conducting a broad electronic and hand search strategy.
- We will generate and populate evidence gap maps for the 20 most burdensome conditions in the Chilean General Regime of the Health Guarantees Act.
- We will limit the evidence gap maps only to health interventions in prioritised outcomes.
- By focusing on the 20 conditions with the highest burden in Chile, we will not address the whole extent of the locally-prioritised health conditions.
- This project will guide local stakeholders and funding agencies on the identification of current knowledge gaps and redundant research, and the establishment of future research agendas.

Introduction

For peer review only

Clinical research comprehends any investigation addressing questions about prevention, aetiology, diagnosis, treatment and prognosis of any health condition or disease. Primary clinical research and synthesised evidence are the basis for knowledge translation, decision support and implementation in clinical settings, which should ultimately guide the generation of primary research [1,2].

Good quality clinical research, which must be set on priority questions [3,4], is essential for elucidating the optimal and safest approaches to solve health challenges [5]. Clinical research has to be always aimed at informing decision making to make healthcare more effective, less harmful, and less expensive [6]. However, knowledge generation itself faces certain limitations. Much concern exists about poor design and deficient conduction and reporting of clinical research [7], not only because of methodological issues or its effects on scientific advancement but by its practical implications in health decision making, at either individual or population level [8]. Glasziou and Chalmers [8] estimated that 85% of the investments for clinical research end up being avoidable research waste, which may be derived from any step along the research process, by choosing research questions with low-priority, or assessing futile outcomes [3]; conducting research whose rationale of design and methods are inadequate [9]; dismissing the full dissemination of data, causing selective report bias [10]; making research findings unusable because of inadequate reporting (i.e., scientific writing and publishing issues) [11]; and disregarding regulation, governance, and management over clinical research development [12].

Governments, policy-makers, funders and researchers should have accurate and up-to-date pictures of health evidence so that funds and resources are used in the best possible way, which is attuned to the Strategy on Health Research released by the World Health Organization in 2012 [13]. Nevertheless, the use of rigorous clinical research evidence for both clinical practice and health policymaking is still limited [14–16]. The limited budgets for both clinical practice and research make prioritisation a mandatory step for funding agencies, especially governmental organisations. This prioritisation should be done mainly considering the needed research topics, methodological design, cost-effectiveness of the projects, and expected social value [17,18].

Bases of health scientific advances and funding allocation should tend towards (and be guided by) population needs and local decision-making requirements [17,18]. Although some frameworks have been proposed to evaluate research applications [19], the focus on academic background and scientific productivity during the project assessment seem to be predominant [20]. In practice, organisations summon expert panels to judge according to the aforementioned technical and academic merit measures, with heterogeneous considerations about cost, research gaps, social value or local impact[20].

The lack of a prioritised agenda in developing countries may generate a source of inequity, by letting third-party interests (i.e. global research funding, or the pharmaceutical industry from high-income countries) inhabit regional research planning [21–23], in a context of poor governance in this matter [12,24]. The strategic planning of state funding allocation in most countries can still improve the identification of local evidence gaps to be filled by new research, avoiding (and not funding) redundant research. In Chile, clinical research is funded through different government grants, in addition to other types of funding (private agencies, competitive funding, pharmaceutical or medical device industry sponsoring, international grants, etc.) [25], but there is no comprehensive local diagnosis of recent clinical research that could inform prioritisation for future research funding.

This is a protocol for a government-funded project with the following objectives:

1. To identify and classify health research studies conducted in Chile or by authors whose affiliations are based in Chile.
2. To elaborate evidence maps for relevant health conditions in Chile, considering prioritised interventions and core outcome sets.
3. To populate the maps with the primary and secondary evidence and their risk of bias and to identify knowledge gaps and redundant research incorporating international evidence.

Methods and analysis

This section describes the methods for each of the objectives.

Objective 1: Identification and classification of Chilean clinical research

We will conduct a scoping review that will be reported following the Preferred Reporting Items for Systematic Reviews and Meta-Analyses extension for scoping reviews (PRISMA-ScR) [26]. For this protocol, we followed the guidance from the PRISMA-P extension for protocols [27].

Eligibility criteria:

We will consider any clinical study design, either conducted in Chile or at least one affiliation based in Chile. We define clinical studies as those focused on a clinical health topic describing, measuring or exploring a health-related outcome in humans. Considering that our focus in the following objectives will be the highest priority conditions of the last Chilean health reform in which the explicitly guaranteed health conditions were defined, we will consider studies published from 2000 onwards [28].

We will consider any descriptive, observational or experimental primary study design, including case reports, case series, cross-sectional studies, case-control studies, cohort studies, quasi-experimental studies, diagnostic or prognostic studies, mixed-methods studies, and clinical trials. We will also include different formats of evidence synthesis, including systematic reviews, scoping reviews, evidence maps, and overviews [29]. We will not include narrative reviews, editorials, correspondence, letters to the editor, opinion articles, conference proceedings, study protocols and reports without peer review (e.g. preprints). We will also exclude studies whose observation units are biological samples, economic evaluations, modelling studies, validation of instruments, and ecological studies.

Search methods

Electronic search strategy

We will perform a comprehensive search in electronic databases. The search strategies will be designed to retrieve any clinical study, whether conducted in Chile or with at least one affiliation based in Chile, that has been published since 2000 to date without the restriction of language or publication status.

For peer review only

We will search the following databases to be used:

1. MEDLINE vía Ovid SP
2. Embase vía Elsevier
3. PsycINFO via ProQuest SP
4. Cumulative Index to Nursing and Allied Health Literature (CINAHL) via EBSCOhost
5. Latin American and Caribbean Literature in Health Sciences (LILACS)
6. Web of Science (WoS) via Clarivate.

Supplemental **Appendix A** provides details of the electronic search strategy for MEDLINE via Ovid. For our MEDLINE search, we added a highly sensitive filter to identify randomised trials developed by the Cochrane Collaboration [30] and a validated search filter to retrieve systematic reviews developed by the Scottish Intercollegiate Guidelines Network (SIGN) [31]. The strategy proposed was peer-reviewed by another information specialist prior to implementation using the PRESS checklist [32].

Hand search strategy

Considering that research in the Latin American Region might not be adequately indexed in the aforementioned databases, we will conduct complimentary hand searches in the following information sources with the same criteria reported in the electronic search:
We identified seven information resources considered relevant to identify all the evidence that has not been indexed in the databases described in the electronic search and additional four information resources necessary to retrieve trials, reviews, protocols or other types of evidence that contain the inclusion criteria.
1) ANID repository (http://repositorio.conicyt.cl/). Repository of the National Agency for Research and Development that contains research and innovation projects financed by this agency.
2) SciELO (https://scielo.org/es/). Scielo contains the full texts of Chilean Open Access scientific journals, from all areas of knowledge, including peer-reviewed scientific research.
3) Bibliographic catalogues of the Chilean universities and DIBAM (http://www.bncatalogo.gob.cl). This is a unified catalogue of all public institutions in Chile (public libraries, archives and museums) that includes bibliographic information on all national print journals.
4) Bibliographic and National Repository of the Ministry of Health of Chile (http://www.repositoriodigital.minsal.cl/). It contains all the research developed by the Ministry, including clinical practice guidelines, primary studies, population statistics, and other documents.
5) Directory of Open Access Journals (https://doaj.org/). Independent index containing peer-reviewed open access journals.
6) Latin American Repositories Network (http://repositorioslatinoamericanos.uchile.cl). Provides access to full-text electronic publications located in different repositories in Latin American countries.
7) Portal of academic journals of the Universidad de Chile (https://revistas.uchile.cl/); Chilean Academic Journals provides open access to more than 300 publications published by universities, scientific societies, government agencies and Non-Government Organisations.
8) Google Scholar (https://scholar.google.com/)
9) PROSPERO (https://www.crd.york.ac.uk/prospero/).

For peer review only

10) World Health Organization International Clinical Trials Registry Platform (ICTRP, www.who.int/trialsearch/).
11) ClinicalTrials.gov (http://clinicaltrials.gov)
We will follow the same inclusion criteria for studies identified in electronic searches.

Study selection

Two reviewers will independently perform a title and abstract screening. A third reviewer will solve any discrepancies. We will retrieve the full text of each study applying our selection criteria to determine its final inclusion. Afterwards, two reviewers will screen references by full text, solving discrepancies by a third reviewer. For this process, we will use the Covidence platform [33]. We will present a PRISMA 2020 flow diagram showing the process of study selection [34].

Data extraction

Two reviewers will extract the following data from the included studies:

- Bibliographic data: full citation including the list of authors, journal, and date of publication.
- Type of evidence: Primary studies or secondary studies.
- Study design: Case reports, case series, cross-sectional studies, case-control studies, cohorts, quasi-experimental studies, diagnostic studies, prognostic studies, randomised trials, systematic reviews and other forms of synthesised evidence.
- Area of study: We will characterise the retrieved articles by discipline and area of study used by the Organisation for Economic Co-operation and Development (OECD) in their category scheme [35].
- Location of the study: Chile, other country, multicentric study.
- Authors and authorships: Affiliation (based in Chilean institution or not), gender and type of authorship (main author, last author, corresponding author, working groups authorships, among others).
- Diseases or health conditions addressed by each study according to the taxonomy developed by the Global Burden Disease [36].
- Funding: We will classify the type of funding of each article, public or private, competitive funding, pharmaceutical or medical device industry sponsoring, or international grants.
- Conflicts of interest: We will extract the authors' statements from each report, considering descriptions for each author if available.

We will enter the data into a data extraction form (based in Google Sheets, Google LLC)

Summary of data

We do not intend to perform a risk of bias assessment at this stage (see **Objective 3**). We will summarise the findings of each category (i.e. proportion of clinical trials, proportion of studies for each condition). Moreover we will illustrate the trends for each study design and disease category.

For peer review only

Objective 2: Elaboration of evidence matrices for relevant health conditions in Chile, considering prioritised interventions and core outcome sets.

We will elaborate evidence matrices for prioritised health conditions selected from those defined under the General Regime of the Health Guarantees Act (*Garantías Explícitas en Salud,* GES) that was stated in 2004 in Chile. This Regime commanded public and private health providers to guarantee access, opportunity, quality and financial protection for the most relevant programs, diseases or conditions [37]. This Regime contemplates 85 programs, diseases and conditions that have been selected considering the health situation of the population, the effectiveness of the interventions, their contribution to the extension or quality of life and, when possible, their cost effectiveness [38].

We extracted the list of health conditions and excluded those defined as programs (e.g. «Orthosis (or technical help) for people aged 65 years and older» which aims to improve independence in the elderly). These conditions were initially prioritised by the Chilean government based on a local study of the global burden of disease in 2007 [39]. In consultation with the Health Ministry, we decided to prioritise the conditions based on the 2019 report by the Global Burden of Disease (GBD) initiative as it is a more up-to-date resource for decision-making [40]. GBD is a consortium of more than 3,600 researchers who collect, analyse data and provide a tool to visualise the burden and health loss of people due to hundreds of health conditions. The available data is classified by country, incorporating the prevalence, risk factors and the relative harm that each condition causes [40]. We filtered the GBD database by year (2019), location (Chile), context (cause), measure (DALYs, Disability-Adjusted Life Years), and metric (number). We distinguished causes by age groups, and used data from all causes and both sexes. We excluded GES conditions that did not match precisely enough with a GBD cause, according to the consensus of the authors. We provide the details and judgements regarding the reasons for exclusions for each GES problem in **Appendix B**. Then, we selected the 20 conditions with the highest burden in Chile, among those included in GES. This selection closely matched the local study on burden of disease conducted in 2007 which informed decision-making during the constitution of GES [39]. See **Table 1** for the list of the top 20 conditions identified in this process.

Table 1. Prioritised conditions from the General Regime of the Health Guarantees Act (*Garantías Explícitas en Salud*, GES) according to the Global Burden of Disease (GBD) 2019 in Chile, in decreasing order by Disability-Adjusted Life Years (DALYs).

GES condition	Matching GBD cause (age group)	GBD 2019 DALYs, number (CI95%)
Myocardial infarction	Ischemic heart disease (all ages)	214819.57 (227068.32 to 0)
Type 2 diabetes	Diabetes mellitus type 2 (all ages)	170569.88 (213283.00 to 0.19)

Depression in people aged 15 years and over	Depressive disorders (20 plus)	121414.38 (169061.49 to 2.23E-06)
Chronic kidney disease stage 4 and 6	Chronic kidney disease (all ages)	101733.72 (110880.12 to 208.53)
Ischemic stroke in people aged 15 years and over	Ischemic stroke (20 plus)	100161.76 (109460.56 to 94.57)
Stomach cancer	Stomach cancer (all ages)	85929.07 (91647.46 to 1.26)
Lung cancer	Tracheal, bronchus, and lung cancer (all ages)	83674.49 (88943.91 to 0.0002)
Chronic obstructive pulmonary disease (outpatient management)	Chronic obstructive pulmonary disease (all ages)	83190.76 (96438.34 to 0.0002)
Alzheimer's disease and other dementias	Alzheimer's disease and other dementias (all ages)	72825.97 (154958.48 to 4.11)
Colorectal cancer in people aged 15 years and over	Colon and rectum cancer (20 plus)	70756.65 (75584.77 to 1.66)
Hip and/or knee osteoarthritis, mild or moderate, in people aged 55 years and over (medical management)	Osteoarthritis (55 plus)	55576.88 (112169.90 to 1.85)
Prevention of preterm birth	Neonatal preterm birth (all ages)	51479.73 (63435.89 to 0.16)
Chronic hepatitis C	Cirrhosis and other chronic liver diseases due to hepatitis C (all ages)	50969.21 (65323.67 to 0.0005)
Breast cancer in people aged 15 years and over	Breast cancer (20 plus)	47006.54 (51554.51 to 16.13)
Prostate cancer in people aged 15 years and over	Prostate cancer (20 plus)	45854.87 (54664.48 to 2.60)
Schizophrenia	Schizophrenia (all ages)	42501.32 (56297.82 to 0.24)
Bipolar disorder in people aged 15 years and over	Bipolar disorder (20 plus)	38096.12 (58487.76 to 8.43E-06)

For peer review only

Community-acquired pneumonia in people aged 65 years and over (outpatient management)	Lower respiratory infections (65 to 89 years)	34844.45 (38946.70 to 1.10)
Asthma in people aged 15 years and over	Asthma (20 plus)	32694.24 (45713.86 to 0.12)
Secondary subarachnoid haemorrhage to rupture of brain aneurysms	Subarachnoid haemorrhage (all ages)	28635.44 (31612.39 to 1089.41)

We will then conduct the following steps to complete the matrices that will be populated with evidence in the following objective.

Step 1: Identifying the main outcomes

The definition of main outcomes for each health condition is a complex and difficult task, considering that reported outcomes are prone to bias, and that many studies tend to report outcomes with positive or statistically significant findings [41].

For that reason, we will consider agreed standardised sets of outcomes, known as Core Outcome Sets (COS) for identifying the main outcomes. The COS represents a non-restrictive minimum set of outcomes to be assessed and reported in studies for every health condition, and they are permanently updated and revised by the COMET initiative (http://www.comet-initiative.org/). The COS includes outcomes that are most relevant to clinicians, decision makers, patients and carers. If COS are not available for an individual condition, we will build a set of outcomes based on consensus with the input of multidisciplinary experts and patients, considering the existing outcomes embedded in current GES clinical practice guidelines.

Step 2: Identifying the interventions

We will define the interventions for the rows of the matrix for each health condition considering:

- Local Clinical Practice Guidelines: The Chilean Health Ministry developed clinical practice guidelines comprehending primary, secondary and tertiary prevention and treatment for the main health conditions that are guaranteed. We will extract the main interventions from the clinical recommendations in existing guidelines.
- Those prioritised by Cochrane Review Groups and Networks: We will contact Cochrane Groups and Networks' authors in order to gather a list of priority interventions for each health condition, according to their consideration.
- Those identified by the regulatory agency: We will review the authorisations of the Institute of Public Health in Chile as the main regulatory agency in charge of the permits for clinical trials, in order to classify the authorised pharmacological interventions for each health condition.

Step 3: Building up the matrices

For peer review only

We will create evidence gap maps for each included condition. These evidence gap maps will be framed upon grids or matrices that will consider the relevant interventions (defined in **Step 3**) in the rows, and the main outcomes (defined in **Step 2**) in the columns. These grids will be populated in each intersection with the included studies, as we detail in Objective 3 (see **Objective 3** below). We will use evimappr [42], an R [43] package for producing bubble plots, which provides an interactive display for visualising the evidence gap maps.

Objective 3: To populate the maps with local primary and secondary evidence and their risk of bias and to identify knowledge gaps and redundant research incorporating international evidence.

After the completion of the tasks proposed in objective 1, we will select all the studies that might provide evidence for decision making regarding interventions, to populate each of the matrices elaborated as described in objective 2. Whenever an evidence matrix is populated with studies for a certain outcome and intervention, we will refer to it as an evidence map.

Step 1: Identifying the main study designs

From the complete set of research articles included in objective 1, we will separate all those studies with a design relevant for decision making regarding interventions:

1. Randomised controlled trials.
2. Non-randomised primary studies, including controlled clinical trials, quasi-experimental designs (e.g., interrupted time series, controlled before-after studies) and observational studies (cohort studies, case-control studies, case series, analytical cross-sectional studies).
3. Synthesised evidence relevant to interventions (systematic reviews, overviews).

Descriptive studies, qualitative or mixed-methods studies, diagnostic accuracy studies, narrative reviews, scoping reviews and evidence maps will not be considered for this objective as they do not provide evidence supporting the efficacy or effectiveness of interventions.

Step 2: Risk of bias assessment

To assess risk of bias of the included studies, we will use standardised tools for each methodological design: 1) Cochrane Risk of Bias tool for randomised clinical trials [44], 2) ROBINS-I for non-randomised primary studies [45], and 3) ROBIS for systematic reviews [46], We will skip this step in the case of overviews, since currently there is no validated tool for assessing risk of bias in this specific methodological design.

Step 3: Populating evidence gap maps

We will populate each node of the previously elaborated grids or matrices with the selected studies, considering the intervention and outcome on that node. We will represent the populated nodes with visual symbols (bubbles), with different colours according to the type of study and risk of bias, and different sizes according to the number of studies available. Once a matrix is populated with Chilean studies, we will refer to it as a 'local evidence map'.

Step 4: Developing global certainty of the evidence gap maps

We will develop a second map for each prioritised condition populated with rigorous international research (defined as the availability of high or moderate certainty of evidence, for each specific clinical question, according to the GRADE approach [47], that we will refer to as a 'global evidence map'. For this purpose we will conduct a new search aimed at identifying systematic reviews using the GRADE approach, which is currently used for developing Chilean clinical practice guidelines. We will use appropriate filters for the identification of the abovementioned reviews [32]. We will populate these maps with the degree of certainty of evidence, so each node will be classified as "High certainty" or "Moderate certainty" (reflecting that mostly there is enough evidence); and "Low certainty", "Very low certainty", or "No evidence" (reflecting that more evidence is needed).

Step 5: Contrasting global certainty of the evidence maps with local maps

Once step 4 is concluded, we will have two maps for each condition: 1) A local evidence map (populated with local Chilean research), and 2) a global certainty of the evidence map (populated with certainties of evidence from international synthesised evidence). In order to identify knowledge gaps and estimate redundant research, we will contrast these two maps. Once we cross these two maps, we will classify each populated node of the local map as "adequate research" or "redundant research", while each empty node will be classified as "evidence gap", according to the criteria defined in **Table 2**.

Table 2. Possible scenarios in the evidence gap maps.

	**Global evidence map: very low to low certainty of the evidence (or no evidence)**	**Global evidence map: moderate to high certainty of the evidence**
**Local evidence map: No local evidence**	True evidence gap	False evidence gap (unless local conditions require context-specific research)
**Local evidence map: Available local evidence**	Adequate research	Adequate research (if local evidence was published before the review)<br><br>Redundant research(if local evidence was published after the review)

We will consider the absence of local evidence as a gap. If this gap is established in the context of low or very low certainty of evidence in the global map, or if there is no global evidence, we will consider it as a true gap. If the gap is established in the context of high or moderate certainty of evidence in the global gap, we will consider it in general as a false evidence gap.

For peer review only - http://bmjopen.bmj.com/site/about/guidelines.xhtml

If there is local research in the context of a low, very low certainty, or no evidence in the global map, we will consider the local research as adequate. If there is local research in the context of a high or moderate certainty of evidence in the global map, we will consider the research as adequate if it was published before the review that informed the certainty of evidence, or as redundant research if it was published after this review.

Patient and public involvement

No patients were involved in the development of this protocol.

Ethics and dissemination

No ethical approval is required to conduct this project. Our findings will be submitted to peer-reviewed journals, and scientific conferences. To amplify the impact of our work and to accomplish knowledge translation objectives, we will disseminate the results through different platforms, including academic social media, blogs, local press, among others. At the same time, we will aim to communicate the final results to local funding agencies and government stakeholders, in order to inform the elaboration of future national research agendas.

Acknowledgments: We thank Ivan Solà from the Iberoamerican Cochrane Centre - Sant Pau Biomedical Research Institute (IIB Sant Pau) - CIBERESP (Barcelona, Spain) for his valuable support in drafting and refining our search strategy.

Authors' contributions: The study concept was developed by JP-B, NM, JVAF and EM. The manuscript of the protocol was drafted by JP-B, NM, JVAF, CME-L and EM. SM and GU developed and provided feedback for all sections of the review protocol and approved the final manuscript. The search strategy was developed by CME-L and JP-B. Study selection, data extraction, quality assessment and gap maps elaboration will be performed by all the authors. All authors reviewed and approved the final version of the manuscript.
Funding: FONDECYT Grant 1212037 from the Chilean National Agency of Research and Development (ANID). The funding agency had no involvement in the conception, development, drafting or approval of this manuscript.
Competing interests: All the authors have conducted clinical research in Chile or associated with Chilean researchers or institutions. Nevertheless, decisions about inclusion, data extraction and quality assessment of these studies, will be conducted by independent researchers within our team. We declare no other conflict of interests.
Patient consent for publication: Not required.
Provenance and peer review: Not commissioned; externally peer-reviewed.
Data sharing statements: No additional data are available.

References

1 Vandvik PO, Brandt L, Alonso-Coello P, *et al.* Creating Clinical Practice Guidelines We Can Trust, Use, and Share. *Chest* 2013;**144**:381–9. doi:10.1378/chest.13-0746

For peer review only

2 Vandvik PO, Brandt L. Future of Evidence Ecosystem Series: Evidence ecosystems and learning health systems: why bother? *Journal of Clinical Epidemiology* 2020;**123**:166–70. doi:10.1016/j.jclinepi.2020.02.008

3 Chalmers I, Bracken MB, Djulbegovic B, *et al. How to increase value and reduce waste when research priorities are set*. Elsevier 2014. doi:10.1016/S0140-6736(13)62229-1

4 Dechartres A, Ravaud P. *Better prioritization to increase research value and decrease waste*. BioMed Central 2015. doi:10.1186/s12916-015-0492-3

5 Cartabellotta A, Tilson JK. The ecosystem of evidence cannot thrive without efficiency of knowledge generation, synthesis, and translation. *Journal of Clinical Epidemiology* 2019;**110**:90–5. doi:10.1016/j.jclinepi.2019.01.008

6 World Health Organization. *The WHO strategy on research for health*. World Health Organization 2012.

7 Ioannidis JPA, Greenland S, Hlatky MA, *et al. Increasing value and reducing waste in research design, conduct, and analysis*. Elsevier 2014. doi:10.1016/S0140-6736(13)62227-8

8 Glasziou P, Chalmers I. Research waste is still a scandal- An essay by Paul Glasziou and Iain Chalmers. *The BMJ* 2018;**363**:10–2. doi:10.1136/bmj.k4645

9 Ioannidis JPA, Greenland S, Hlatky MA, *et al. Increasing value and reducing waste in research design, conduct, and analysis*. Elsevier 2014. doi:10.1016/S0140-6736(13)62227-8

10 Chan A-W, Song F, Vickers A, *et al.* Increasing value and reducing waste: addressing inaccessible research. *The Lancet* 2014;**383**:257–66. doi:10.1016/S0140-6736(13)62296-5

11 Glasziou P, Altman DG, Bossuyt P, *et al.* Reducing waste from incomplete or unusable reports of biomedical research. *The Lancet* 2014;**383**:267–76. doi:10.1016/S0140-6736(13)62228-X

12 Salman RA-S, Beller E, Kagan J, *et al.* Increasing value and reducing waste in biomedical research regulation and management. *The Lancet* 2014;**383**:176–85. doi:10.1016/S0140-6736(13)62297-7

13 World Health Organization. Research and development to meet health needs in developing countries: strengthening global financing and coordination: report of the consultative expert working group on research and development: financing and coordination. 2012.

14 Brownson RC, Fielding JE, Maylahn CM. Evidence-Based Public Health: A Fundamental Concept for Public Health Practice. *Annu Rev Public Health* 2009;**30**:175–201. doi:10.1146/annurev.publhealth.031308.100134

15 Lester L, Haby MM, Chapman E, *et al.* Evaluation of the performance and achievements of the WHO Evidence-informed Policy Network (EVIPNet) Europe. In Review 2020. doi:10.21203/rs.2.21663/v1

16 van de Goor I, Hämäläinen R-M, Syed A, *et al.* Determinants of evidence use in public health policy making: Results from a study across six EU countries. *Health Policy* 2017;**121**:273–81. doi:10.1016/j.healthpol.2017.01.003

17 Ciarli T, Ràfols I. The relation between research priorities and societal demands: The case of rice. *Research Policy* 2019;**48**:949–67. doi:10.1016/j.respol.2018.10.027

18 Mutapi F. Africa should set its own health-research agenda. *Nature* 2019;**575**:567. doi:10.1038/d41586-019-03627-9

19 Tuffaha HW, Aitken J, Chambers S, *et al.* A Framework to Prioritise Health Research Proposals for Funding: Integrating Value for Money. *Appl Health Econ Health Policy* 2019;**17**:761–70. doi:10.1007/s40258-019-00495-2

For peer review only

20 Cassi L, Lahatte A, Rafols I, *et al.* Improving fitness: Mapping research priorities against societal needs on obesity. *Journal of Informetrics* 2017;**11**:1095–113. doi:10.1016/j.joi.2017.09.010
21 Aguilera B. Trends in clinical trials performed in Chile. *Rev méd Chile* 2021;**149**:110–8. doi:10.4067/S0034-98872021000100110
22 Atal I, Trinquart L, Porcher R, *et al.* Differential Globalization of Industry- and Non-Industry–Sponsored Clinical Trials. *PLoS ONE* 2015;**10**:e0145122. doi:10.1371/journal.pone.0145122
23 Hoffman SJ, Gunn E, Rogers Van Katwyk S, *et al.* Systematic analysis of global health research funding in Canada, 2000–2016. *Can J Public Health* 2020;**111**:80–95. doi:10.17269/s41997-019-00247-8
24 Smits P, Champagne F. Governance of health research funding institutions: an integrated conceptual framework and actionable functions of governance. *Health Res Policy Sys* 2020;**18**:22. doi:10.1186/s12961-020-0525-z
25 Agencia Nacional de Investigación y Desarrollo, Ministerio de Ciencia, Tecnología, Conocimiento e Innovación, Chile. ANID, Concursos. 2021.https://www.anid.cl/concursos/ (accessed 2 Jul 2021).
26 Tricco AC, Lillie E, Zarin W, *et al.* PRISMA Extension for Scoping Reviews (PRISMA-ScR): Checklist and Explanation. *Ann Intern Med* 2018;**169**:467–73. doi:10.7326/M18-0850
27 Moher D, Shamseer L, Clarke M, *et al.* Preferred reporting items for systematic review and meta-analysis protocols (PRISMA-P) 2015 statement. *Syst Rev* 2015;**4**:1. doi:10.1186/2046-4053-4-1
28 Bastias G, Pantoja T, Leisewitz T, *et al.* Health care reform in Chile. *Canadian Medical Association Journal* 2008;**179**:1289–92. doi:10.1503/cmaj.071843
29 Franco JVA, Arancibia M, Simancas-Racines D, *et al.* Syntheses of biomedical information: narrative reviews, systematic reviews and emerging formats. *Medwave* 2018;**18**:e7354–e7354. doi:10.5867/medwave.2018.07.7354
30 Lefebvre C, Glanville J, Briscoe S, *et al.* Technical Supplement to Chapter 4: Searching for and selecting studies. In: *Cochrane Handbook for Systematic Reviews of Interventions Version 6.2 (updated February 2021)*. Cochrane 2021. www.training.cochrane.org/handbook. (accessed 3 Sep 2021).
31 SIGN. Search filters. Scottish Intercollegiate Guidelines Network (SIGN). https://www.sign.ac.uk/what-we-do/methodology/search-filters/ (accessed 3 Sep 2021).
32 McGowan J, Sampson M, Salzwedel DM, *et al.* PRESS Peer Review of Electronic Search Strategies: 2015 Guideline Statement. *Journal of Clinical Epidemiology* 2016;**75**:40–6. doi:10.1016/j.jclinepi.2016.01.021
33 Veritas Health Innovation. *Covidence*. Melbourne, Australia: www.covidence.org
34 Page MJ, McKenzie JE, Bossuyt PM, *et al.* The PRISMA 2020 statement: an updated guideline for reporting systematic reviews. *BMJ* 2021;:n71. doi:10.1136/bmj.n71
35 OECD Category Scheme. http://help.prod-incites.com/inCites2Live/filterValuesGroup/researchAreaSchema/oecdCategoryScheme.html (accessed 14 Jul 2020).
36 Lozano R, Naghavi M, Foreman K, *et al.* Global and regional mortality from 235 causes of death for 20 age groups in 1990 and 2010: a systematic analysis for the Global Burden of Disease Study 2010. *The Lancet* 2012;**380**:2095–128. doi:10.1016/S0140-6736(12)61728-0
37 Superintendencia de Salud, MINSAL. Ley N° 19.966. Superintendencia de Salud, MINSAL. 2004.https://www.supersalud.gob.cl/normativa/668/w3-article-554.html (accessed 1 Sep 2021).
38 Urriola C, Infante A, Aguilera I, *et al.* La reforma de salud chilena a diez años de su implementación. *Salud Publica de Mexico* 2016;**58**:514–21. doi:10.21149/spm.v58i5.8240

For peer review only

39 Departamento de Epidemiología, MINSAL. Estudio Carga de Enfermedad y Carga atribuible. 2007.http://epi.minsal.cl/estudio-de-carga-de-enfermedades/ (accessed 1 Sep 2021).
40 Vos T, Lim SS, Abbafati C, *et al.* Global burden of 369 diseases and injuries in 204 countries and territories, 1990–2019: a systematic analysis for the Global Burden of Disease Study 2019. *The Lancet* 2020;**396**:1204–22. doi:10.1016/S0140-6736(20)30925-9
41 Dwan K, Altman DG, Arnaiz JA, *et al.* Systematic Review of the Empirical Evidence of Study Publication Bias and Outcome Reporting Bias. *PLoS ONE* 2008;**3**:e3081. doi:10.1371/journal.pone.0003081
42 Haddaway N, Keenan C. *evimappr: an R package for creating bubble plots for evidence maps.* 2021. https://github.com/nealhaddaway/evimappr/
43 R Core Team. *R: A language and environment for statistical computing*. 2021. https://www.R-project.org
44 Higgins JPT, Altman DG, Gotzsche PC, *et al.* The Cochrane Collaboration's tool for assessing risk of bias in randomised trials. *BMJ* 2011;**343**:d5928–d5928. doi:10.1136/bmj.d5928
45 Sterne JAC, Savović J, Page MJ, *et al.* RoB 2: a revised tool for assessing risk of bias in randomised trials. *BMJ* 2019;:l4898. doi:10.1136/bmj.l4898
46 Whiting P, Savović J, Higgins JPT, *et al.* ROBIS: A new tool to assess risk of bias in systematic reviews was developed. *Journal of Clinical Epidemiology* 2016;**69**:225–34. doi:10.1016/j.jclinepi.2015.06.005
47 Guyatt G, Oxman AD, Akl EA, *et al.* GRADE guidelines: 1. Introduction—GRADE evidence profiles and summary of findings tables. *Journal of Clinical Epidemiology* 2011;**64**:383–94. doi:10.1016/j.jclinepi.2010.04.026

For peer review only

Appendix A. Search strategy for a scoping review protocol for trends in Chilean clinical research

MEDLINE Ovid search strategy

Ovid MEDLINE(R) and Epub Ahead of Print, In-Process, In-Data-Review & Other Non-Indexed Citations, Daily and Versions(R) <1946 to June 17, 2021>

#1	Chile.ia,in.
#2	exp Chile/
#3	((Hospital or Clinic* or funda* or universi*) adj3 (Arica or Tarapaca or Antofagasta or Atacama or Coquimbo or Valparaiso or "Metropolitana de Santiago" or O Higgins or Maule or nuble or Biobio or Araucania or Los Rios or Los Lagos or Aysen or Magallanes)).ia,in.
#4	1 or 2 or 3
#5	randomized controlled trial.pt.
#6	controlled clinical trial.pt.
#7	randomized.ab.
#8	placebo.ab.
#9	drug therapy.fs.
#10	randomly.ab.
#11	trial.ab.
#12	groups.ab.
#13	5 or 6 or 7 or 8 or 9 or 10 or 11 or 12
#14	exp animals/ not humans.sh.
#15	13 not 14
#16	4 and 15
#17	Meta-Analysis as Topic/
#18	meta analy$.tw.
#19	metaanaly$.tw.
#20	Meta-Analysis/

For peer review only

#21	(systematic adj (review$1 or overview$1)).tw.
#22	exp Review Literature as Topic/
#23	17 or 18 or 19 or 20 or 21 or 22
#24	cochrane.ab.
#25	embase.ab.
#26	(psychlit or psyclit).ab.
#27	(psychinfo or psycinfo).ab.
#28	(cinahl or cinhal).ab.
#29	science citation index.ab.
#30	bids.ab.
#31	cancerlit.ab.
#32	24 or 25 or 16 or 27 or 28 or 29 or 30 or 31
#33	reference list$.ab.
#34	bibliograph$.ab.
#35	hand-search$.ab.
#36	relevant journals.ab.
#37	manual search$.ab.
#38	33 or 34 or 35 or 36 or 37
#39	selection criteria.ab.
#40	data extraction.ab.
#41	39 or 40
#42	Review/
#43	41 and 42
#44	Comment/
#45	Letter/
#46	Editorial/
#47	animal/

For peer review only

#48	human/
#49	47 not (47 and 48)
#50	44 or 45 or 46 or 49
#51	23 or 32 or 38 or 43 or 50
#52	4 and 51
#53	16 or 52
#54	limit 53 to yr=2000-2021

#1	exp cohort studies/
#2	cohort$.tw.
#3	exp Observational Study/
#4	observational.ab,ti.
#5	exp Cross-Sectional Studies/
#6	Prevalence Stud*.ti,ab.
#7	Cross Sectional*.ti,ab.
#8	descriptive.mp.
#9	Case.ti,ab. AND (report*.ti,ab. OR case serie*.ti,ab.)
#10	exp Diagnosis/
#11	Diagnos.ti,ab
#12	1 or 2 or 3 or 4 or 5 or 6 or 7 or 8 or 9 or 10 or 11 or 12
#13	Chile.ia,in.
#14	exp Chile/
#15	((Hospital or Clinic* or funda* or universi*) adj3 (Arica or Tarapaca or Antofagasta or Atacama or Coquimbo or Valparaiso or "Metropolitana de Santiago" or O Higgins or Maule or nuble or Biobio or Araucania or Los Rios or Los Lagos or Aysen or Magallanes)).ia,in.
#16	13 or 14 or 15
#17	12 and 16
#18	limit 17 to yr=2000-2021

For peer review only

Appendix B. Potentially eligible and excluded conditions/programs from the General Regime of the Health Guarantees Act (*Garantías Explícitas en Salud*, GES).

Appendix B.1. Potentially eligible conditions from GES, in descending order according to Global Burden of Disease 2019 (GBD 2019) Disability-Adjusted Life Years (DALYs).

GES condition	Matching GBD 2019 cause (age groups)	DALYs, number (CI%95)
Myocardial infarction	Ischemic heart disease (all ages)	214819.571800329 (227068.320701352 to 0)
Type 2 diabetes	Diabetes mellitus type 2 (all ages)	170569.880724338 (213283.001985072 to 0.192722088496509)
Depression in people aged 15 years and over	Depressive disorders (20 plus)	121414.377867517 (169061.491027657 to 2.2253601218192E-06)
Chronic kidney disease stage 4 and 6	Chronic kidney disease (all ages)	101733.722907926 (110880.123710307 to 208.525669506703)
Ischemic stroke in people aged 15 years and over	Ischemic stroke (20 plus)	100161.760973747 (109460.558336013 to 94.57213560342)
Stomach cancer	Stomach cancer (all ages)	85929.065773134 (91647.4644054603 to 1.26268744062835)

For peer review only

Lung cancer	Tracheal, bronchus, and lung cancer (all ages)	83674.4888793253 (88943.9075435297 to 0.000258752006588802)
Chronic obstructive pulmonary disease (outpatient management)	Chronic obstructive pulmonary disease (all ages)	83190.7630904602 (96438.3426616626 to 0.000164947566358871)
Alzheimer's disease and other dementias	Alzheimer's disease and other dementias (all ages)	72825.9715672292 (154958.475973897 to 4.10904084133898)
Colorectal cancer in people aged 15 years and over	Colon and rectum cancer (20 plus)	70756.6453332632 (75584.7656820732 to 1.65874626595222)
Operable congenital heart defect in people younger than 15 years	Congenital birth defects (0 to 14)	60558.1526518899 (79184.2191913363 to 3.88473612239553E-06)
Hip and/or knee osteoarthritis, mild or moderate, in people aged 55 years and over (medical management)	Osteoarthritis (55 plus)	55576.8772705614 (112169.901045112 to 1.84635512150662)
Prevention of preterm birth	Neonatal preterm birth (all ages)	51479.7300991547 (63435.89308863 to 0.158666035639263)
Chronic hepatitis C	Cirrhosis and other chronic liver diseases due to hepatitis C (all ages)	50969.2141174526 (65323.6655151894 to 0.000549020300847728)
Breast cancer in people aged 15 years and over	Breast cancer (20 plus)	47006.5369289545 (51554.507927785 to 16.1265082764972)
Prostate cancer in people aged 15 years and over	Prostate cancer (20 plus)	45854.8719687154 (54664.4754416884 to 2.59747447089968)

For peer review only

Schizophrenia	Schizophrenia (all ages)	42501.3194923616 (56297.8169798092 to 0.240749074074181)
Bipolar disorder in people aged 15 years and over	Bipolar disorder (20 plus)	38096.1151342666 (58487.7573236035 to 8.43274832801397E-06)
Community-acquired pneumonia in people aged 65 years and over (outpatient management)	Lower respiratory infections (65 to 89 years)	34844.453660895 (38946.6963391689 to 1.10402107319051)
Asthma in people aged 15 years and over	Asthma (20 plus)	32694.2377867036 (45713.8574539779 to 0.116280132000234)
Secondary subarachnoid hemorrhage to rupture of brain aneurysms	Subarachnoid hemorrhage (all ages)	28635.4404153411 (31612.3869284669 to 1089.40824984095)
Human Immunodeficiency Virus and Acquired Immunodeficiency Syndrome (HIV/AIDS)	HIV/AIDS (all ages)	27181.8681774182 (32191.0340641685 to 29.3017079071855)
Cervical cancer	Cervical cancer (all ages)	25200.4297936711 (30005.252368359 to 0.000474071744494449)
Non-refractory epilepsy in people aged 15 years and older	Idiopathic epilepsy (20 plus)	24620.5412700062 (41485.3459737919 to 3501.83306715692)
Kidney cancer in people aged 15 years and older	Kidney cancer (20 plus)	23991.4065239036 (26418.6543665931 to 534.114744605753)
Leukemia in people aged 15 years and older	Leukemia (20 plus)	21325.5560639148 (22901.6129356555 to 0.000813097105958376)

For peer review only

Parkinson’s disease	Parkinson's disease (all ages)	20331.9233118182 (22034.4067968826 to 147.770845873412)
Epithelial ovarian cancer	Ovarian cancer (20 plus)	15600.3173555778 (17963.5312380173 to 1407.16245468356)
Rheumatoid arthritis	Rheumatoid arthritis (all ages)	14399.6554552725 (18512.9913244069 to 0.00362617834828417)
Primary tumors of the central nervous system in people aged 15 years and older	Brain and central nervous system cancer (20 plus)	14245.6107807219 (16062.6268023673 to 133.432208773944)
Multiple myeloma in people aged 15 years and older	Multiple myeloma (20 plus)	13895.9089272371 (15829.801624441 to 9.75441705033119)
Chronic hepatitis due to hepatitis B virus	Cirrhosis and other chronic liver diseases due to hepatitis B (all ages)	13680.7698238002 (19065.5815024126 to 0.0085089965860338)
Bladder cancer in people aged 15 years and older	Bladder cancer (20 plus)	12707.3764274785 (14072.9882127301 to 3054.38153002352)
Cancer in children under the age of 15 years	Neoplasms (0 to 14)	12303.3788104734 (14469.5035669086 to 113.905690992672)
Total hip endoprothesis for people aged 65 years and older and arthrosis of the hip with severe functional limitations	Osteoarthritis hand (65 to 89 years)	10568.6092353729 (21500.8167895118 to 0)
Cataract surgery	Cataract (all ages)	9770.85045117638 (13317.247585107 to 3232.36904442194)

For peer review only

Moderate and acute bronchial asthma in children under the age of 15 years	Asthma (0 to 14)	7963.77834457529 (13656.3525907285 to 51.6514027019469)
Type I diabetes mellitus	Diabetes mellitus type 1 (all ages)	7429.36242517253 (9651.21392599779 to 0)
Testicular cancer in people aged 15 years and older	Testicular cancer (20 plus)	6425.06386606812 (7673.24210673214 to 0)
Refractory epilepsy in children between the ages of 1 and 15 years	Idiopathic epilepsy (0 to 14)	5569.06914520869 (11130.3966112371 to 105.052591863323)
Heavy consumption or low to moderate risk of addiction to alcohol and drugs in people under the age of 20 years	Substance use disorders (<20 years)	5312.55244366324 (7461.50867463031 to 698.314690531639)
Treatment for the eradication of *Helicobacter pylori*	Gastritis and duodenitis (all ages)	3833.44230358697 (5407.26107597058 to 31.0664601228219)
Differentiated and medullary thyroid cancer in people aged 15 years and older	Thyroid cancer (20 plus)	3524.95356842812 (3925.21814487822 to 0)
Treatment for benign prostatic hyperplasia in symptomatic people	Benign prostatic hyperplasia (all ages)	3136.15726142161 (4876.83538193793 to 0.00194715937414714)
Surgical treatment for chronic lesions of the mitral and tricuspid valves in people aged 15 years and older	Non-rheumatic degenerative mitral valve disease (20 plus)	2321.91890136389 (2772.42279203503 to 0.00640083277215629)
Refractive errors in people aged 65 years and older	Refraction disorders (65 to 89 years)	2267.63302256029 (3291.72011313406 to 4699.20120851181)
Relapsing-remitting multiple sclerosis	Multiple sclerosis (all ages)	2192.67301330785 (2860.98207134884 to

For peer review only

		0.000916848432839514)
Cholecystectomy for the prevention of gallbladder cancer in people between the ages of 35 and 49 years	Gallbladder and biliary diseases (25 to 49)	2061.70765625961 (2857.83007158215 to 7.89297979047496)
Outpatient care for Acute Respiratory Infection (ARI) in children under the age of 5 years	Upper respiratory infections (under 5)	1783.98509162834 (2869.84870415435 to 53.7737581195539)
Cleft lip and palate	Orofacial clefts (0 to 14)	66.8835836316003 (109.496663940256 to 17707.9341422313)

For peer review only

Appendix B.2. Excluded GES conditions

GES condition	Reason for exclusion
Primary or essential arterial hypertension in people aged 15 years and older	Matching condition not found in GBD 2019 data
Hemophilia	Matching condition not found in GBD 2019 data
Cystic fibrosis	Matching condition not found in GBD 2019 data
Juvenile idiopathic arthritis	Matching condition not found in GBD 2019 data
Hypothyroidism in people aged 15 years and older	Matching condition not found in GBD 2019 data.
Systemic lupus erythematosis	Matching condition not found in GBD 2019 data
Bilateral hearing loss in people aged 65 years and older requiring hearing aids	Matching condition not found in GBD 2019 data
Diabetic retinopathy	Matching condition not found in GBD 2019 data
Nontraumatic rhegmatogenous retinal detachment	Matching condition not found in GBD 2019 data
Scoliosis surgery for people under the age of 25 years	Matching condition not found in GBD 2019 data
Osteosarcoma in people aged 15 years and older	Matching condition not found in GBD 2019 data
Strabismus in children under the age of 9 years	Matching condition not found in GBD 2019 data
Hip dysplasia	Matching condition not found in GBD 2019 data
Treatment for moderate, severe and profound hearing loss in children under four	Matching condition not found in GBD 2019 data
Serious polytrauma	GES condition does not match with a unique cause in GBD 2019 data
Lymphomas in people aged 15 years and older	GES condition does not match with a unique cause in GBD 2019 data
Spinal dysraphism	Matching condition not found in GBD 2019 data

For peer review only

Surgical treatment of chronic lesions of the aortic valve in people aged 15 years and older	Matching condition not found in GBD 2019 data
Surgery for herniated nucleus pulposus	Matching condition not found in GBD 2019 data
Severe burns	Matching condition not found in GBD 2019 data
Serious eye trauma	Matching condition not found in GBD 2019 data
Impulse and conduction disorders in people aged 15 years and older who require a pacemaker	Matching condition not found in GBD 2019 data
Respiratory distress syndrome in the newborn	Matching condition not found in GBD 2019 data
Bilateral sensorineural hearing loss in premature babies	Matching condition not found in GBD 2019 data
Bronchopulmonary dysplasia in premature babies	Matching condition not found in GBD 2019 data
Retinopathy in premature babies	Matching condition not found in GBD 2019 data
Moderate and serious traumatic brain injury	Matching condition not found in GBD 2019 data

Appendix B.3. Excluded GES programs

Orthosis (or technical help) for people aged 65 years and older
Pain relief and palliative care for advanced cancer
Analgesia in childbirth
Integral dental health care for girls and boys aged 6 years old
Outpatient treatment for odontological emergencies
Integral dental health care for 60 year old adults
Integral dental health care for pregnant women
Secondary prevention of end-stage chronic kidney disease

For peer review only

PRISMA-P (Preferred Reporting Items for Systematic review and Meta-Analysis Protocols) 2015 checklist: recommended items to address in a systematic review protocol*

Section and topic	Item No	Checklist item	Page
**ADMINISTRATIVE INFORMATION**			
Title:			
Identification	1a	Identify the report as a protocol of a systematic review	1
Update	1b	If the protocol is for an update of a previous systematic review, identify as such	N/A
Registration	2	If registered, provide the name of the registry (such as PROSPERO) and registration number	N/A
Authors:			
Contact	3a	Provide name, institutional affiliation, e-mail address of all protocol authors; provide physical mailing address of corresponding author	2
Contributions	3b	Describe contributions of protocol authors and identify the guarantor of the review	15
Amendments	4	If the protocol represents an amendment of a previously completed or published protocol, identify as such and list changes; otherwise, state plan for documenting important protocol amendments	N/A
Support:			
Sources	5a	Indicate sources of financial or other support for the review	N/A
Sponsor	5b	Provide name for the review funder and/or sponsor	15
Role of sponsor or funder	5c	Describe roles of funder(s), sponsor(s), and/or institution(s), if any, in developing the protocol	15
**INTRODUCTION**			
Rationale	6	Describe the rationale for the review in the context of what is already known	3-4
Objectives	7	Provide an explicit statement of the question(s) the review will address with reference to participants, interventions, comparators, and outcomes (PICO)	5
**METHODS**			
Eligibility criteria	8	Specify the study characteristics (such as PICO, study design, setting, time frame) and report characteristics (such as years considered, language, publication status) to be used as criteria for eligibility for the review	5-6
Information sources	9	Describe all intended information sources (such as electronic databases, contact with study authors, trial registers or other grey literature sources) with planned dates of coverage	6-7
Search strategy	10	Present draft of search strategy to be used for at least one electronic database, including planned limits, such that it could be repeated	Appendix A

For peer review only

Study records:			
Data management	11a	Describe the mechanism(s) that will be used to manage records and data throughout the review	7-8
Selection process	11b	State the process that will be used for selecting studies (such as two independent reviewers) through each phase of the review (that is, screening, eligibility and inclusion in meta-analysis)	7
Data collection process	11c	Describe planned method of extracting data from reports (such as piloting forms, done independently, in duplicate), any processes for obtaining and confirming data from investigators	7-8
Data items	12	List and define all variables for which data will be sought (such as PICO items, funding sources), any pre-planned data assumptions and simplifications	7-8
Outcomes and prioritization	13	List and define all outcomes for which data will be sought, including prioritization of main and additional outcomes, with rationale	11
Risk of bias in individual studies	14	Describe anticipated methods for assessing risk of bias of individual studies, including whether this will be done at the outcome or study level, or both; state how this information will be used in data synthesis	13
Data synthesis	15a	Describe criteria under which study data will be quantitatively synthesised	
	15b	If data are appropriate for quantitative synthesis, describe planned summary measures, methods of handling data and methods of combining data from studies, including any planned exploration of consistency (such as I^2, Kendall's τ)	
	15c	Describe any proposed additional analyses (such as sensitivity or subgroup analyses, meta-regression)	
	15d	If quantitative synthesis is not appropriate, describe the type of summary planned	8, 13, 14
Meta-bias(es)	16	Specify any planned assessment of meta-bias(es) (such as publication bias across studies, selective reporting within studies)	N/A
Confidence in cumulative evidence	17	Describe how the strength of the body of evidence will be assessed (such as GRADE)	13-14

*** It is strongly recommended that this checklist be read in conjunction with the PRISMA-P Explanation and Elaboration (cite when available) for important clarification on the items. Amendments to a review protocol should be tracked and dated. The copyright for PRISMA-P (including checklist) is held by the PRISMA-P Group and is distributed under a Creative Commons Attribution Licence 4.0.**

From: Shamseer L, Moher D, Clarke M, Ghersi D, Liberati A, Petticrew M, Shekelle P, Stewart L, PRISMA-P Group. Preferred reporting items for systematic review and meta-analysis protocols (PRISMA-P) 2015: elaboration and explanation. BMJ. 2015 Jan 2;349(jan02 1):g7647.

For peer review only

BMJ Open

Mapping Chilean clinical research: a protocol for a scoping review and multiple evidence gap maps

Journal:	*BMJ Open*
Manuscript ID	bmjopen-2021-057555.R2
Article Type:	Protocol
Date Submitted by the Author:	07-Apr-2022
Complete List of Authors:	Pérez-Bracchiglione, Javier; Universidad de Valparaíso, Interdisciplinary Centre for Health Studies (CIESAL)<br>Meza, Nicolás; Universidad de Valparaíso<br>Franco, Juan Victor Ariel; Hospital Italiano de Buenos Aires, Instituto Universitario; Instituto Universitario Hospital Italiano<br>Escobar Liquitay, Camila Micaela; Instituto Universitario Hospital Italiano de Buenos Aires, Research Department<br>Munoz, Sergio; Universidad de La Frontera, Public Heath<br>Urrutia, Gerard; Iberoamerican Cochrane Centre<br>Madrid, Eva; Universidad de Valparaiso, Escuela de Medicina
**Primary Subject Heading**:	Epidemiology
Secondary Subject Heading:	Health policy, Medical publishing and peer review, Health services research
Keywords:	PUBLIC HEALTH, STATISTICS & RESEARCH METHODS, HEALTH SERVICES ADMINISTRATION & MANAGEMENT

SCHOLARONE™
Manuscripts

Mapping Chilean clinical research: a protocol for a scoping review and multiple evidence gap maps

Authors:

Javier Bracchiglione
Interdisciplinary Centre for Health Studies (CIESAL), Universidad de Valparaíso, Viña del Mar, Chile
https://orcid.org/0000-0001-8738-2184

Nicolás Meza
Interdisciplinary Centre for Health Studies (CIESAL), Universidad de Valparaíso, Viña del Mar, Chile
https://orcid.org/0000-0001-9505-0358

Juan Victor Ariel Franco
Research Department, Instituto Universitario Hospital Italiano de Buenos Aires, Buenos Aires, Argentina
https://orcid.org/0000-0003-0411-899X

Camila Micaela Escobar-Liquitay
Research Department, Instituto Universitario Hospital Italiano de Buenos Aires, Buenos Aires, Argentina
https://orcid.org/0000-0002-2903-6870

Sergio Muñoz
Department of Public Health - CIGES, Faculty of Medicine, Universidad de La Frontera, Temuco, Chile
https://orcid.org/0000-0001-8383-6599

Gerard Urrútia
Iberoamerican Cochrane Centre - Sant Pau Biomedical Research Institute (IIB Sant Pau) - CIBERESP, Barcelona, Spain
https://orcid.org/0000-0002-8850-0960

Eva Madrid
Interdisciplinary Centre for Health Studies (CIESAL), Universidad de Valparaíso, Viña del Mar, Chile
https://orcid.org/0000-0002-8095-5549

Corresponding author: Nicolás Meza
Angamos 655 Edificio R2 Oficina 1107, Reñaca, Viña del Mar, Chile
nicolas.meza@uv.cl

Word count: 4058

For peer review only

Abstract

Introduction: Clinical research broadly aims to influence decision making in order to promote appropriate healthcare. Funding agencies should prioritise research projects according to needed research topics, methodological and cost-effectiveness considerations, and expected social value. In Chile, there is no local diagnosis regarding recent clinical research that might inform prioritisation for future research funding. This research aims to comprehensively identify and classify Chilean health research studies, elaborating evidence gap maps for the most burdensome local conditions.

Methods and analysis: We will search in electronic databases (MEDLINE, Embase, PsycINFO, CINAHL, LILACS, and WoS) and perform hand searches to retrieve, identify, and classify health research studies conducted in Chile or by authors whose affiliations are based in Chile, from 2000 onwards. We will elaborate evidence matrices for the 20 conditions with the highest burden in Chile (according to the Global Burden of Disease 2019) selected from those defined under the General Regime of the Health Guarantees Act. To elaborate the evidence gap maps, we will consider prioritised interventions and core outcome sets. To identify knowledge gaps and estimate redundant research, we will contrast these gap maps with the available international evidence of high or moderate certainty of evidence, for each specific clinical question. For this purpose, we will search systematic reviews using the GRADE approach.

Ethics and dissemination: No ethical approval is required to conduct this project. We will submit our results in both peer-reviewed journals and scientific conferences. We will aim to disseminate our findings through different academic platforms, social media, local press, among others. The final results will be communicated to local funding agencies and government stakeholders.

Discussion: We aim to provide an accurate and up-to-date picture of the research gaps—to be filled by new future findings—and the identification of redundant research, which will constitute relevant information for local decision-makers.

Keywords: clinical research, evidence map, scoping review, bibliometrics, Chile

Strengths and limitations of this study

- The protocol for this scoping review includes a comprehensive electronic and hand search strategy.
- We will be able to accurately elucidate local evidence gaps by first constructing evidence grids and then populating them with the results of our search.
- Considering that we used the estimations from the Global Burden of Disease 2019 for prioritising the most important diseases in Chile, we might be neglecting some important clinical entities, such as multicomponent diseases.
- We will develop gap maps only for the 20 conditions with the highest burden in Chile.

For peer review only

Introduction

Clinical research comprehends any investigation addressing questions about prevention, aetiology, diagnosis, treatment and prognosis of any health condition or disease. Primary clinical research and synthesised evidence are the basis for knowledge translation, decision support and implementation in clinical settings, which should ultimately guide the generation of primary research [1,2].

Good quality clinical research, which must be set on priority questions [3,4], is essential for elucidating the optimal and safest approaches to solve health challenges [5]. Clinical research has to be always aimed at informing decision making to make healthcare more effective, less harmful, and less expensive [6]. However, knowledge generation itself faces certain limitations. Much concern exists about poor design and deficient conduction and reporting of clinical research [7], not only because of methodological issues or its effects on scientific advancement but by its practical implications in health decision making, at either individual or population level [8]. Glasziou and Chalmers [8] estimated that 85% of the investments for clinical research end up being avoidable research waste, which may be derived from any step along the research process, by choosing research questions with low-priority, or assessing futile outcomes [3]; conducting research whose rationale of design and methods are inadequate [9]; dismissing the full dissemination of data, causing selective report bias [10]; making research findings unusable because of inadequate reporting (i.e., scientific writing and publishing issues) [11]; and disregarding regulation, governance, and management over clinical research development [12].

Governments, policy-makers, funders and researchers should have accurate and up-to-date pictures of health evidence so that funds and resources are used in the best possible way, which is attuned to the Strategy on Health Research released by the World Health Organization in 2012 [13]. Nevertheless, the use of rigorous clinical research evidence for both clinical practice and health policymaking is still limited [14–16]. The limited budgets for both clinical practice and research make prioritisation a mandatory step for funding agencies, especially governmental organisations. This prioritisation should be done mainly considering the needed research topics, methodological design, cost-effectiveness of the projects, and expected social value [17,18].

Bases of health scientific advances and funding allocation should tend towards (and be guided by) population needs and local decision-making requirements [17,18]. Although some frameworks have been proposed to evaluate research applications [19], the focus on academic background and scientific productivity during the project assessment seem to be predominant [20]. In practice, organisations summon expert panels to judge according to the aforementioned technical and academic merit measures, with heterogeneous considerations about cost, research gaps, social value or local impact[20].

The lack of a prioritised agenda in developing countries may generate a source of inequity, by letting third-party interests (i.e. global research funding, or the pharmaceutical industry from high-income countries) inhabit regional research planning [21–23], in a context of poor governance in this matter [12,24]. The strategic planning of state funding allocation in most countries can still improve the identification of local evidence gaps to be filled by new research, avoiding (and not funding) redundant research. In Chile, clinical research is funded through different government grants, in addition to other types of funding (private agencies,

For peer review only - http://bmjopen.bmj.com/site/about/guidelines.xhtml

For peer review only

competitive funding, pharmaceutical or medical device industry sponsoring, international grants, etc.) [25], but there is no comprehensive local diagnosis of recent clinical research that could inform prioritisation for future research funding.

This is a protocol for a government-funded project with the following objectives:

1. To identify and classify health research studies conducted in Chile or by authors whose affiliations are based in Chile.
2. To elaborate evidence maps for relevant health conditions in Chile, considering prioritised interventions and core outcome sets.
3. To populate the maps with the primary and secondary evidence and their risk of bias and to identify knowledge gaps and redundant research incorporating international evidence.

Methods and analysis

This section describes the methods for each of the objectives.

Objective 1: Identification and classification of Chilean clinical research

We will conduct a scoping review that will be reported following the Preferred Reporting Items for Systematic Reviews and Meta-Analyses extension for scoping reviews (PRISMA-ScR) [26]. For this protocol, we followed the guidance from the PRISMA-P extension for protocols [27].

Eligibility criteria:

We will consider any clinical study design, either conducted in Chile or at least one affiliation based in Chile. We define clinical studies as those focused on a clinical health topic describing, measuring or exploring a health-related outcome in humans. Considering that our focus in the following objectives will be the highest priority conditions of the last Chilean health reform in which the explicitly guaranteed health conditions were defined, we will consider studies published from 2000 onwards [28].

We will consider any descriptive, observational or experimental primary study design, including case reports, case series, cross-sectional studies, case-control studies, cohort studies, quasi-experimental studies, diagnostic or prognostic studies, mixed-methods studies, and clinical trials. We will also include different formats of evidence synthesis, including systematic reviews, scoping reviews, evidence maps, and overviews [29]. We will not include narrative reviews, editorials, correspondence, letters to the editor, opinion articles, conference proceedings, study protocols and preprint reports without peer review. We will also exclude studies whose observation units are biological samples, economic evaluations, modelling studies, validation of instruments, and ecological studies.

Search methods

Electronic search strategy

For peer review only

We will perform a comprehensive search in electronic databases. The search strategies will be designed to retrieve any clinical study, whether conducted in Chile or with at least one affiliation based in Chile, that has been published since 2000 to date without the restriction of language or publication status.

We will search the following databases to be used:

1. MEDLINE vía Ovid SP
2. Embase vía Elsevier
3. PsycINFO via ProQuest SP
4. Cumulative Index to Nursing and Allied Health Literature (CINAHL) via EBSCOhost
5. Latin American and Caribbean Literature in Health Sciences (LILACS)
6. Web of Science (WoS) via Clarivate.

Supplemental **Appendix A** provides details of the electronic search strategy for MEDLINE via Ovid. For our MEDLINE search, we added a highly sensitive filter to identify randomised trials developed by the Cochrane Collaboration [30] and a validated search filter to retrieve systematic reviews developed by the Scottish Intercollegiate Guidelines Network (SIGN) [31]. The strategy proposed was peer-reviewed by another information specialist prior to implementation using the PRESS checklist [32].

Hand search strategy

We will conduct complimentary hand searches in the following information sources with the same criteria reported in the electronic search:
We identified seven information resources considered relevant to identify all the evidence that has not been indexed in the databases described in the electronic search and additional four information resources necessary to retrieve trials, reviews, protocols or other types of evidence, published in peer reviewed journals, that contain the inclusion criteria.
1) ANID repository (http://repositorio.conicyt.cl/). Repository of the National Agency for Research and Development that contains research and innovation projects financed by this agency.
2) SciELO (https://scielo.org/es/). Scielo contains the full texts of Chilean Open Access scientific journals, from all areas of knowledge, including peer-reviewed scientific research.
3) Bibliographic catalogues of the Chilean universities and DIBAM (http://www.bncatalogo.gob.cl). This is a unified catalogue of all public institutions in Chile (public libraries, archives and museums) that includes bibliographic information on all national print journals.
4) Bibliographic and National Repository of the Ministry of Health of Chile (http://www.repositoriodigital.minsal.cl/). It contains all the research developed by the Ministry, including clinical practice guidelines, primary studies, population statistics, and other documents.
5) Directory of Open Access Journals (https://doaj.org/). Independent index containing peer-reviewed open access journals.
6) Latin American Repositories Network (http://repositorioslatinoamericanos.uchile.cl). Provides access to full-text electronic publications located in different repositories in Latin American countries.
7) Portal of academic journals of the Universidad de Chile (https://revistas.uchile.cl/); Chilean Academic Journals provides open access to more than 300 publications published

For peer review only

by universities, scientific societies, government agencies and Non-Government Organisations.
8) Google Scholar (https://scholar.google.com/)
9) PROSPERO (https://www.crd.york.ac.uk/prospero/).
10) World Health Organization International Clinical Trials Registry Platform (ICTRP, www.who.int/trialsearch/).
11) ClinicalTrials.gov (http://clinicaltrials.gov)
We will follow the same inclusion criteria for studies identified in electronic searches.

Study selection

Two reviewers will independently perform a title and abstract screening. A third reviewer will solve any discrepancies. We will retrieve the full text of each study applying our selection criteria to determine its final inclusion. Afterwards, two reviewers will screen references by full text, solving discrepancies by a third reviewer. For this process, we will use the Covidence platform [33]. We will present a PRISMA 2020 flow diagram showing the process of study selection [34].

Data extraction

Two reviewers will extract the following data from the included studies:

- Bibliographic data: full citation including the list of authors, journal, and date of publication.
- Type of evidence: Primary studies or secondary studies.
- Study design: Case reports, case series, cross-sectional studies, case-control studies, cohorts, quasi-experimental studies, diagnostic studies, prognostic studies, randomised trials, systematic reviews and other forms of synthesised evidence.
- Area of study: We will characterise the retrieved articles by discipline and area of study used by the Organisation for Economic Co-operation and Development (OECD) in their category scheme [35].
- Location of the study: Chile, other country, multicentric study.
- Authors and authorships: Affiliation (based in Chilean institution or not), gender and type of authorship (main author, last author, corresponding author, working groups authorships, among others).
- Diseases or health conditions addressed by each study according to the taxonomy developed by the Global Burden Disease [36].
- Funding: We will classify the type of funding of each article, public or private, competitive funding, pharmaceutical or medical device industry sponsoring, or international grants.
- Conflicts of interest: We will extract the authors' statements from each report, considering descriptions for each author if available.

We will enter the data into a data extraction form (based in Google Sheets, Google LLC)

Summary of data

We do not intend to perform a risk of bias assessment at this stage (see **Objective 3**). We will summarise the findings of each category (i.e. proportion of clinical trials, proportion of studies for each condition). Moreover we will illustrate the trends for each study design and disease category.

For peer review only

Objective 2: Elaboration of evidence matrices for relevant health conditions in Chile, considering prioritised interventions and core outcome sets.

We will elaborate evidence matrices for prioritised health conditions selected from those defined under the General Regime of the Health Guarantees Act (*Garantías Explícitas en Salud,* GES) that was stated in 2004 in Chile. This Regime commanded public and private health providers to guarantee access, opportunity, quality and financial protection for the most relevant programs, diseases or conditions [37]. This Regime contemplates 85 programs, diseases and conditions that have been selected considering the health situation of the population, the effectiveness of the interventions, their contribution to the extension or quality of life and, when possible, their cost effectiveness [38].

We extracted the list of health conditions and excluded those defined as programs (e.g. «Orthosis (or technical help) for people aged 65 years and older» which aims to improve independence in the elderly). These conditions were initially prioritised by the Chilean government based on a local study of the global burden of disease in 2007 [39]. In consultation with the Health Ministry, we decided to prioritise the conditions based on the 2019 report by the Global Burden of Disease (GBD) initiative as it is a more up-to-date resource for decision-making [40]. GBD is a consortium of more than 3,600 researchers who collect, analyse data and provide a tool to visualise the burden and health loss of people due to hundreds of health conditions. The available data is classified by country, incorporating the prevalence, risk factors and the relative harm that each condition causes [40]. We filtered the GBD database by year (2019), location (Chile), context (cause), measure (DALYs, Disability-Adjusted Life Years), and metric (number). We distinguished causes by age groups, and used data from all causes and both sexes. We excluded GES conditions that did not match precisely enough with a GBD cause, according to the consensus of the authors. We provide the details and judgements regarding the reasons for exclusions for each GES problem in **Appendix B**. Then, we selected the 20 conditions with the highest burden in Chile, among those included in GES. This selection closely matched the local study on burden of disease conducted in 2007 which informed decision-making during the constitution of GES [39]. See **Table 1** for the list of the top 20 conditions identified in this process.

Table 1. Prioritised conditions from the General Regime of the Health Guarantees Act (*Garantías Explícitas en Salud*, GES) according to the Global Burden of Disease (GBD) 2019 in Chile, in decreasing order by Disability-Adjusted Life Years (DALYs).

GES condition	Matching GBD cause (age group)	GBD 2019 DALYs, number (CI95%)
Myocardial infarction	Ischemic heart disease (all ages)	214819.57 (227068.32 to 0)
Type 2 diabetes	Diabetes mellitus type 2 (all ages)	170569.88 (213283.00 to 0.19)

For peer review only

Depression in people aged 15 years and over	Depressive disorders (20 plus)	121414.38 (169061.49 to 2.23E-06)
Chronic kidney disease stage 4 and 6	Chronic kidney disease (all ages)	101733.72 (110880.12 to 208.53)
Ischemic stroke in people aged 15 years and over	Ischemic stroke (20 plus)	100161.76 (109460.56 to 94.57)
Stomach cancer	Stomach cancer (all ages)	85929.07 (91647.46 to 1.26)
Lung cancer	Tracheal, bronchus, and lung cancer (all ages)	83674.49 (88943.91 to 0.0002)
Chronic obstructive pulmonary disease (outpatient management)	Chronic obstructive pulmonary disease (all ages)	83190.76 (96438.34 to 0.0002)
Alzheimer's disease and other dementias	Alzheimer's disease and other dementias (all ages)	72825.97 (154958.48 to 4.11)
Colorectal cancer in people aged 15 years and over	Colon and rectum cancer (20 plus)	70756.65 (75584.77 to 1.66)
Hip and/or knee osteoarthritis, mild or moderate, in people aged 55 years and over (medical management)	Osteoarthritis (55 plus)	55576.88 (112169.90 to 1.85)
Prevention of preterm birth	Neonatal preterm birth (all ages)	51479.73 (63435.89 to 0.16)
Chronic hepatitis C	Cirrhosis and other chronic liver diseases due to hepatitis C (all ages)	50969.21 (65323.67 to 0.0005)
Breast cancer in people aged 15 years and over	Breast cancer (20 plus)	47006.54 (51554.51 to 16.13)
Prostate cancer in people aged 15 years and over	Prostate cancer (20 plus)	45854.87 (54664.48 to 2.60)
Schizophrenia	Schizophrenia (all ages)	42501.32 (56297.82 to 0.24)
Bipolar disorder in people aged 15 years and over	Bipolar disorder (20 plus)	38096.12 (58487.76 to 8.43E-06)

For peer review only

Community-acquired pneumonia in people aged 65 years and over (outpatient management)	Lower respiratory infections (65 to 89 years)	34844.45 (38946.70 to 1.10)
Asthma in people aged 15 years and over	Asthma (20 plus)	32694.24 (45713.86 to 0.12)
Secondary subarachnoid haemorrhage to rupture of brain aneurysms	Subarachnoid haemorrhage (all ages)	28635.44 (31612.39 to 1089.41)

We will then conduct the following steps to complete the matrices that will be populated with evidence in the following objective.

Step 1: Identifying the main outcomes

The definition of main outcomes for each health condition is a complex and difficult task, considering that reported outcomes are prone to bias, and that many studies tend to report outcomes with positive or statistically significant findings [41].

For that reason, we will consider agreed standardised sets of outcomes, known as Core Outcome Sets (COS) for identifying the main outcomes. The COS represents a non-restrictive minimum set of outcomes to be assessed and reported in studies for every health condition, and they are permanently updated and revised by the COMET initiative (http://www.comet-initiative.org/). The COS includes outcomes that are most relevant to clinicians, decision makers, patients and carers. If COS are not available for an individual condition, we will build a set of outcomes based on consensus with the input of multidisciplinary experts and patients, considering the existing outcomes embedded in current GES clinical practice guidelines.

Step 2: Identifying the interventions

We will define the interventions for the rows of the matrix for each health condition considering:

- Local Clinical Practice Guidelines: The Chilean Health Ministry developed clinical practice guidelines comprehending primary, secondary and tertiary prevention and treatment for the main health conditions that are guaranteed. We will extract the main interventions from the clinical recommendations in existing guidelines.
- Those prioritised by Cochrane Review Groups and Networks: We will contact Cochrane Groups and Networks' authors in order to gather a list of priority interventions for each health condition, according to their consideration.
- Those identified by the regulatory agency: We will review the authorisations of the Institute of Public Health in Chile as the main regulatory agency in charge of the permits for clinical trials, in order to classify the authorised pharmacological interventions for each health condition.

Step 3: Building up the matrices

For peer review only

We will create evidence gap maps for each included condition. These evidence gap maps will be framed upon grids or matrices that will consider the relevant interventions (defined in **Step 3**) in the rows, and the main outcomes (defined in **Step 2**) in the columns. These grids will be populated in each intersection with the included studies, as we detail in Objective 3 (see **Objective 3** below). We will use evimappr [42], an R [43] package for producing bubble plots, which provides an interactive display for visualising the evidence gap maps.

Objective 3: To populate the maps with local primary and secondary evidence and their risk of bias and to identify knowledge gaps and redundant research incorporating international evidence.

After the completion of the tasks proposed in objective 1, we will select all the studies that might provide evidence for decision making regarding interventions, to populate each of the matrices elaborated as described in objective 2. Whenever an evidence matrix is populated with studies for a certain outcome and intervention, we will refer to it as an evidence map.

Step 1: Identifying the main study designs

From the complete set of research articles included in objective 1, we will separate all those studies with a design relevant for decision making regarding interventions:

1. Randomised controlled trials.
2. Non-randomised primary studies, including controlled clinical trials, quasi-experimental designs (e.g., interrupted time series, controlled before-after studies) and observational studies (cohort studies, case-control studies, case series, analytical cross-sectional studies).
3. Synthesised evidence relevant to interventions (systematic reviews, overviews).

Descriptive studies, qualitative or mixed-methods studies, diagnostic accuracy studies, narrative reviews, scoping reviews and evidence maps will not be considered for this objective as they do not provide evidence supporting the efficacy or effectiveness of interventions.

Step 2: Risk of bias assessment

To assess risk of bias of the included studies, we will use standardised tools for each methodological design: 1) Cochrane Risk of Bias tool for randomised clinical trials [44], 2) ROBINS-I for non-randomised primary studies [45], and 3) ROBIS for systematic reviews [46], We will skip this step in the case of overviews, since currently there is no validated tool for assessing risk of bias in this specific methodological design.

Step 3: Populating evidence gap maps

We will populate each node of the previously elaborated grids or matrices with the selected studies, considering the intervention and outcome on that node. We will represent the populated nodes with visual symbols (bubbles), with different colours according to the type of study and risk of bias, and different sizes according to the number of studies available. Once a matrix is populated with Chilean studies, we will refer to it as a ‘local evidence map’.

For peer review only - http://bmjopen.bmj.com/site/about/guidelines.xhtml

Step 4: Developing global certainty of the evidence gap maps

We will develop a second map for each prioritised condition populated with rigorous international research (defined as the availability of high or moderate certainty of evidence, for each specific clinical question, according to the GRADE approach [47], that we will refer to as a 'global evidence map'. For this purpose we will conduct a new search aimed at identifying systematic reviews using the GRADE approach, which is currently used for developing Chilean clinical practice guidelines. We will use appropriate filters for the identification of the abovementioned reviews [32]. We will populate these maps with the degree of certainty of evidence, so each node will be classified as "High certainty" or "Moderate certainty" (reflecting that mostly there is enough evidence); and "Low certainty", "Very low certainty", or "No evidence" (reflecting that more evidence is needed).

Step 5: Contrasting global certainty of the evidence maps with local maps

Once step 4 is concluded, we will have two maps for each condition: 1) A local evidence map (populated with local Chilean research), and 2) a global certainty of the evidence map (populated with certainties of evidence from international synthesised evidence). In order to identify knowledge gaps and estimate redundant research, we will contrast these two maps. Once we cross these two maps, we will classify each populated node of the local map as "adequate research" or "redundant research", while each empty node will be classified as "evidence gap", according to the criteria defined in **Table 2**.

For peer review only

Table 2. Possible scenarios in the evidence gap maps.

	**Global evidence map: very low to low certainty of the evidence (or no evidence)**	**Global evidence map: moderate to high certainty of the evidence**
**Local evidence map: No local evidence**	True evidence gap	False evidence gap (unless local conditions require context-specific research)
**Local evidence map: Available local evidence**	Adequate research	Adequate research (if local evidence was published before the review)<br><br>Redundant research(if local evidence was published after the review)

We will consider the absence of local evidence as a gap. If this gap is established in the context of low or very low certainty of evidence in the global map, or if there is no global evidence, we will consider it as a true gap. If the gap is established in the context of high or moderate certainty of evidence in the global gap, we will consider it in general as a false evidence gap.

If there is local research in the context of a low, very low certainty, or no evidence in the global map, we will consider the local research as adequate. If there is local research in the context of a high or moderate certainty of evidence in the global map, we will consider the research as adequate if it was published before the review that informed the certainty of evidence, or as redundant research if it was published after this review.

Patient and public involvement

No patients were involved in the development of this protocol.

Ethics and dissemination

No ethical approval is required to conduct this project. Our findings will be submitted to peer-reviewed journals, and scientific conferences. To amplify the impact of our work and to accomplish knowledge translation objectives, we will disseminate the results through different platforms, including academic social media, blogs, local press, among others. At the same time, we will aim to communicate the final results to local funding agencies and government stakeholders, in order to inform the elaboration of future national research agendas.

Discussion

After the completion of this proposed study, we will deliver an overall picture of the type of clinical research that is being conducted in Chile since 2000, and to elaborate evidence gap maps for the main health conditions guaranteed in Chile, with a detailed description of the most relevant interventions and outcomes and the type of clinical research in each node.

We will also study the evidence gaps for the 10 health conditions with the highest burden in Chile and compare them with the available research globally; as a way to establish the true gaps of clinical research, and the amount of redundant findings addressed in Chile or by authors affiliated to Chilean institutions.

A limitation of our protocol might be the use of the GBD 2019 estimations to prioritise the most important diseases or conditions in Chile, according to GES. As GES is based on policy and local definitions, and not totally on an epidemiological rationale, our conceptualisation may neglect some important clinical entities (mainly multicomponent diseases). Nevertheless, we have added appendices with the detailed pairingt process, the burden of disease, and the excluded health topics (and reason of exclusion). In order to operationalise our procedures, we also narrowed the scope of the gap maps development (objective 3) to clinical research centred on efficacy or effectiveness, excluding descriptive studies, qualitative or mixed-methods studies, and diagnostic accuracy studies, among others, which may constitute other methodological limitation. Furthermore, we will limit the evidence gap maps only to health interventions in prioritised outcomes, and then, by focusing on the 20 conditions with the highest burden in Chile, we will not address the whole extent of the research in locally-prioritised health conditions.

For peer review only

A main strength of our protocol is the comprehensiveness of our search strategy. The development of a peer-reviewed search strategy in major electronic databases complemented with an exhaustive hand search will allow us to probably identify the whole body of clinical evidence conducted locally. The hand search is crucial and constitutes an important source of information, considering that research in the Latin American Region might not be adequately indexed in the electronic databases. The process of development of evidence maps constitute another major strength of our proposed research. We will first develop the evidence grids identifying main outcomes and interventions independently of the results of our search and screening process (objective 2), and we will populate those grids afterwards with the results of our selection process (objective 3). Besides identifying the research being conducted in Chile, with this approach we will be able to accurately elucidate the evidence gaps, since it is possible that particular interventions in the grid remain unpopulated, as the construction of the grid will not be guided by the results of our search.

This accurate and up-to-date picture of the research gaps—to be filled by new future findings—and the identification of redundant research, will constitute relevant information for local decision-makers and, at the same time, an interesting methodological proposal to visualise clinical research trends and gaps in other countries or regions.

Acknowledgments: We thank Ivan Solà from the Iberoamerican Cochrane Centre - Sant Pau Biomedical Research Institute (IIB Sant Pau) - CIBERESP (Barcelona, Spain) for his valuable support in drafting and refining our search strategy.

Authors' contributions: The study concept was developed by JB, NM, JVAF and EM. The manuscript of the protocol was drafted by JB, NM, JVAF, CME-L and EM. SM and GU developed and provided feedback for all sections of the review protocol and approved the final manuscript. The search strategy was developed by CME-L and JB. Study selection, data extraction, quality assessment and gap maps elaboration will be performed by all the authors. All authors reviewed and approved the final version of the manuscript.
Funding: FONDECYT Grant 1212037 from the Chilean National Agency of Research and Development (ANID). The funding agency had no involvement in the conception, development, drafting or approval of this manuscript.
Competing interests: All the authors have conducted clinical research in Chile or associated with Chilean researchers or institutions. Nevertheless, decisions about inclusion, data extraction and quality assessment of these studies, will be conducted by independent researchers within our team. We declare no other conflict of interests.
Patient consent for publication: Not required.
Provenance and peer review: Not commissioned; externally peer-reviewed.
Data sharing statements: No additional data are available.

References

1 Vandvik PO, Brandt L, Alonso-Coello P, *et al.* Creating Clinical Practice Guidelines We Can Trust, Use, and Share. *Chest* 2013;**144**:381–9. doi:10.1378/chest.13-0746
2 Vandvik PO, Brandt L. Future of Evidence Ecosystem Series: Evidence ecosystems and learning health systems: why bother? *Journal of Clinical Epidemiology* 2020;**123**:166–70. doi:10.1016/j.jclinepi.2020.02.008

For peer review only

3 Chalmers I, Bracken MB, Djulbegovic B, *et al. How to increase value and reduce waste when research priorities are set*. Elsevier 2014. doi:10.1016/S0140-6736(13)62229-1
4 Dechartres A, Ravaud P. *Better prioritization to increase research value and decrease waste*. BioMed Central 2015. doi:10.1186/s12916-015-0492-3
5 Cartabellotta A, Tilson JK. The ecosystem of evidence cannot thrive without efficiency of knowledge generation, synthesis, and translation. *Journal of Clinical Epidemiology* 2019;**110**:90–5. doi:10.1016/j.jclinepi.2019.01.008
6 World Health Organization. *The WHO strategy on research for health*. World Health Organization 2012.
7 Ioannidis JPA, Greenland S, Hlatky MA, *et al. Increasing value and reducing waste in research design, conduct, and analysis*. Elsevier 2014. doi:10.1016/S0140-6736(13)62227-8
8 Glasziou P, Chalmers I. Research waste is still a scandal- An essay by Paul Glasziou and Iain Chalmers. *The BMJ* 2018;**363**:10–2. doi:10.1136/bmj.k4645
9 Ioannidis JPA, Greenland S, Hlatky MA, *et al. Increasing value and reducing waste in research design, conduct, and analysis*. Elsevier 2014. doi:10.1016/S0140-6736(13)62227-8
10 Chan A-W, Song F, Vickers A, *et al.* Increasing value and reducing waste: addressing inaccessible research. *The Lancet* 2014;**383**:257–66. doi:10.1016/S0140-6736(13)62296-5
11 Glasziou P, Altman DG, Bossuyt P, *et al.* Reducing waste from incomplete or unusable reports of biomedical research. *The Lancet* 2014;**383**:267–76. doi:10.1016/S0140-6736(13)62228-X
12 Salman RA-S, Beller E, Kagan J, *et al.* Increasing value and reducing waste in biomedical research regulation and management. *The Lancet* 2014;**383**:176–85. doi:10.1016/S0140-6736(13)62297-7
13 World Health Organization. Research and development to meet health needs in developing countries: strengthening global financing and coordination: report of the consultative expert working group on research and development: financing and coordination. 2012.
14 Brownson RC, Fielding JE, Maylahn CM. Evidence-Based Public Health: A Fundamental Concept for Public Health Practice. *Annu Rev Public Health* 2009;**30**:175–201. doi:10.1146/annurev.publhealth.031308.100134
15 Lester L, Haby MM, Chapman E, *et al.* Evaluation of the performance and achievements of the WHO Evidence-informed Policy Network (EVIPNet) Europe. In Review 2020. doi:10.21203/rs.2.21663/v1
16 van de Goor I, Hämäläinen R-M, Syed A, *et al.* Determinants of evidence use in public health policy making: Results from a study across six EU countries. *Health Policy* 2017;**121**:273–81. doi:10.1016/j.healthpol.2017.01.003
17 Ciarli T, Ràfols I. The relation between research priorities and societal demands: The case of rice. *Research Policy* 2019;**48**:949–67. doi:10.1016/j.respol.2018.10.027
18 Mutapi F. Africa should set its own health-research agenda. *Nature* 2019;**575**:567. doi:10.1038/d41586-019-03627-9
19 Tuffaha HW, Aitken J, Chambers S, *et al.* A Framework to Prioritise Health Research Proposals for Funding: Integrating Value for Money. *Appl Health Econ Health Policy* 2019;**17**:761–70. doi:10.1007/s40258-019-00495-2
20 Cassi L, Lahatte A, Rafols I, *et al.* Improving fitness: Mapping research priorities against societal needs on obesity. *Journal of Informetrics* 2017;**11**:1095–113. doi:10.1016/j.joi.2017.09.010
21 Aguilera B. Trends in clinical trials performed in Chile. *Rev méd Chile* 2021;**149**:110–8. doi:10.4067/S0034-98872021000100110

For peer review only

22 Atal I, Trinquart L, Porcher R, *et al.* Differential Globalization of Industry- and Non-Industry–Sponsored Clinical Trials. *PLoS ONE* 2015;**10**:e0145122. doi:10.1371/journal.pone.0145122
23 Hoffman SJ, Gunn E, Rogers Van Katwyk S, *et al.* Systematic analysis of global health research funding in Canada, 2000–2016. *Can J Public Health* 2020;**111**:80–95. doi:10.17269/s41997-019-00247-8
24 Smits P, Champagne F. Governance of health research funding institutions: an integrated conceptual framework and actionable functions of governance. *Health Res Policy Sys* 2020;**18**:22. doi:10.1186/s12961-020-0525-z
25 Agencia Nacional de Investigación y Desarrollo, Ministerio de Ciencia, Tecnología, Conocimiento e Innovación, Chile. ANID, Concursos. 2021.https://www.anid.cl/concursos/ (accessed 2 Jul 2021).
26 Tricco AC, Lillie E, Zarin W, *et al.* PRISMA Extension for Scoping Reviews (PRISMA-ScR): Checklist and Explanation. *Ann Intern Med* 2018;**169**:467–73. doi:10.7326/M18-0850
27 Moher D, Shamseer L, Clarke M, *et al.* Preferred reporting items for systematic review and meta-analysis protocols (PRISMA-P) 2015 statement. *Syst Rev* 2015;**4**:1. doi:10.1186/2046-4053-4-1
28 Bastias G, Pantoja T, Leisewitz T, *et al.* Health care reform in Chile. *Canadian Medical Association Journal* 2008;**179**:1289–92. doi:10.1503/cmaj.071843
29 Franco JVA, Arancibia M, Simancas-Racines D, *et al.* Syntheses of biomedical information: narrative reviews, systematic reviews and emerging formats. *Medwave* 2018;**18**:e7354–e7354. doi:10.5867/medwave.2018.07.7354
30 Lefebvre C, Glanville J, Briscoe S, *et al.* Technical Supplement to Chapter 4: Searching for and selecting studies. In: *Cochrane Handbook for Systematic Reviews of Interventions Version 6.2 (updated February 2021)*. Cochrane 2021. www.training.cochrane.org/handbook. (accessed 3 Sep 2021).
31 SIGN. Search filters. Scottish Intercollegiate Guidelines Network (SIGN). https://www.sign.ac.uk/what-we-do/methodology/search-filters/ (accessed 3 Sep 2021).
32 McGowan J, Sampson M, Salzwedel DM, *et al.* PRESS Peer Review of Electronic Search Strategies: 2015 Guideline Statement. *Journal of Clinical Epidemiology* 2016;**75**:40–6. doi:10.1016/j.jclinepi.2016.01.021
33 Veritas Health Innovation. *Covidence*. Melbourne, Australia: www.covidence.org
34 Page MJ, McKenzie JE, Bossuyt PM, *et al.* The PRISMA 2020 statement: an updated guideline for reporting systematic reviews. *BMJ* 2021;:n71. doi:10.1136/bmj.n71
35 OECD Category Scheme. http://help.prod-incites.com/inCites2Live/filterValuesGroup/researchAreaSchema/oecdCategoryScheme.html (accessed 14 Jul 2020).
36 Lozano R, Naghavi M, Foreman K, *et al.* Global and regional mortality from 235 causes of death for 20 age groups in 1990 and 2010: a systematic analysis for the Global Burden of Disease Study 2010. *The Lancet* 2012;**380**:2095–128. doi:10.1016/S0140-6736(12)61728-0
37 Superintendencia de Salud, MINSAL. Ley N° 19.966. Superintendencia de Salud, MINSAL. 2004.https://www.supersalud.gob.cl/normativa/668/w3-article-554.html (accessed 1 Sep 2021).
38 Urriola C, Infante A, Aguilera I, *et al.* La reforma de salud chilena a diez años de su implementación. *Salud Publica de Mexico* 2016;**58**:514–21. doi:10.21149/spm.v58i5.8240
39 Departamento de Epidemiología, MINSAL. Estudio Carga de Enfermedad y Carga atribuible. 2007.http://epi.minsal.cl/estudio-de-carga-de-enfermedades/ (accessed 1 Sep 2021).

For peer review only

40 Vos T, Lim SS, Abbafati C, *et al.* Global burden of 369 diseases and injuries in 204 countries and territories, 1990–2019: a systematic analysis for the Global Burden of Disease Study 2019. *The Lancet* 2020;**396**:1204–22. doi:10.1016/S0140-6736(20)30925-9
41 Dwan K, Altman DG, Arnaiz JA, *et al.* Systematic Review of the Empirical Evidence of Study Publication Bias and Outcome Reporting Bias. *PLoS ONE* 2008;**3**:e3081. doi:10.1371/journal.pone.0003081
42 Haddaway N, Keenan C. *evimappr: an R package for creating bubble plots for evidence maps.* 2021. https://github.com/nealhaddaway/evimappr/
43 R Core Team. *R: A language and environment for statistical computing*. 2021. https://www.R-project.org
44 Higgins JPT, Altman DG, Gotzsche PC, *et al.* The Cochrane Collaboration's tool for assessing risk of bias in randomised trials. *BMJ* 2011;**343**:d5928–d5928. doi:10.1136/bmj.d5928
45 Sterne JAC, Savović J, Page MJ, *et al.* RoB 2: a revised tool for assessing risk of bias in randomised trials. *BMJ* 2019;:l4898. doi:10.1136/bmj.l4898
46 Whiting P, Savović J, Higgins JPT, *et al.* ROBIS: A new tool to assess risk of bias in systematic reviews was developed. *Journal of Clinical Epidemiology* 2016;**69**:225–34. doi:10.1016/j.jclinepi.2015.06.005
47 Guyatt G, Oxman AD, Akl EA, *et al.* GRADE guidelines: 1. Introduction—GRADE evidence profiles and summary of findings tables. *Journal of Clinical Epidemiology* 2011;**64**:383–94. doi:10.1016/j.jclinepi.2010.04.026

For peer review only

Appendix A. Search strategy for a scoping review protocol for trends in Chilean clinical research

MEDLINE Ovid search strategy

Ovid MEDLINE(R) and Epub Ahead of Print, In-Process, In-Data-Review & Other Non-Indexed Citations, Daily and Versions(R) <1946 to June 17, 2021>

#1	Chile.ia,in.
#2	exp Chile/
#3	((Hospital or Clinic* or funda* or universi*) adj3 (Arica or Tarapaca or Antofagasta or Atacama or Coquimbo or Valparaiso or "Metropolitana de Santiago" or O Higgins or Maule or nuble or Biobio or Araucania or Los Rios or Los Lagos or Aysen or Magallanes)).ia,in.
#4	1 or 2 or 3
#5	randomized controlled trial.pt.
#6	controlled clinical trial.pt.
#7	randomized.ab.
#8	placebo.ab.
#9	drug therapy.fs.
#10	randomly.ab.
#11	trial.ab.
#12	groups.ab.
#13	5 or 6 or 7 or 8 or 9 or 10 or 11 or 12
#14	exp animals/ not humans.sh.
#15	13 not 14
#16	4 and 15
#17	Meta-Analysis as Topic/
#18	meta analy$.tw.
#19	metaanaly$.tw.
#20	Meta-Analysis/

For peer review only

#21	(systematic adj (review$1 or overview$1)).tw.
#22	exp Review Literature as Topic/
#23	17 or 18 or 19 or 20 or 21 or 22
#24	cochrane.ab.
#25	embase.ab.
#26	(psychlit or psyclit).ab.
#27	(psychinfo or psycinfo).ab.
#28	(cinahl or cinhal).ab.
#29	science citation index.ab.
#30	bids.ab.
#31	cancerlit.ab.
#32	24 or 25 or 16 or 27 or 28 or 29 or 30 or 31
#33	reference list$.ab.
#34	bibliograph$.ab.
#35	hand-search$.ab.
#36	relevant journals.ab.
#37	manual search$.ab.
#38	33 or 34 or 35 or 36 or 37
#39	selection criteria.ab.
#40	data extraction.ab.
#41	39 or 40
#42	Review/
#43	41 and 42
#44	Comment/
#45	Letter/
#46	Editorial/
#47	animal/

For peer review only

#48	human/
#49	47 not (47 and 48)
#50	44 or 45 or 46 or 49
#51	23 or 32 or 38 or 43 or 50
#52	4 and 51
#53	16 or 52
#54	limit 53 to yr=2000-2021

#1	exp cohort studies/
#2	cohort$.tw.
#3	exp Observational Study/
#4	observational.ab,ti.
#5	exp Cross-Sectional Studies/
#6	Prevalence Stud*.ti,ab.
#7	Cross Sectional*.ti,ab.
#8	descriptive.mp.
#9	Case.ti,ab. AND (report*.ti,ab. OR case serie*.ti,ab.)
#10	exp Diagnosis/
#11	Diagnos.ti,ab
#12	1 or 2 or 3 or 4 or 5 or 6 or 7 or 8 or 9 or 10 or 11 or 12
#13	Chile.ia,in.
#14	exp Chile/
#15	((Hospital or Clinic* or funda* or universi*) adj3 (Arica or Tarapaca or Antofagasta or Atacama or Coquimbo or Valparaiso or "Metropolitana de Santiago" or O Higgins or Maule or nuble or Biobio or Araucania or Los Rios or Los Lagos or Aysen or Magallanes)).ia,in.
#16	13 or 14 or 15
#17	12 and 16
#18	limit 17 to yr=2000-2021

For peer review only

Appendix B. Potentially eligible and excluded conditions/programs from the General Regime of the Health Guarantees Act (*Garantías Explícitas en Salud*, GES).

Appendix B.1. Potentially eligible conditions from GES, in descending order according to Global Burden of Disease 2019 (GBD 2019) Disability-Adjusted Life Years (DALYs).

GES condition	Matching GBD 2019 cause (age groups)	DALYs, number (CI%95)
Myocardial infarction	Ischemic heart disease (all ages)	214819.571800329 (227068.320701352 to 0)
Type 2 diabetes	Diabetes mellitus type 2 (all ages)	170569.880724338 (213283.001985072 to 0.192722088496509)
Depression in people aged 15 years and over	Depressive disorders (20 plus)	121414.377867517 (169061.491027657 to 2.2253601218192E-06)
Chronic kidney disease stage 4 and 6	Chronic kidney disease (all ages)	101733.722907926 (110880.123710307 to 208.525669506703)
Ischemic stroke in people aged 15 years and over	Ischemic stroke (20 plus)	100161.760973747 (109460.558336013 to 94.57213560342)
Stomach cancer	Stomach cancer (all ages)	85929.065773134 (91647.4644054603 to 1.26268744062835)

For peer review only

Lung cancer	Tracheal, bronchus, and lung cancer (all ages)	83674.4888793253 (88943.9075435297 to 0.000258752006588802)
Chronic obstructive pulmonary disease (outpatient management)	Chronic obstructive pulmonary disease (all ages)	83190.7630904602 (96438.3426616626 to 0.000164947566358871)
Alzheimer's disease and other dementias	Alzheimer's disease and other dementias (all ages)	72825.9715672292 (154958.475973897 to 4.10904084133898)
Colorectal cancer in people aged 15 years and over	Colon and rectum cancer (20 plus)	70756.6453332632 (75584.7656820732 to 1.65874626595222)
Operable congenital heart defect in people younger than 15 years	Congenital birth defects (0 to 14)	60558.1526518899 (79184.2191913363 to 3.88473612239553E-06)
Hip and/or knee osteoarthritis, mild or moderate, in people aged 55 years and over (medical management)	Osteoarthritis (55 plus)	55576.8772705614 (112169.901045112 to 1.84635512150662)
Prevention of preterm birth	Neonatal preterm birth (all ages)	51479.7300991547 (63435.89308863 to 0.158666035639263)
Chronic hepatitis C	Cirrhosis and other chronic liver diseases due to hepatitis C (all ages)	50969.2141174526 (65323.6655151894 to 0.000549020300847728)
Breast cancer in people aged 15 years and over	Breast cancer (20 plus)	47006.5369289545 (51554.507927785 to 16.1265082764972)
Prostate cancer in people aged 15 years and over	Prostate cancer (20 plus)	45854.8719687154 (54664.4754416884 to 2.59747447089968)

For peer review only

Schizophrenia	Schizophrenia (all ages)	42501.3194923616 (56297.8169798092 to 0.240749074074181)
Bipolar disorder in people aged 15 years and over	Bipolar disorder (20 plus)	38096.1151342666 (58487.7573236035 to 8.43274832801397E-06)
Community-acquired pneumonia in people aged 65 years and over (outpatient management)	Lower respiratory infections (65 to 89 years)	34844.453660895 (38946.6963391689 to 1.10402107319051)
Asthma in people aged 15 years and over	Asthma (20 plus)	32694.2377867036 (45713.8574539779 to 0.116280132000234)
Secondary subarachnoid hemorrhage to rupture of brain aneurysms	Subarachnoid hemorrhage (all ages)	28635.4404153411 (31612.3869284669 to 1089.40824984095)
Human Immunodeficiency Virus and Acquired Immunodeficiency Syndrome (HIV/AIDS)	HIV/AIDS (all ages)	27181.8681774182 (32191.0340641685 to 29.3017079071855)
Cervical cancer	Cervical cancer (all ages)	25200.4297936711 (30005.252368359 to 0.000474071744494449)
Non-refractory epilepsy in people aged 15 years and older	Idiopathic epilepsy (20 plus)	24620.5412700062 (41485.3459737919 to 3501.83306715692)
Kidney cancer in people aged 15 years and older	Kidney cancer (20 plus)	23991.4065239036 (26418.6543665931 to 534.114744605753)
Leukemia in people aged 15 years and older	Leukemia (20 plus)	21325.5560639148 (22901.6129356555 to 0.000813097105958376)

For peer review only

Parkinson's disease	Parkinson's disease (all ages)	20331.9233118182 (22034.4067968826 to 147.770845873412)
Epithelial ovarian cancer	Ovarian cancer (20 plus)	15600.3173555778 (17963.5312380173 to 1407.16245468356)
Rheumatoid arthritis	Rheumatoid arthritis (all ages)	14399.6554552725 (18512.9913244069 to 0.00362617834828417)
Primary tumors of the central nervous system in people aged 15 years and older	Brain and central nervous system cancer (20 plus)	14245.6107807219 (16062.6268023673 to 133.432208773944)
Multiple myeloma in people aged 15 years and older	Multiple myeloma (20 plus)	13895.9089272371 (15829.801624441 to 9.75441705033119)
Chronic hepatitis due to hepatitis B virus	Cirrhosis and other chronic liver diseases due to hepatitis B (all ages)	13680.7698238002 (19065.5815024126 to 0.0085089965860338)
Bladder cancer in people aged 15 years and older	Bladder cancer (20 plus)	12707.3764274785 (14072.9882127301 to 3054.38153002352)
Cancer in children under the age of 15 years	Neoplasms (0 to 14)	12303.3788104734 (14469.5035669086 to 113.905690992672)
Total hip endoprothesis for people aged 65 years and older and arthrosis of the hip with severe functional limitations	Osteoarthritis hand (65 to 89 years)	10568.6092353729 (21500.8167895118 to 0)
Cataract surgery	Cataract (all ages)	9770.85045117638 (13317.247585107 to 3232.36904442194)

For peer review only

Moderate and acute bronchial asthma in children under the age of 15 years	Asthma (0 to 14)	7963.77834457529 (13656.3525907285 to 51.6514027019469)
Type I diabetes mellitus	Diabetes mellitus type 1 (all ages)	7429.36242517253 (9651.21392599779 to 0)
Testicular cancer in people aged 15 years and older	Testicular cancer (20 plus)	6425.06386606812 (7673.24210673214 to 0)
Refractory epilepsy in children between the ages of 1 and 15 years	Idiopathic epilepsy (0 to 14)	5569.06914520869 (11130.3966112371 to 105.052591863323)
Heavy consumption or low to moderate risk of addiction to alcohol and drugs in people under the age of 20 years	Substance use disorders (<20 years)	5312.55244366324 (7461.50867463031 to 698.314690531639)
Treatment for the eradication of *Helicobacter pylori*	Gastritis and duodenitis (all ages)	3833.44230358697 (5407.26107597058 to 31.0664601228219)
Differentiated and medullary thyroid cancer in people aged 15 years and older	Thyroid cancer (20 plus)	3524.95356842812 (3925.21814487822 to 0)
Treatment for benign prostatic hyperplasia in symptomatic people	Benign prostatic hyperplasia (all ages)	3136.15726142161 (4876.83538193793 to 0.00194715937414714)
Surgical treatment for chronic lesions of the mitral and tricuspid valves in people aged 15 years and older	Non-rheumatic degenerative mitral valve disease (20 plus)	2321.91890136389 (2772.42279203503 to 0.00640083277215629)
Refractive errors in people aged 65 years and older	Refraction disorders (65 to 89 years)	2267.63302256029 (3291.72011313406 to 4699.20120851181)
Relapsing-remitting multiple sclerosis	Multiple sclerosis (all ages)	2192.67301330785 (2860.98207134884 to

For peer review only

		0.000916848432839514)
Cholecystectomy for the prevention of gallbladder cancer in people between the ages of 35 and 49 years	Gallbladder and biliary diseases (25 to 49)	2061.70765625961 (2857.83007158215 to 7.89297979047496)
Outpatient care for Acute Respiratory Infection (ARI) in children under the age of 5 years	Upper respiratory infections (under 5)	1783.98509162834 (2869.84870415435 to 53.7737581195539)
Cleft lip and palate	Orofacial clefts (0 to 14)	66.8835836316003 (109.496663940256 to 17707.9341422313)

For peer review only

Appendix B.2. Excluded GES conditions

GES condition	Reason for exclusion
Primary or essential arterial hypertension in people aged 15 years and older	Matching condition not found in GBD 2019 data
Hemophilia	Matching condition not found in GBD 2019 data
Cystic fibrosis	Matching condition not found in GBD 2019 data
Juvenile idiopathic arthritis	Matching condition not found in GBD 2019 data
Hypothyroidism in people aged 15 years and older	Matching condition not found in GBD 2019 data.
Systemic lupus erythematosis	Matching condition not found in GBD 2019 data
Bilateral hearing loss in people aged 65 years and older requiring hearing aids	Matching condition not found in GBD 2019 data
Diabetic retinopathy	Matching condition not found in GBD 2019 data
Nontraumatic rhegmatogenous retinal detachment	Matching condition not found in GBD 2019 data
Scoliosis surgery for people under the age of 25 years	Matching condition not found in GBD 2019 data
Osteosarcoma in people aged 15 years and older	Matching condition not found in GBD 2019 data
Strabismus in children under the age of 9 years	Matching condition not found in GBD 2019 data
Hip dysplasia	Matching condition not found in GBD 2019 data
Treatment for moderate, severe and profound hearing loss in children under four	Matching condition not found in GBD 2019 data
Serious polytrauma	GES condition does not match with a unique cause in GBD 2019 data
Lymphomas in people aged 15 years and older	GES condition does not match with a unique cause in GBD 2019 data
Spinal dysraphism	Matching condition not found in GBD 2019 data

For peer review only

Surgical treatment of chronic lesions of the aortic valve in people aged 15 years and older	Matching condition not found in GBD 2019 data
Surgery for herniated nucleus pulposus	Matching condition not found in GBD 2019 data
Severe burns	Matching condition not found in GBD 2019 data
Serious eye trauma	Matching condition not found in GBD 2019 data
Impulse and conduction disorders in people aged 15 years and older who require a pacemaker	Matching condition not found in GBD 2019 data
Respiratory distress syndrome in the newborn	Matching condition not found in GBD 2019 data
Bilateral sensorineural hearing loss in premature babies	Matching condition not found in GBD 2019 data
Bronchopulmonary dysplasia in premature babies	Matching condition not found in GBD 2019 data
Retinopathy in premature babies	Matching condition not found in GBD 2019 data
Moderate and serious traumatic brain injury	Matching condition not found in GBD 2019 data

Appendix B.3. Excluded GES programs

Orthosis (or technical help) for people aged 65 years and older
Pain relief and palliative care for advanced cancer
Analgesia in childbirth
Integral dental health care for girls and boys aged 6 years old
Outpatient treatment for odontological emergencies
Integral dental health care for 60 year old adults
Integral dental health care for pregnant women
Secondary prevention of end-stage chronic kidney disease

For peer review only

PRISMA-P (Preferred Reporting Items for Systematic review and Meta-Analysis Protocols) 2015 checklist: recommended items to address in a systematic review protocol*

Section and topic	Item No	Checklist item	Page
**ADMINISTRATIVE INFORMATION**			
Title:			
Identification	1a	Identify the report as a protocol of a systematic review	1
Update	1b	If the protocol is for an update of a previous systematic review, identify as such	N/A
Registration	2	If registered, provide the name of the registry (such as PROSPERO) and registration number	N/A
Authors:			
Contact	3a	Provide name, institutional affiliation, e-mail address of all protocol authors; provide physical mailing address of corresponding author	2
Contributions	3b	Describe contributions of protocol authors and identify the guarantor of the review	15
Amendments	4	If the protocol represents an amendment of a previously completed or published protocol, identify as such and list changes; otherwise, state plan for documenting important protocol amendments	N/A
Support:			
Sources	5a	Indicate sources of financial or other support for the review	N/A
Sponsor	5b	Provide name for the review funder and/or sponsor	15
Role of sponsor or funder	5c	Describe roles of funder(s), sponsor(s), and/or institution(s), if any, in developing the protocol	15
**INTRODUCTION**			
Rationale	6	Describe the rationale for the review in the context of what is already known	3-4
Objectives	7	Provide an explicit statement of the question(s) the review will address with reference to participants, interventions, comparators, and outcomes (PICO)	5
**METHODS**			
Eligibility criteria	8	Specify the study characteristics (such as PICO, study design, setting, time frame) and report characteristics (such as years considered, language, publication status) to be used as criteria for eligibility for the review	5-6
Information sources	9	Describe all intended information sources (such as electronic databases, contact with study authors, trial registers or other grey literature sources) with planned dates of coverage	6-7
Search strategy	10	Present draft of search strategy to be used for at least one electronic database, including planned limits, such that it could be repeated	Appendix A

For peer review only

Study records:			
Data management	11a	Describe the mechanism(s) that will be used to manage records and data throughout the review	7-8
Selection process	11b	State the process that will be used for selecting studies (such as two independent reviewers) through each phase of the review (that is, screening, eligibility and inclusion in meta-analysis)	7
Data collection process	11c	Describe planned method of extracting data from reports (such as piloting forms, done independently, in duplicate), any processes for obtaining and confirming data from investigators	7-8
Data items	12	List and define all variables for which data will be sought (such as PICO items, funding sources), any pre-planned data assumptions and simplifications	7-8
Outcomes and prioritization	13	List and define all outcomes for which data will be sought, including prioritization of main and additional outcomes, with rationale	11
Risk of bias in individual studies	14	Describe anticipated methods for assessing risk of bias of individual studies, including whether this will be done at the outcome or study level, or both; state how this information will be used in data synthesis	13
Data synthesis	15a	Describe criteria under which study data will be quantitatively synthesised	
	15b	If data are appropriate for quantitative synthesis, describe planned summary measures, methods of handling data and methods of combining data from studies, including any planned exploration of consistency (such as I^2, Kendall's τ)	
	15c	Describe any proposed additional analyses (such as sensitivity or subgroup analyses, meta-regression)	
	15d	If quantitative synthesis is not appropriate, describe the type of summary planned	8, 13, 14
Meta-bias(es)	16	Specify any planned assessment of meta-bias(es) (such as publication bias across studies, selective reporting within studies)	N/A
Confidence in cumulative evidence	17	Describe how the strength of the body of evidence will be assessed (such as GRADE)	13-14

*** It is strongly recommended that this checklist be read in conjunction with the PRISMA-P Explanation and Elaboration (cite when available) for important clarification on the items. Amendments to a review protocol should be tracked and dated. The copyright for PRISMA-P (including checklist) is held by the PRISMA-P Group and is distributed under a Creative Commons Attribution Licence 4.0.**

From: Shamseer L, Moher D, Clarke M, Ghersi D, Liberati A, Petticrew M, Shekelle P, Stewart L, PRISMA-P Group. Preferred reporting items for systematic review and meta-analysis protocols (PRISMA-P) 2015: elaboration and explanation. BMJ. 2015 Jan 2;349(jan02 1):g7647.

For peer review only

BMJ Open

Mapping Chilean clinical research: a protocol for a scoping review and multiple evidence gap maps

Journal:	*BMJ Open*
Manuscript ID	bmjopen-2021-057555.R3
Article Type:	Protocol
Date Submitted by the Author:	19-May-2022
Complete List of Authors:	Bracchiglione, Javier; Universidad de Valparaíso, Interdisciplinary Centre for Health Studies (CIESAL)<br>Meza, Nicolás; Universidad de Valparaíso<br>Franco, Juan Victor Ariel; Hospital Italiano de Buenos Aires, Instituto Universitario; Instituto Universitario Hospital Italiano<br>Escobar Liquitay, Camila Micaela; Instituto Universitario Hospital Italiano de Buenos Aires, Research Department<br>Munoz, Sergio; Universidad de La Frontera, Public Heath<br>Urrutia, Gerard; Iberoamerican Cochrane Centre<br>Madrid, Eva; Universidad de Valparaiso, Escuela de Medicina
**Primary Subject Heading**:	Epidemiology
Secondary Subject Heading:	Health policy, Medical publishing and peer review, Health services research
Keywords:	PUBLIC HEALTH, STATISTICS & RESEARCH METHODS, HEALTH SERVICES ADMINISTRATION & MANAGEMENT

SCHOLARONE™
Manuscripts

Mapping Chilean clinical research: a protocol for a scoping review and multiple evidence gap maps

Authors:

Javier Bracchiglione
Interdisciplinary Centre for Health Studies (CIESAL), Universidad de Valparaíso, Viña del Mar, Chile
https://orcid.org/0000-0001-8738-2184

Nicolás Meza
Interdisciplinary Centre for Health Studies (CIESAL), Universidad de Valparaíso, Viña del Mar, Chile
https://orcid.org/0000-0001-9505-0358

Juan Victor Ariel Franco
Research Department, Instituto Universitario Hospital Italiano de Buenos Aires, Buenos Aires, Argentina
https://orcid.org/0000-0003-0411-899X

Camila Micaela Escobar-Liquitay
Research Department, Instituto Universitario Hospital Italiano de Buenos Aires, Buenos Aires, Argentina
https://orcid.org/0000-0002-2903-6870

Sergio Muñoz
Department of Public Health - CIGES, Faculty of Medicine, Universidad de La Frontera, Temuco, Chile
https://orcid.org/0000-0001-8383-6599

Gerard Urrútia
Iberoamerican Cochrane Centre - Sant Pau Biomedical Research Institute (IIB Sant Pau) - CIBERESP, Barcelona, Spain
https://orcid.org/0000-0002-8850-0960

Eva Madrid
Interdisciplinary Centre for Health Studies (CIESAL), Universidad de Valparaíso, Viña del Mar, Chile
https://orcid.org/0000-0002-8095-5549

Corresponding author: Nicolás Meza
Angamos 655 Edificio R2 Oficina 1107, Reñaca, Viña del Mar, Chile
nicolas.meza@uv.cl

Word count: 3591

For peer review only

Abstract

Introduction: Clinical research broadly aims to influence decision making in order to promote appropriate healthcare. Funding agencies should prioritise research projects according to needed research topics, methodological and cost-effectiveness considerations, and expected social value. In Chile, there is no local diagnosis regarding recent clinical research that might inform prioritisation for future research funding. This research aims to comprehensively identify and classify Chilean health research studies, elaborating evidence gap maps for the most burdensome local conditions.

Methods and analysis: We will search in electronic databases (MEDLINE, Embase, PsycINFO, CINAHL, LILACS, and WoS) and perform hand searches to retrieve, identify, and classify health research studies conducted in Chile or by authors whose affiliations are based in Chile, from 2000 onwards. We will elaborate evidence matrices for the 20 conditions with the highest burden in Chile (according to the Global Burden of Disease 2019) selected from those defined under the General Regime of the Health Guarantees Act. To elaborate the evidence gap maps, we will consider prioritised interventions and core outcome sets. To identify knowledge gaps and estimate redundant research, we will contrast these gap maps with the available international evidence of high or moderate certainty of evidence, for each specific clinical question. For this purpose, we will search systematic reviews using the GRADE approach.

Ethics and dissemination: No ethical approval is required to conduct this project. We will submit our results in both peer-reviewed journals and scientific conferences. We will aim to disseminate our findings through different academic platforms, social media, local press, among others. The final results will be communicated to local funding agencies and government stakeholders.

Discussion: We aim to provide an accurate and up-to-date picture of the research gaps—to be filled by new future findings—and the identification of redundant research, which will constitute relevant information for local decision-makers.

Keywords: clinical research, evidence map, scoping review, bibliometrics, Chile

For peer review only

Strengths and limitations of this study

- The protocol for this scoping review includes a comprehensive electronic and hand search strategy.
- We will be able to accurately elucidate local evidence gaps by first constructing evidence grids and then populating them with the results of our search.
- Considering that we used the estimations from the Global Burden of Disease 2019 for prioritising the most important diseases in Chile, we might be neglecting some important clinical entities, such as multicomponent diseases.
- We will develop gap maps only for the 20 conditions with the highest burden in Chile.

Introduction

Primary clinical research and synthesised evidence are the basis for knowledge translation, decision support and implementation in clinical settings, which should ultimately guide the generation of primary research (on prevention, aetiology, diagnosis, treatment and prognosis of any health condition or disease) [1,2].

Good quality clinical research, which must be set on priority questions [3,4], is essential for elucidating the best approaches to solve health challenges [5]; aiming at informing decision making to make healthcare more effective, less harmful, and less expensive [6]. However, much concern exists about poor design and deficient conduction and reporting [7], not only because of methodological issues or its effects on scientific advancement but by its practical implications, at either individual or population level [8]. Glasziou and Chalmers [8] estimated that 85% of the investments for clinical research end up being avoidable research waste, which may be derived from any step along the research process and its application (i.e. disregarding regulation, governance, and management over clinical research development) [3,9,10-12]. Stakeholders should have accurate and up-to-date pictures of evidence so that resources are used in the best possible way [13]. Nevertheless, the use of rigorous evidence for both clinical practice and health policymaking is still limited [14–16]. The limited budgets make prioritisation a mandatory step for funding agencies; which should be done mainly considering the needed research topics, cost-effectiveness, and expected social value [17,18].

Although some frameworks have been proposed to evaluate research applications [19], the focus on academic background and scientific productivity during the project assessment seem to be predominant [20]. In practice, organisations summon expert panels to judge according to the aforementioned technical and academic merit measures, with heterogeneous considerations about cost, research gaps, social value or local impact [20].

The lack of a prioritised agenda in developing countries may generate a source of inequity, by letting third-party interests (i.e. global research funding, or the pharmaceutical industry from high-income countries) inhabit regional research planning [21–23], in a context of poor governance in this matter [12,24]. The strategic planning of state funding allocation in most countries can still improve the identification of local evidence gaps to be filled by new research, avoiding (and not funding) redundant research. In Chile, clinical research is funded through different government grants, in addition to other types of funding (private agencies, competitive funding, pharmaceutical or medical device industry sponsoring, international grants, etc.) [25], but there is no comprehensive local diagnosis of recent clinical research that could inform prioritisation for future research funding.

This is a protocol for a government-funded project with the following objectives:

1. To identify and classify health research studies conducted in Chile or by authors whose affiliations are based in Chile.
2. To elaborate evidence maps for relevant health conditions in Chile, considering prioritised interventions and core outcome sets.
3. To populate the maps with the primary and secondary evidence and their risk of bias and to identify knowledge gaps and redundant research incorporating international evidence.

For peer review only - http://bmjopen.bmj.com/site/about/guidelines.xhtml

Methods and analysis

This section describes the methods for each of the objectives.

Objective 1: Identification and classification of Chilean clinical research

We will conduct a scoping review that will be reported following the Preferred Reporting Items for Systematic Reviews and Meta-Analyses extension for scoping reviews (PRISMA-ScR) [26]. For this protocol, we followed the guidance from the PRISMA-P extension for protocols [27].

Eligibility criteria:

We will consider any clinical study design, either conducted in Chile or at least one affiliation based in Chile. We define clinical studies as those focused on a clinical health topic describing, measuring or exploring a health-related outcome in humans. Considering that our focus in the following objectives will be the highest priority conditions of the last Chilean health reform, we will consider studies published from 2000 onwards [28].

We will consider any descriptive, observational or experimental primary study designs, including case reports, case series, cross-sectional studies, case-control studies, cohort studies, quasi-experimental studies, diagnostic or prognostic studies, mixed-methods studies, and clinical trials. We will also include different formats of evidence synthesis, including systematic reviews, scoping reviews, evidence maps, and overviews [29]. We will exclude narrative reviews, editorials, correspondence, letters to the editor, opinion articles, conference proceedings, study protocols and preprint reports without peer review. We will also exclude studies whose observation units are biological samples, economic evaluations, modelling studies, validation of instruments, and ecological studies.

Search methods

Electronic search strategy

We will perform a comprehensive search in electronic databases, without restriction of language or publication status.

We will search the following databases:

1. MEDLINE via Ovid SP
2. Embase via Elsevier
3. PsycINFO via ProQuest SP
4. Cumulative Index to Nursing and Allied Health Literature (CINAHL) via EBSCOhost
5. Latin American and Caribbean Literature in Health Sciences (LILACS)
6. Web of Science (WoS) via Clarivate.

Supplemental **Appendix A** provides details of the electronic search strategy for MEDLINE via Ovid. For our MEDLINE search, we added a highly sensitive filter to identify randomised

For peer review only

trials developed by the Cochrane Collaboration [30] and a validated search filter to retrieve systematic reviews developed by the Scottish Intercollegiate Guidelines Network (SIGN) [31]. The strategy proposed was peer-reviewed by another information specialist prior to implementation using the PRESS checklist [32].

Hand search strategy

We will conduct complimentary hand searches with the same criteria reported in the electronic search. We identified seven information resources considered relevant to identify all the evidence that has not been indexed in the databases described in the electronic search and additional four information resources necessary to retrieve trials, reviews or other types of evidence, published in peer reviewed journals, that meet the eligibility criteria (see Appendix B for further details)

Study selection

Two reviewers will independently perform a title and abstract screening. A third reviewer will solve any discrepancies. We will retrieve the full text of each study applying our selection criteria to determine its final inclusion. Afterwards, two reviewers will screen references by full text, solving discrepancies by a third reviewer. For this process, we will use the Covidence platform [33]. We will present a PRISMA 2020 flow diagram showing the process of study selection [34].

Data extraction

Two reviewers will extract the following data from the included studies:

- Bibliographic data: full citation including the list of authors, journal, and date of publication.
- Type of evidence: Primary studies or secondary studies.
- Study design: Case reports, case series, cross-sectional studies, case-control studies, cohorts, quasi-experimental studies, diagnostic studies, prognostic studies, randomised trials, systematic reviews and other forms of synthesised evidence.
- Area of study: We will characterise the retrieved articles by discipline and area of study used by the Organisation for Economic Co-operation and Development (OECD) in their category scheme [35].
- Location of the study: Chile, other country, multicentric study.
- Authors and authorships: Affiliation (based in Chilean institution or not), gender and type of authorship (main author, last author, corresponding author, working groups authorships, among others).
- Diseases or health conditions addressed by each study according to the taxonomy developed by the Global Burden Disease [36].
- Funding: We will classify the type of funding of each article, public or private, competitive funding, pharmaceutical or medical device industry sponsoring, or international grants.
- Conflicts of interest: We will extract the authors' statements from each report, considering descriptions for each author if available.

For peer review only

We will enter the data into a data extraction form (based in Google Sheets, Google LLC)

Summary of data

We do not intend to perform a risk of bias assessment at this stage (see **Objective 3**). We will summarise the findings of each category (i.e. proportion of clinical trials, proportion of studies for each condition). Moreover we will illustrate the trends for each study design and disease category.

Objective 2: Elaboration of evidence matrices for relevant health conditions in Chile, considering prioritised interventions and core outcome sets.

We will elaborate evidence matrices for prioritised health conditions selected from those defined under the General Regime of the Health Guarantees Act (*Garantías Explícitas en Salud,* GES) that was stated in 2004 in Chile. This Regime commanded public and private health providers to guarantee access, opportunity, quality and financial protection for the most relevant programs, diseases or conditions [37]. This Regime contemplates 85 programs, diseases and conditions that have been selected considering the health situation of the population, the effectiveness of the interventions, their contribution to the extension or quality of life and, when possible, their cost effectiveness [38].

We extracted the list of health conditions and excluded those defined as programs (e.g. «Orthosis (or technical help) for people aged 65 years and older» which aims to improve independence in the elderly). These conditions were initially prioritised by the Chilean government based on a local study of the global burden of disease in 2007 [39]. In consultation with the Health Ministry, we decided to prioritise the conditions based on the 2019 report by the Global Burden of Disease (GBD) initiative as it is a more up-to-date resource for decision-making [40]. GBD is a consortium of more than 3,600 researchers who collect, analyse data and provide a tool to visualise the burden and health loss of people due to hundreds of health conditions[40]. We filtered the GBD database by year (2019), location (Chile), context (cause), measure (DALYs, Disability-Adjusted Life Years), and metric (number). We distinguished causes by age groups, and used data from all causes and both sexes. We excluded GES conditions that did not match precisely enough with a GBD cause, according to the consensus of the authors. We provide the details and judgements regarding the reasons for exclusions for each GES problem in **Appendix C**. Then, we selected the 20 conditions with the highest burden in Chile, among those included in GES. This selection closely matched the local study on burden of disease conducted in 2007 which informed decision-making during the constitution of GES [39]. See **Table 1** for the list of the top 20 conditions identified in this process.

Table 1. Prioritised conditions from the General Regime of the Health Guarantees Act (*Garantías Explícitas en Salud*, GES) according to the Global Burden of Disease (GBD) 2019 in Chile, in decreasing order by Disability-Adjusted Life Years (DALYs).

GES condition	Matching GBD cause (age group)	GBD 2019 DALYs, number (CI95%, upper to lower)

For peer review only

Myocardial infarction	Ischemic heart disease (all ages)	214819.57 (227068.32 to 0)
Type 2 diabetes	Diabetes mellitus type 2 (all ages)	170569.88 (213283.00 to 0.19)
Depression in people aged 15 years and over	Depressive disorders (20 plus)	121414.38 (169061.49 to 2.23E-06)
Chronic kidney disease stage 4 and 6	Chronic kidney disease (all ages)	101733.72 (110880.12 to 208.53)
Ischemic stroke in people aged 15 years and over	Ischemic stroke (20 plus)	100161.76 (109460.56 to 94.57)
Stomach cancer	Stomach cancer (all ages)	85929.07 (91647.46 to 1.26)
Lung cancer	Tracheal, bronchus, and lung cancer (all ages)	83674.49 (88943.91 to 0.0002)
Chronic obstructive pulmonary disease (outpatient management)	Chronic obstructive pulmonary disease (all ages)	83190.76 (96438.34 to 0.0002)
Alzheimer's disease and other dementias	Alzheimer's disease and other dementias (all ages)	72825.97 (154958.48 to 4.11)
Colorectal cancer in people aged 15 years and over	Colon and rectum cancer (20 plus)	70756.65 (75584.77 to 1.66)
Hip and/or knee osteoarthritis, mild or moderate, in people aged 55 years and over (medical management)	Osteoarthritis (55 plus)	55576.88 (112169.90 to 1.85)
Prevention of preterm birth	Neonatal preterm birth (all ages)	51479.73 (63435.89 to 0.16)
Chronic hepatitis C	Cirrhosis and other chronic liver diseases due to hepatitis C (all ages)	50969.21 (65323.67 to 0.0005)
Breast cancer in people aged 15 years and over	Breast cancer (20 plus)	47006.54 (51554.51 to 16.13)
Prostate cancer in people aged 15 years and over	Prostate cancer (20 plus)	45854.87 (54664.48 to 2.60)

Schizophrenia	Schizophrenia (all ages)	42501.32 (56297.82 to 0.24)
Bipolar disorder in people aged 15 years and over	Bipolar disorder (20 plus)	38096.12 (58487.76 to 8.43E-06)
Community-acquired pneumonia in people aged 65 years and over (outpatient management)	Lower respiratory infections (65 to 89 years)	34844.45 (38946.70 to 1.10)
Asthma in people aged 15 years and over	Asthma (20 plus)	32694.24 (45713.86 to 0.12)
Secondary subarachnoid haemorrhage to rupture of brain aneurysms	Subarachnoid haemorrhage (all ages)	28635.44 (31612.39 to 1089.41)

We will then conduct the following steps to complete the matrices that will be populated with evidence in the following objective.

Step 1: Identifying the main outcomes

The definition of main outcomes for each health condition is a complex and difficult task, considering that reported outcomes are prone to bias, and that many studies tend to report outcomes with positive or statistically significant findings [41].

For that reason, we will consider agreed standardised sets of outcomes, known as Core Outcome Sets (COS) for identifying the main outcomes. The COS represents a non-restrictive minimum set of outcomes to be assessed and reported in studies for every health condition, and they are permanently updated and revised by the COMET initiative (http://www.comet-initiative.org/). The COS includes outcomes that are most relevant to clinicians, decision makers, patients and carers. If COS are not available for an individual condition, we will build a set of outcomes based on consensus with the input of multidisciplinary experts and patients, considering the existing outcomes embedded in current GES clinical practice guidelines.

Step 2: Identifying the interventions

We will define the interventions for the rows of the matrix for each health condition considering:

- Local Clinical Practice Guidelines: We will extract the main interventions from the clinical recommendations in existing guidelines conducted by the Chilean Health Ministry..
- Those prioritised by Cochrane Review Groups and Networks: We will contact Cochrane Groups and Networks' authors in order to gather a list of priority interventions for each health condition, according to their consideration.
- Those identified by the regulatory agency: We will review the authorisations of the Institute of Public Health in Chile as the main regulatory agency in charge of the

For peer review only

permits for clinical trials, in order to classify the authorised pharmacological interventions for each health condition.

Step 3: Building up the matrices

We will create evidence gap maps for each included condition. These evidence gap maps will be framed upon grids or matrices that will consider the relevant interventions (defined in **Step 3**) in the rows, and the main outcomes (defined in **Step 2**) in the columns. These grids will be populated in each intersection with the included studies, as we detail in Objective 3 (see **Objective 3** below). We will use evimappr [42], an R [43] package for producing bubble plots, which provides an interactive display for visualising the evidence gap maps.

Objective 3: To populate the maps with local primary and secondary evidence and their risk of bias and to identify knowledge gaps and redundant research incorporating international evidence.

After the completion of the tasks proposed in objective 1, we will select all the studies that might provide evidence for decision making regarding interventions, to populate each of the matrices elaborated as described in objective 2. Whenever an evidence matrix is populated with studies for a certain outcome and intervention, we will refer to it as an evidence map.

Step 1: Identifying the main study designs

From the complete set of research articles included in objective 1, we will separate all those studies with a design relevant for decision making regarding interventions:

1. Randomised controlled trials.
2. Non-randomised primary studies, including controlled clinical trials, quasi-experimental designs (e.g., interrupted time series, controlled before-after studies) and observational studies (cohort studies, case-control studies, analytical cross-sectional studies).
3. Synthesised evidence relevant to interventions (systematic reviews, overviews).

Descriptive studies, qualitative or mixed-methods studies, diagnostic accuracy studies, narrative reviews, scoping reviews and evidence maps will not be considered for this objective as they do not provide evidence supporting the efficacy or effectiveness of interventions.

Step 2: Risk of bias assessment

To assess risk of bias of the included studies, we will use standardised tools for each methodological design: 1) Cochrane Risk of Bias tool for randomised clinical trials [44], 2) ROBINS-I for non-randomised primary studies [45], and 3) ROBIS for systematic reviews [46], We will skip this step in the case of overviews, since currently there is no validated tool for assessing risk of bias in this specific methodological design.

Step 3: Populating evidence gap maps

We will populate each node of the previously elaborated grids or matrices with the selected studies, considering the intervention and outcome on that node. We will represent the

For peer review only

populated nodes with visual symbols (bubbles), with different colours according to the type of study and risk of bias, and different sizes according to the number of studies available. Once a matrix is populated with Chilean studies, we will refer to it as a 'local evidence map'.

Step 4: Developing global certainty of the evidence gap maps

We will develop a second map for each prioritised condition populated with rigorous international research (defined as the availability of high or moderate certainty of evidence), for each specific clinical question, according to the GRADE approach [47], that we will refer to as a 'global evidence map'. For this purpose we will conduct a new search aimed at identifying systematic reviews using the GRADE approach, using appropriate filters [32]. We will populate these maps with the degree of certainty of evidence, so each node will be classified as "High certainty" or "Moderate certainty" (reflecting that mostly there is enough evidence); and "Low certainty", "Very low certainty", or "No evidence" (reflecting that more evidence is needed).

Step 5: Contrasting global certainty of the evidence maps with local maps

Once step 4 is concluded, we will have two maps for each condition: 1) A local evidence map (populated with local Chilean research), and 2) a global certainty of the evidence map (populated with certainties of evidence from international synthesised evidence). In order to identify knowledge gaps and estimate redundant research, we will contrast these two maps. Once we cross these two maps, we will classify each populated node of the local map as "adequate research" or "redundant research", while each empty node will be classified as "evidence gap", according to the criteria defined in **Table 2**.

Table 2. Possible scenarios in the evidence gap maps.

	**Global evidence map: very low to low certainty of the evidence (or no evidence)**	**Global evidence map: moderate to high certainty of the evidence**
**Local evidence map: No local evidence**	True evidence gap	False evidence gap (unless local conditions require context-specific research)
**Local evidence map: Available local evidence**	Adequate research	Adequate research (if local evidence was published before the review)<br><br>Redundant research(if local evidence was published after the review)

We will consider the absence of local evidence as a gap. If this gap is established in the context of low or very low certainty of evidence in the global map, or if there is no global evidence, we will consider it as a true gap. If the gap is established in the context of high or

For peer review only

moderate certainty of evidence in the global gap, we will consider it in general as a false evidence gap.

If there is local research in the context of a low, very low certainty, or no evidence in the global map, we will consider the local research as adequate. If there is local research in the context of a high or moderate certainty of evidence in the global map, we will consider the research as adequate if it was published before the review that informed the certainty of evidence, or as redundant research if it was published after this review.

Patient and public involvement

No patients were involved in the development of this protocol.

Ethics and dissemination

No ethical approval is required to conduct this project. Our findings will be submitted to peer-reviewed journals, and scientific conferences. To amplify the impact of our work and to accomplish knowledge translation objectives, we will disseminate the results through different platforms, including academic social media, blogs, local press, among others. At the same time, we will aim to communicate the final results to local funding agencies and government stakeholders, in order to inform the elaboration of future national research agendas.

Discussion

After the completion of this proposed study, we will deliver an overall picture of the clinical research conducted in Chile since 2000, and we will elaborate evidence gap maps for the main health conditions guaranteed in Chile, with a detailed description of the most relevant interventions and outcomes and the type of clinical research in each node.

We will also study the evidence gaps for the 10 health conditions with the highest burden in Chile and compare them with the available research globally; as a way to establish the true gaps of clinical research, and the amount of redundant findings addressed in Chile or by authors affiliated to Chilean institutions.

A limitation of our protocol might be the use of the GBD 2019 estimations to prioritise the most important diseases or conditions in Chile, according to GES. As GES is based on policy and local definitions, and not totally on an epidemiological rationale, our conceptualisation may neglect some important clinical entities (mainly multicomponent diseases). Nevertheless, we have added appendices with the detailed pairing process, the burden of disease, and the excluded health topics (and reason of exclusion). To operationalise our procedures, we also narrowed the scope of the gap maps development (objective 3) to clinical research centred on efficacy or effectiveness, excluding descriptive studies, qualitative or mixed-methods studies, and diagnostic accuracy studies, among others. Furthermore, we will limit the evidence maps only to health interventions in prioritised outcomes, and then, by focusing on the 20 conditions with the highest burden in Chile, we will not address the whole extent of the research in locally-prioritised health conditions.

For peer review only

A main strength of our protocol is the comprehensiveness of our search strategy. The development of a peer-reviewed search strategy in major electronic databases complemented with an exhaustive hand search will allow us to probably identify the whole body of clinical evidence conducted locally. The hand search is crucial and constitutes an important source of information, considering that research in the Latin American Region might not be adequately indexed in the electronic databases. The process of development of evidence maps constitute another major strength of our proposed research. We will first develop the evidence grids identifying main outcomes and interventions independently of the results of our search and screening process (objective 2), and we will populate those grids afterwards with the results of our selection process (objective 3). Besides identifying the research being conducted in Chile, with this approach we will be able to accurately elucidate the evidence gaps, since it is possible that particular interventions in the grid remain unpopulated, as the construction of the grid will not be guided by the results of our search.

This accurate and up-to-date picture of the research gaps—to be filled by new future findings—and the identification of redundant research, will constitute relevant information for local decision-makers and, at the same time, an interesting methodological proposal to visualise clinical research trends and gaps in other countries or regions.

Acknowledgments: We thank Ivan Solà from the Iberoamerican Cochrane Centre - Sant Pau Biomedical Research Institute (IIB Sant Pau) - CIBERESP (Barcelona, Spain) for his valuable support in drafting and refining our search strategy.

Authors' contributions: The study concept was developed by JB, NM, JVAF and EM. The manuscript of the protocol was drafted by JB, NM, JVAF, CME-L and EM. SM and GU developed and provided feedback for all sections of the review protocol and approved the final manuscript. The search strategy was developed by CME-L and JB. Study selection, data extraction, quality assessment and gap maps elaboration will be performed by all the authors. All authors reviewed and approved the final version of the manuscript.
Funding: FONDECYT Grant 1212037 from the Chilean National Agency of Research and Development (ANID). The funding agency had no involvement in the conception, development, drafting or approval of this manuscript.
Competing interests: All the authors have conducted clinical research in Chile or associated with Chilean researchers or institutions. Nevertheless, decisions about inclusion, data extraction and quality assessment of these studies, will be conducted by independent researchers within our team. We declare no other conflict of interests.
Patient consent for publication: Not required.
Provenance and peer review: Not commissioned; externally peer-reviewed.
Data sharing statements: No additional data are available.

References

1 Vandvik PO, Brandt L, Alonso-Coello P, *et al.* Creating Clinical Practice Guidelines We Can Trust, Use, and Share. *Chest* 2013;**144**:381–9. doi:10.1378/chest.13-0746
2 Vandvik PO, Brandt L. Future of Evidence Ecosystem Series: Evidence ecosystems and learning health systems: why bother? *Journal of Clinical Epidemiology* 2020;**123**:166–70. doi:10.1016/j.jclinepi.2020.02.008

For peer review only

3 Chalmers I, Bracken MB, Djulbegovic B, *et al. How to increase value and reduce waste when research priorities are set*. Elsevier 2014. doi:10.1016/S0140-6736(13)62229-1
4 Dechartres A, Ravaud P. *Better prioritization to increase research value and decrease waste*. BioMed Central 2015. doi:10.1186/s12916-015-0492-3
5 Cartabellotta A, Tilson JK. The ecosystem of evidence cannot thrive without efficiency of knowledge generation, synthesis, and translation. *Journal of Clinical Epidemiology* 2019;**110**:90–5. doi:10.1016/j.jclinepi.2019.01.008
6 World Health Organization. *The WHO strategy on research for health*. World Health Organization 2012.
7 Ioannidis JPA, Greenland S, Hlatky MA, *et al. Increasing value and reducing waste in research design, conduct, and analysis*. Elsevier 2014. doi:10.1016/S0140-6736(13)62227-8
8 Glasziou P, Chalmers I. Research waste is still a scandal- An essay by Paul Glasziou and Iain Chalmers. *The BMJ* 2018;**363**:10–2. doi:10.1136/bmj.k4645
9 Ioannidis JPA, Greenland S, Hlatky MA, *et al. Increasing value and reducing waste in research design, conduct, and analysis*. Elsevier 2014. doi:10.1016/S0140-6736(13)62227-8
10 Chan A-W, Song F, Vickers A, *et al.* Increasing value and reducing waste: addressing inaccessible research. *The Lancet* 2014;**383**:257–66. doi:10.1016/S0140-6736(13)62296-5
11 Glasziou P, Altman DG, Bossuyt P, *et al.* Reducing waste from incomplete or unusable reports of biomedical research. *The Lancet* 2014;**383**:267–76. doi:10.1016/S0140-6736(13)62228-X
12 Salman RA-S, Beller E, Kagan J, *et al.* Increasing value and reducing waste in biomedical research regulation and management. *The Lancet* 2014;**383**:176–85. doi:10.1016/S0140-6736(13)62297-7
13 World Health Organization. Research and development to meet health needs in developing countries: strengthening global financing and coordination: report of the consultative expert working group on research and development: financing and coordination. 2012.
14 Brownson RC, Fielding JE, Maylahn CM. Evidence-Based Public Health: A Fundamental Concept for Public Health Practice. *Annu Rev Public Health* 2009;**30**:175–201. doi:10.1146/annurev.publhealth.031308.100134
15 Lester L, Haby MM, Chapman E, *et al.* Evaluation of the performance and achievements of the WHO Evidence-informed Policy Network (EVIPNet) Europe. In Review 2020. doi:10.21203/rs.2.21663/v1
16 van de Goor I, Hämäläinen R-M, Syed A, *et al.* Determinants of evidence use in public health policy making: Results from a study across six EU countries. *Health Policy* 2017;**121**:273–81. doi:10.1016/j.healthpol.2017.01.003
17 Ciarli T, Ràfols I. The relation between research priorities and societal demands: The case of rice. *Research Policy* 2019;**48**:949–67. doi:10.1016/j.respol.2018.10.027
18 Mutapi F. Africa should set its own health-research agenda. *Nature* 2019;**575**:567. doi:10.1038/d41586-019-03627-9
19 Tuffaha HW, Aitken J, Chambers S, *et al.* A Framework to Prioritise Health Research Proposals for Funding: Integrating Value for Money. *Appl Health Econ Health Policy* 2019;**17**:761–70. doi:10.1007/s40258-019-00495-2
20 Cassi L, Lahatte A, Rafols I, *et al.* Improving fitness: Mapping research priorities against societal needs on obesity. *Journal of Informetrics* 2017;**11**:1095–113. doi:10.1016/j.joi.2017.09.010
21 Aguilera B. Trends in clinical trials performed in Chile. *Rev méd Chile* 2021;**149**:110–8. doi:10.4067/S0034-98872021000100110

For peer review only

22 Atal I, Trinquart L, Porcher R, *et al.* Differential Globalization of Industry- and Non-Industry–Sponsored Clinical Trials. *PLoS ONE* 2015;**10**:e0145122. doi:10.1371/journal.pone.0145122
23 Hoffman SJ, Gunn E, Rogers Van Katwyk S, *et al.* Systematic analysis of global health research funding in Canada, 2000–2016. *Can J Public Health* 2020;**111**:80–95. doi:10.17269/s41997-019-00247-8
24 Smits P, Champagne F. Governance of health research funding institutions: an integrated conceptual framework and actionable functions of governance. *Health Res Policy Sys* 2020;**18**:22. doi:10.1186/s12961-020-0525-z
25 Agencia Nacional de Investigación y Desarrollo, Ministerio de Ciencia, Tecnología, Conocimiento e Innovación, Chile. ANID, Concursos. 2021.https://www.anid.cl/concursos/ (accessed 2 Jul 2021).
26 Tricco AC, Lillie E, Zarin W, *et al.* PRISMA Extension for Scoping Reviews (PRISMA-ScR): Checklist and Explanation. *Ann Intern Med* 2018;**169**:467–73. doi:10.7326/M18-0850
27 Moher D, Shamseer L, Clarke M, *et al.* Preferred reporting items for systematic review and meta-analysis protocols (PRISMA-P) 2015 statement. *Syst Rev* 2015;4:1. doi:10.1186/2046-4053-4-1
28 Bastias G, Pantoja T, Leisewitz T, *et al.* Health care reform in Chile. *Canadian Medical Association Journal* 2008;**179**:1289–92. doi:10.1503/cmaj.071843
29 Franco JVA, Arancibia M, Simancas-Racines D, *et al.* Syntheses of biomedical information: narrative reviews, systematic reviews and emerging formats. *Medwave* 2018;**18**:e7354–e7354. doi:10.5867/medwave.2018.07.7354
30 Lefebvre C, Glanville J, Briscoe S, *et al.* Technical Supplement to Chapter 4: Searching for and selecting studies. In: *Cochrane Handbook for Systematic Reviews of Interventions Version 6.2 (updated February 2021)*. Cochrane 2021. www.training.cochrane.org/handbook. (accessed 3 Sep 2021).
31 SIGN. Search filters. Scottish Intercollegiate Guidelines Network (SIGN). https://www.sign.ac.uk/what-we-do/methodology/search-filters/ (accessed 3 Sep 2021).
32 McGowan J, Sampson M, Salzwedel DM, *et al.* PRESS Peer Review of Electronic Search Strategies: 2015 Guideline Statement. *Journal of Clinical Epidemiology* 2016;**75**:40–6. doi:10.1016/j.jclinepi.2016.01.021
33 Veritas Health Innovation. *Covidence*. Melbourne, Australia: www.covidence.org
34 Page MJ, McKenzie JE, Bossuyt PM, *et al.* The PRISMA 2020 statement: an updated guideline for reporting systematic reviews. *BMJ* 2021;:n71. doi:10.1136/bmj.n71
35 OECD Category Scheme. http://help.prod-incites.com/inCites2Live/filterValuesGroup/researchAreaSchema/oecdCategoryScheme.html (accessed 14 Jul 2020).
36 Lozano R, Naghavi M, Foreman K, *et al.* Global and regional mortality from 235 causes of death for 20 age groups in 1990 and 2010: a systematic analysis for the Global Burden of Disease Study 2010. *The Lancet* 2012;**380**:2095–128. doi:10.1016/S0140-6736(12)61728-0
37 Superintendencia de Salud, MINSAL. Ley N° 19.966. Superintendencia de Salud, MINSAL. 2004.https://www.supersalud.gob.cl/normativa/668/w3-article-554.html (accessed 1 Sep 2021).
38 Urriola C, Infante A, Aguilera I, *et al.* La reforma de salud chilena a diez años de su implementación. *Salud Publica de Mexico* 2016;**58**:514–21. doi:10.21149/spm.v58i5.8240
39 Departamento de Epidemiología, MINSAL. Estudio Carga de Enfermedad y Carga atribuible. 2007.http://epi.minsal.cl/estudio-de-carga-de-enfermedades/ (accessed 1 Sep 2021).

For peer review only

40 Vos T, Lim SS, Abbafati C, *et al.* Global burden of 369 diseases and injuries in 204 countries and territories, 1990–2019: a systematic analysis for the Global Burden of Disease Study 2019. *The Lancet* 2020;**396**:1204–22. doi:10.1016/S0140-6736(20)30925-9
41 Dwan K, Altman DG, Arnaiz JA, *et al.* Systematic Review of the Empirical Evidence of Study Publication Bias and Outcome Reporting Bias. *PLoS ONE* 2008;3:e3081. doi:10.1371/journal.pone.0003081
42 Haddaway N, Keenan C. *evimappr: an R package for creating bubble plots for evidence maps.* 2021. https://github.com/nealhaddaway/evimappr/
43 R Core Team. *R: A language and environment for statistical computing*. 2021. https://www.R-project.org
44 Higgins JPT, Altman DG, Gotzsche PC, *et al.* The Cochrane Collaboration's tool for assessing risk of bias in randomised trials. *BMJ* 2011;**343**:d5928–d5928. doi:10.1136/bmj.d5928
45 Sterne JAC, Savović J, Page MJ, *et al.* RoB 2: a revised tool for assessing risk of bias in randomised trials. *BMJ* 2019;:l4898. doi:10.1136/bmj.l4898
46 Whiting P, Savović J, Higgins JPT, *et al.* ROBIS: A new tool to assess risk of bias in systematic reviews was developed. *Journal of Clinical Epidemiology* 2016;**69**:225–34. doi:10.1016/j.jclinepi.2015.06.005
47 Guyatt G, Oxman AD, Akl EA, *et al.* GRADE guidelines: 1. Introduction—GRADE evidence profiles and summary of findings tables. *Journal of Clinical Epidemiology* 2011;**64**:383–94. doi:10.1016/j.jclinepi.2010.04.026

For peer review only

Appendix A. Search strategy for a scoping review protocol for trends in Chilean clinical research

MEDLINE Ovid search strategy

Ovid MEDLINE(R) and Epub Ahead of Print, In-Process, In-Data-Review & Other Non-Indexed Citations, Daily and Versions(R) <1946 to June 17, 2021>

#1	Chile.ia,in.
#2	exp Chile/
#3	((Hospital or Clinic* or funda* or universi*) adj3 (Arica or Tarapaca or Antofagasta or Atacama or Coquimbo or Valparaiso or "Metropolitana de Santiago" or O Higgins or Maule or nuble or Biobio or Araucania or Los Rios or Los Lagos or Aysen or Magallanes)).ia,in.
#4	1 or 2 or 3
#5	randomized controlled trial.pt.
#6	controlled clinical trial.pt.
#7	randomized.ab.
#8	placebo.ab.
#9	drug therapy.fs.
#10	randomly.ab.
#11	trial.ab.
#12	groups.ab.
#13	5 or 6 or 7 or 8 or 9 or 10 or 11 or 12
#14	exp animals/ not humans.sh.
#15	13 not 14
#16	4 and 15
#17	Meta-Analysis as Topic/
#18	meta analy$.tw.
#19	metaanaly$.tw.

#20	Meta-Analysis/
#21	(systematic adj (review$1 or overview$1)).tw.
#22	exp Review Literature as Topic/
#23	17 or 18 or 19 or 20 or 21 or 22
#24	cochrane.ab.
#25	embase.ab.
#26	(psychlit or psyclit).ab.
#27	(psychinfo or psycinfo).ab.
#28	(cinahl or cinhal).ab.
#29	science citation index.ab.
#30	bids.ab.
#31	cancerlit.ab.
#32	24 or 25 or 16 or 27 or 28 or 29 or 30 or 31
#33	reference list$.ab.
#34	bibliograph$.ab.
#35	hand-search$.ab.
#36	relevant journals.ab.
#37	manual search$.ab.
#38	33 or 34 or 35 or 36 or 37
#39	selection criteria.ab.
#40	data extraction.ab.
#41	39 or 40
#42	Review/
#43	41 and 42
#44	Comment/
#45	Letter/

#46	Editorial/
#47	animal/
#48	human/
#49	47 not (47 and 48)
#50	44 or 45 or 46 or 49
#51	23 or 32 or 38 or 43 or 50
#52	4 and 51
#53	16 or 52
#54	limit 53 to yr=2000-2021

#1	exp cohort studies/
#2	cohort$.tw.
#3	exp Observational Study/
#4	observational.ab,ti.
#5	exp Cross-Sectional Studies/
#6	Prevalence Stud*.ti,ab.
#7	Cross Sectional*.ti,ab.
#8	descriptive.mp.
#9	Case.ti,ab. AND (report*.ti,ab. OR case serie*.ti,ab.)
#10	exp Diagnosis/
#11	Diagnos.ti,ab
#12	1 or 2 or 3 or 4 or 5 or 6 or 7 or 8 or 9 or 10 or 11 or 12
#13	Chile.ia,in.
#14	exp Chile/
#15	((Hospital or Clinic* or funda* or universi*) adj3 (Arica or Tarapaca or Antofagasta or Atacama or Coquimbo or Valparaiso or "Metropolitana de Santiago" or O Higgins or Maule or nuble or Biobio or Araucania or Los Rios or Los Lagos or Aysen or Magallanes)).ia,in.

#16	13 or 14 or 15
#17	12 and 16
#18	limit 17 to yr=2000-2021

Appendix B. Data sources for complimentary hand search strategy.

1) ANID repository (http://repositorio.conicyt.cl/). Repository of the National Agency for Research and Development that contains research and innovation projects financed by this agency.

2) SciELO (https://scielo.org/es/). Scielo contains the full texts of Chilean Open Access scientific journals, from all areas of knowledge, including peer-reviewed scientific research.
3) Bibliographic catalogues of the Chilean universities and DIBAM (http://www.bncatalogo.gob.cl). This is a unified catalogue of all public institutions in Chile (public libraries, archives and museums) that includes bibliographic information on all national print journals.

4) Bibliographic and National Repository of the Ministry of Health of Chile (http://www.repositoriodigital.minsal.cl/). It contains all the research developed by the Ministry, including clinical practice guidelines, primary studies, population statistics, and other documents.

5) Directory of Open Access Journals (https://doaj.org/). Independent index containing peer-reviewed open access journals.

6) Latin American Repositories Network (http://repositorioslatinoamericanos.uchile.cl). Provides access to full-text electronic publications located in different repositories in Latin American countries.

7) Portal of academic journals of the Universidad de Chile (https://revistas.uchile.cl/); Chilean Academic Journals provides open access to more than 300 publications published by universities, scientific societies, government agencies and Non-Government Organisations.

8) Google Scholar (https://scholar.google.com/).

9) PROSPERO (https://www.crd.york.ac.uk/prospero/).

10) World Health Organization International Clinical Trials Registry Platform (ICTRP, www.who.int/trialsearch/).

11) ClinicalTrials.gov (http://clinicaltrials.gov)

Appendix C. Potentially eligible and excluded conditions/programs from the General Regime of the Health Guarantees Act (*Garantías Explícitas en Salud*, GES).

Appendix C.1. Potentially eligible conditions from GES, in descending order according to Global Burden of Disease 2019 (GBD 2019) Disability-Adjusted Life Years (DALYs).

GES condition	Matching GBD 2019 cause (age groups)	DALYs, number (CI%95, upper to lower)
Myocardial infarction	Ischemic heart disease (all ages)	214819.57 (227068.320701352 to 0)
Type 2 diabetes	Diabetes mellitus type 2 (all ages)	170569.880724338 (213283.001985072 to 0.192722088496509)
Depression in people aged 15 years and over	Depressive disorders (20 plus)	121414.377867517 (169061.491027657 to 2.2253601218192E-06)
Chronic kidney disease stage 4 and 6	Chronic kidney disease (all ages)	101733.722907926 (110880.123710307 to 208.525669506703)
Ischemic stroke in people aged 15 years and over	Ischemic stroke (20 plus)	100161.760973747 (109460.558336013 to 94.57213560342)
Stomach cancer	Stomach cancer (all ages)	85929.065773134 (91647.4644054603 to

		1.26268744062835)
Lung cancer	Tracheal, bronchus, and lung cancer (all ages)	83674.4888793253 (88943.9075435297 to 0.000258752006588802)
Chronic obstructive pulmonary disease (outpatient management)	Chronic obstructive pulmonary disease (all ages)	83190.7630904602 (96438.3426616626 to 0.000164947566358871)
Alzheimer's disease and other dementias	Alzheimer's disease and other dementias (all ages)	72825.9715672292 (154958.475973897 to 4.10904084133898)
Colorectal cancer in people aged 15 years and over	Colon and rectum cancer (20 plus)	70756.6453332632 (75584.7656820732 to 1.65874626595222)
Hip and/or knee osteoarthritis, mild or moderate, in people aged 55 years and over (medical management)	Osteoarthritis (55 plus)	55576.8772705614 (112169.901045112 to 1.84635512150662)
Prevention of preterm birth	Neonatal preterm birth (all ages)	51479.7300991547 (63435.89308863 to 0.158666035639263)
Chronic hepatitis C	Cirrhosis and other chronic liver diseases due to hepatitis C (all ages)	50969.2141174526 (65323.6655151894 to 0.000549020300847728)
Breast cancer in people aged 15 years and over	Breast cancer (20 plus)	47006.5369289545 (51554.507927785 to 16.1265082764972)
Prostate cancer in people aged 15 years and over	Prostate cancer (20 plus)	45854.8719687154 (54664.4754416884 to 2.59747447089968)
Schizophrenia	Schizophrenia (all ages)	42501.3194923616

		(56297.8169798092 to 0.240749074074181)
Bipolar disorder in people aged 15 years and over	Bipolar disorder (20 plus)	38096.1151342666 (58487.7573236035 to 8.43274832801397E-06)
Community-acquired pneumonia in people aged 65 years and over (outpatient management)	Lower respiratory infections (65 to 89 years)	34844.453660895 (38946.6963391689 to 1.10402107319051)
Asthma in people aged 15 years and over	Asthma (20 plus)	32694.2377867036 (45713.8574539779 to 0.116280132000234)
Secondary subarachnoid hemorrhage to rupture of brain aneurysms	Subarachnoid hemorrhage (all ages)	28635.4404153411 (31612.3869284669 to 1089.40824984095)
Human Immunodeficiency Virus and Acquired Immunodeficiency Syndrome (HIV/AIDS)	HIV/AIDS (all ages)	27181.8681774182 (32191.0340641685 to 29.3017079071855)
Cervical cancer	Cervical cancer (all ages)	25200.4297936711 (30005.252368359 to 0.000474071744494449)
Non-refractory epilepsy in people aged 15 years and older	Idiopathic epilepsy (20 plus)	24620.5412700062 (41485.3459737919 to 3501.83306715692)
Kidney cancer in people aged 15 years and older	Kidney cancer (20 plus)	23991.4065239036 (26418.6543665931 to 534.114744605753)
Leukemia in people aged 15 years and older	Leukemia (20 plus)	21325.5560639148 (22901.6129356555 to 0.000813097105958376)

Parkinson’s disease	Parkinson's disease (all ages)	20331.9233118182 (22034.4067968826 to 147.770845873412)
Operable congenital heart defect in people younger than 15 years	Congenital heart anomalies (0 to 14)	15978.961606000155 (22470.79582735375 to 10367.285436039341)
Epithelial ovarian cancer	Ovarian cancer (20 plus)	15600.3173555778 (17963.5312380173 to 1407.16245468356)
Rheumatoid arthritis	Rheumatoid arthritis (all ages)	14399.6554552725 (18512.9913244069 to 0.00362617834828417)
Primary tumors of the central nervous system in people aged 15 years and older	Brain and central nervous system cancer (20 plus)	14245.6107807219 (16062.6268023673 to 133.432208773944)
Multiple myeloma in people aged 15 years and older	Multiple myeloma (20 plus)	13895.9089272371 (15829.801624441 to 9.75441705033119)
Chronic hepatitis due to hepatitis B virus	Cirrhosis and other chronic liver diseases due to hepatitis B (all ages)	13680.7698238002 (19065.5815024126 to 0.0085089965860338)
Bladder cancer in people aged 15 years and older	Bladder cancer (20 plus)	12707.3764274785 (14072.9882127301 to 3054.38153002352)
Cancer in children under the age of 15 years	Neoplasms (0 to 14)	12303.3788104734 (14469.5035669086 to 113.905690992672)
Total hip endoprothesis for people aged 65 years and older and arthrosis of the hip with severe functional limitations	Osteoarthritis hand (65 to 89 years)	10568.6092353729 (21500.8167895118 to 0)

Cataract surgery	Cataract (all ages)	9770.85045117638 (13317.247585107 to 3232.36904442194)
Moderate and acute bronchial asthma in children under the age of 15 years	Asthma (0 to 14)	7963.77834457529 (13656.3525907285 to 51.6514027019469)
Type I diabetes mellitus	Diabetes mellitus type 1 (all ages)	7429.36242517253 (9651.21392599779 to 0)
Testicular cancer in people aged 15 years and older	Testicular cancer (20 plus)	6425.06386606812 (7673.24210673214 to 0)
Refractory epilepsy in children between the ages of 1 and 15 years	Idiopathic epilepsy (0 to 14)	5569.06914520869 (11130.3966112371 to 105.052591863323)
Heavy consumption or low to moderate risk of addiction to alcohol and drugs in people under the age of 20 years	Substance use disorders (<20 years)	5312.55244366324 (7461.50867463031 to 698.314690531639)
Treatment for the eradication of *Helicobacter pylori*	Gastritis and duodenitis (all ages)	3833.44230358697 (5407.26107597058 to 31.0664601228219)
Differentiated and medullary thyroid cancer in people aged 15 years and older	Thyroid cancer (20 plus)	3524.95356842812 (3925.21814487822 to 0)
Treatment for benign prostatic hyperplasia in symptomatic people	Benign prostatic hyperplasia (all ages)	3136.15726142161 (4876.83538193793 to 0.00194715937414714)
Surgical treatment for chronic lesions of the mitral and tricuspid valves in people aged 15 years and older	Non-rheumatic degenerative mitral valve disease (20 plus)	2321.91890136389 (2772.42279203503 to 0.00640083277215629)
Refractive errors in people aged 65	Refraction disorders (65 to 89 years)	2267.63302256029

years and older		(3291.72011313406 to 4699.20120851181)
Relapsing-remitting multiple sclerosis	Multiple sclerosis (all ages)	2192.67301330785 (2860.98207134884 to 0.000916848432839514)
Cholecystectomy for the prevention of gallbladder cancer in people between the ages of 35 and 49 years	Gallbladder and biliary diseases (25 to 49)	2061.70765625961 (2857.83007158215 to 7.89297979047496)
Outpatient care for Acute Respiratory Infection (ARI) in children under the age of 5 years	Upper respiratory infections (under 5)	1783.98509162834 (2869.84870415435 to 53.7737581195539)
Cleft lip and palate	Orofacial clefts (0 to 14)	66.8835836316003 (109.496663940256 to 17707.9341422313)

Appendix C.2. Excluded GES conditions

GES condition	Reason for exclusion
Primary or essential arterial hypertension in people aged 15 years and older	Matching condition not found in GBD 2019 data
Hemophilia	Matching condition not found in GBD 2019 data
Cystic fibrosis	Matching condition not found in GBD 2019 data
Juvenile idiopathic arthritis	Matching condition not found in GBD 2019 data
Hypothyroidism in people aged 15 years and older	Matching condition not found in GBD 2019 data.
Systemic lupus erythematosis	Matching condition not found in GBD 2019 data
Bilateral hearing loss in people aged 65 years and older requiring hearing aids	Matching condition not found in GBD 2019 data
Diabetic retinopathy	Matching condition not found in GBD 2019 data
Nontraumatic rhegmatogenous retinal detachment	Matching condition not found in GBD 2019 data
Scoliosis surgery for people under the age of 25 years	Matching condition not found in GBD 2019 data
Osteosarcoma in people aged 15 years and older	Matching condition not found in GBD 2019 data
Strabismus in children under the age of 9 years	Matching condition not found in GBD 2019 data
Hip dysplasia	Matching condition not found in GBD 2019 data
Treatment for moderate, severe and profound hearing loss in children under four	Matching condition not found in GBD 2019 data
Serious polytrauma	GES condition does not match with a unique cause in GBD 2019 data
Lymphomas in people aged 15 years and older	GES condition does not match with a unique cause in GBD 2019 data

Spinal dysraphism	Matching condition not found in GBD 2019 data
Surgical treatment of chronic lesions of the aortic valve in people aged 15 years and older	Matching condition not found in GBD 2019 data
Surgery for herniated nucleus pulposus	Matching condition not found in GBD 2019 data
Severe burns	Matching condition not found in GBD 2019 data
Serious eye trauma	Matching condition not found in GBD 2019 data
Impulse and conduction disorders in people aged 15 years and older who require a pacemaker	Matching condition not found in GBD 2019 data
Respiratory distress syndrome in the newborn	Matching condition not found in GBD 2019 data
Bilateral sensorineural hearing loss in premature babies	Matching condition not found in GBD 2019 data
Bronchopulmonary dysplasia in premature babies	Matching condition not found in GBD 2019 data
Retinopathy in premature babies	Matching condition not found in GBD 2019 data
Moderate and serious traumatic brain injury	Matching condition not found in GBD 2019 data

Appendix C.3. Excluded GES programs

Orthosis (or technical help) for people aged 65 years and older
Pain relief and palliative care for advanced cancer
Analgesia in childbirth
Integral dental health care for girls and boys aged 6 years old
Outpatient treatment for odontological emergencies
Integral dental health care for 60 year old adults
Integral dental health care for pregnant women
Secondary prevention of end-stage chronic kidney disease

PRISMA-P (Preferred Reporting Items for Systematic review and Meta-Analysis Protocols) 2015 checklist: recommended items to address in a systematic review protocol*

Section and topic	Item No	Checklist item	Page
**ADMINISTRATIVE INFORMATION**			
Title:			
Identification	1a	Identify the report as a protocol of a systematic review	1
Update	1b	If the protocol is for an update of a previous systematic review, identify as such	N/A
Registration	2	If registered, provide the name of the registry (such as PROSPERO) and registration number	N/A
Authors:			
Contact	3a	Provide name, institutional affiliation, e-mail address of all protocol authors; provide physical mailing address of corresponding author	2
Contributions	3b	Describe contributions of protocol authors and identify the guarantor of the review	15
Amendments	4	If the protocol represents an amendment of a previously completed or published protocol, identify as such and list changes; otherwise, state plan for documenting important protocol amendments	N/A
Support:			
Sources	5a	Indicate sources of financial or other support for the review	N/A
Sponsor	5b	Provide name for the review funder and/or sponsor	15
Role of sponsor or funder	5c	Describe roles of funder(s), sponsor(s), and/or institution(s), if any, in developing the protocol	15
**INTRODUCTION**			
Rationale	6	Describe the rationale for the review in the context of what is already known	3-4
Objectives	7	Provide an explicit statement of the question(s) the review will address with reference to participants, interventions, comparators, and outcomes (PICO)	5
**METHODS**			
Eligibility criteria	8	Specify the study characteristics (such as PICO, study design, setting, time frame) and report characteristics (such as years considered, language, publication status) to be used as criteria for eligibility for the review	5-6
Information sources	9	Describe all intended information sources (such as electronic databases, contact with study authors, trial registers or other grey literature sources) with planned dates of coverage	6-7
Search strategy	10	Present draft of search strategy to be used for at least one electronic database, including planned limits, such that it could be repeated	Appendix A

For peer review only

Study records:			
Data management	11a	Describe the mechanism(s) that will be used to manage records and data throughout the review	7-8
Selection process	11b	State the process that will be used for selecting studies (such as two independent reviewers) through each phase of the review (that is, screening, eligibility and inclusion in meta-analysis)	7
Data collection process	11c	Describe planned method of extracting data from reports (such as piloting forms, done independently, in duplicate), any processes for obtaining and confirming data from investigators	7-8
Data items	12	List and define all variables for which data will be sought (such as PICO items, funding sources), any pre-planned data assumptions and simplifications	7-8
Outcomes and prioritization	13	List and define all outcomes for which data will be sought, including prioritization of main and additional outcomes, with rationale	11
Risk of bias in individual studies	14	Describe anticipated methods for assessing risk of bias of individual studies, including whether this will be done at the outcome or study level, or both; state how this information will be used in data synthesis	13
Data synthesis	15a	Describe criteria under which study data will be quantitatively synthesised	
	15b	If data are appropriate for quantitative synthesis, describe planned summary measures, methods of handling data and methods of combining data from studies, including any planned exploration of consistency (such as I^2, Kendall's τ)	
	15c	Describe any proposed additional analyses (such as sensitivity or subgroup analyses, meta-regression)	
	15d	If quantitative synthesis is not appropriate, describe the type of summary planned	8, 13, 14
Meta-bias(es)	16	Specify any planned assessment of meta-bias(es) (such as publication bias across studies, selective reporting within studies)	N/A
Confidence in cumulative evidence	17	Describe how the strength of the body of evidence will be assessed (such as GRADE)	13-14

*** It is strongly recommended that this checklist be read in conjunction with the PRISMA-P Explanation and Elaboration (cite when available) for important clarification on the items. Amendments to a review protocol should be tracked and dated. The copyright for PRISMA-P (including checklist) is held by the PRISMA-P Group and is distributed under a Creative Commons Attribution Licence 4.0.**

From: Shamseer L, Moher D, Clarke M, Ghersi D, Liberati A, Petticrew M, Shekelle P, Stewart L, PRISMA-P Group. Preferred reporting items for systematic review and meta-analysis protocols (PRISMA-P) 2015: elaboration and explanation. BMJ. 2015 Jan 2;349(jan02 1):g7647.

For peer review only
